# Study protocol for a pilot evaluation of Talk With Me Baby (TWMB) for Well-Child Care: A primary care-integrated language promotion intervention in rural and underserved clinics

**DOI:** 10.1371/journal.pone.0353423

**Published:** 2026-07-24

**Authors:** Brenda Salley, Margaret Jaynes, David Huss, Kristina Foster, DeAnn Hubberd, Kyle Pohl, Stephen Adkison, Phyllis Nader, Jaime Baldner, Melody Greer, Jing Jin, Linda Y. Fu, Preetha Abraham, Edward F. Ellerbeck

**Affiliations:** 1 Department of Pediatrics, University of Kansas Medical School, Kansas City, Kansas, United States of America; 2 Division of Pediatric Neurology and Child Development, West Virginia University, Morgantown, West Virginia, United States of America; 3 Department of Biostats, University of Arkansas for Medical Sciences, Little Rock, Arkansas, United States of America; 4 Department of Pediatrics, Alaska Native Tribal Health Consortium, Anchorage, Alaska, United States of America; 5 National Institutes of Health Environmental Influences on Child Health Outcomes (ECHO) Program, Washington, DC, United States of America; 6 Department of Population Health and Internal Medicine, University of Kansas Medical School, Kansas City, Kansas, United States of America; Hacettepe University: Hacettepe Universitesi, TÜRKIYE

## Abstract

A child’s home language environment (HLE) refers to the quality of early language exposure and linguistic interactions with caregivers. A robust HLE is a primary public health consideration, as it positively influences a child’s early- and long-term academic, health and economic outcomes. Talk With Me Baby for Well-Child Care (TWMB) was developed to encourage a robust HLE within routine pediatric healthcare. While this approach has been deployed in a variety of settings, TWMB has not been rigorously evaluated in rural and underserved primary care settings. This paper describes a research protocol to evaluate the preliminary efficacy of TWMB in clinics serving rural or underserved families. At least 66 child-caregiver dyads will be recruited over 5 months (child age two to six months at enrollment) from two primary care clinics. All participants will receive the TWMB intervention during routine WCC visits at participating clinics during a 12-month period. Participants will complete baseline and post-intervention assessments of their HLE. Preliminary efficacy outcomes include changes in the caregivers’ language-promotion behaviors (as measured by Language Environment Analysis normative scores for daily Conversational Turn Count between the adult and child and daily Adult Word Count) from baseline to 12 months post-intervention. This trial has been prospectively registered as NCT06479278 on 2024-06-26.

## Introduction

Children’s home language environment (HLE) —the quality and quantity of early language input from parents and caregivers—predicts their language development, literacy, and academic success [1–10]. It is the single strongest predictor of a child’s vocabulary development by three years of age [8], and early language-rich interactions with a caregiver support critical brain development necessary for a child’s optimal language and long-term outcomes [3,7–9]. Neural networks for language development are present before birth, and language input influences the brain’s neural circuitry long before infants speak their first words [11,12]. Peak synapse growth responsible for language occurs at six months and is influenced by a child’s language environment [13,14]. Children who experience more language-rich interactions with parents and caregivers display more robust early neural connectivity and activation in specific brain regions that support language and literacy [15–18]. Notably, it is not overheard speech (i.e., simply hearing words from adults, television, or video), but rather speech directed to a child that facilitates their language development [8,19]. The effects of the HLE also become magnified over time: a child’s vocabulary at three years of age predicts later academic achievement [20–24], which in turn predicts their long-term health and economic outcomes [20–23].

A robust literature has documented that differences in the quality of language-building opportunities for young children, and parent knowledge about their importance, are found across all sociodemographic strata [7–9,25–30]. However, HLE differences are often linked to families’ socioeconomic status, parent education, access to child care, economic resources and other social determinants of health [7,29,31,32]. Importantly, the last three decades have yielded clear evidence-based language-promotion strategies that can optimize early parent-child language interactions. Parentese, a form of infant-directed speech characterized by higher pitch and elongated vowels, is one key strategy. A second strategy, responsivity and conversational turns, involves continuous verbal exchanges in which caregivers and children provide contingent responses to each other’s preceding utterances. A third key strategy, narrating and expanding, entails modeling language through description of the child’s environment and what the caregiver/child are doing together and by repeating and adding new words to a child’s utterances [33–35]. Meta-analyses of these strategies in randomized controlled trials have also yielded evidence of strong, positive effects on parents’ language-promotion behaviors and children’s receptive and expressive language outcomes [33,34]. However, existing evidence-based interventions in home-visiting and childcare settings have not had the reach needed to narrow population-level language disparities [36–38]. The primary barrier has been how to scale these interventions to large populations of parents and caregivers, especially underserved families who need this information most [37,38].

Notably, young children living in rural or underserved areas disproportionately experience multiple risk factors that impact the HLE, including higher rates and higher levels of poverty, more persistent poverty and reduced access to key health and early childhood services [39–47]. Families in rural and underserved areas face persistent structural barriers, including limited access to early intervention services, fewer early childhood education programs, provider shortages, and greater travel distances to care. These constraints reduce exposure to evidence-based language-promotion resources and highlight the need for scalable interventions embedded within primary care. Embedding language-promotion strategies within well-child care may therefore be particularly important in rural settings, where primary care is often the most consistent point of developmental support.

Primary care offers a unique opportunity to deliver a universal, low-cost, scalable language promotion intervention to families with infants and toddlers, given that 91–98% of all families with infants and toddlers access primary care services [48]. While the American Academy of Pediatrics (AAP)/ Bright Futures anticipatory guidance objectives identify the importance of spoken language and education to promote language development in young children, standard of care anticipatory guidance does not give primary care providers any practical strategies for delivering education to enrich caregiver-child language interactions and the HLE [38,49,50].

Talk With Me Baby (TWMB) was launched as a statewide initiative in Georgia to promote “language nutrition” for children into public health care and related systems [51]. The term language nutrition refers to the use of language that is sufficiently rich in engagement, quality, quantity, and context to nourish a child neurologically, socially, and linguistically. As a public health model, TWMB was designed to educate healthcare teams and communities about the importance of early language interactions and has since been disseminated nationally and internationally.

TWMB was subsequently adapted for structured integration within routine pediatric well-child care (WCC), embedding language-promotion coaching directly into existing anticipatory guidance workflows [52]. This WCC-integrated model leverages repeated clinical touchpoints and trusted provider-family relationships to deliver evidence-based strategies – parentese, conversational turn-taking, and narration – within standard visit timeframes. Similar to literacy-focused interventions such as Reach Out and Read [53], TWMB in WCC capitalizes on the near-universal reach of primary care during early childhood, including up to eight WCC visits by 15 months. The model introduces a systematic delivery structure and practical tools that support in-visit education, modeling and coaching, equipping caregivers with specific, evidence-based strategies to apply with their child during daily activities. Consistent with the TWMB framework, the approach is culturally adaptable, emphasizing caregivers’ use of their primary home language(s) and integration of language-promotion strategies into existing family routines and practices.

The present study builds on the previously described WCC-integrated TWMB model and evaluates its preliminary efficacy in rural and underserved primary care settings, where structural barriers and resource constraints may influence delivery and impact. Although TWMB is grounded in evidence-based language-promotion strategies, it has not been rigorously evaluated in these settings. The objective of this pilot study is to assess preliminary efficacy of TWMB on caregiver language promotion behaviors, specifically changes in adult word count and conversational turn count for changing caregiver language-promotion behaviors. Primary outcomes are adult word count and conversational turn count, indexing quantity of input and interactional reciprocity, respectively. This pilot estimates the proportion of caregivers demonstrating improvement from baseline (Week 1) to post-intervention (Week 48) and tests whether this proportion exceeds a prespecified null benchmark of 50% (defined as ≥10% increase in AWC or CTC percentile scores). Child language outcomes are conceptualized as downstream effects and will be examined in a future adequately powered randomized controlled trial.

## Materials and methods

### Study design

This is a multi-site study designed to investigate the preliminary efficacy of evidence-based language promotion embedded in routine pediatric primary care, entitled “Talk With Me Baby (TWMB).” The SPIRIT (Standard Protocol Items: Recommendations for Interventional Trials) guidelines [54] were used to develop the reporting of the current study (see S1 File). The study duration is approximately 24 months with participant recruitment spanning about 5 months. This pilot trial will take place in two pediatric primary care clinics in two states (Kansas and West Virginia) within the IDeA States Pediatric Clinical Trials Network (ISPCTN). The study will evaluate preliminary efficacy of TWMB on caregiver language-promotion behaviors when delivered under routine conditions in rural and underserved primary care clinics. Within each clinic, two providers or provider/care team members will be trained to deliver TWMB during the study intervention period with all children 4–36 months of age, regardless of whether the family is enrolled in the study. This is because TWMB is an intervention designed to improve clinical care for all children within primary care well-child visits. We included TWMB delivery at WCC visits for children ages 4–36 months of age because this is the target age group for the TWMB intervention, although for feasibility purposes in this pilot we will only enroll children aged two to six months (+30 days) old. We include this age range for the child participants (rather than all children 4–36 months of age) to minimize variability due to development within the sample.

After the recruitment phase, the study will include an intervention run-in period (i.e., a two-week pre-trial period) during which providers will deliver TWMB during WCC visits. This period will ensure that TWMB can be implemented consistently and with acceptable adherence. Although there is no universal standard for acceptable fidelity for behavioral interventions, positive effects are typically observed at adherence levels ranging from 60–80% [55–58]. In this pilot study, the study team will apply a 75% criterion for acceptable adherence to delivery of critical TWMB elements during a WCC visit, consistent with our previous data [52].

The pre-post intervention design allows us to evaluate participant completion of critical measures that will be used in the future, large-scale randomized controlled trial. Importantly, this design also allows us to estimate the expected change in caregiver language-promotion skills in response to the intervention. Since TWMB targets caregiver language behaviors, the design includes a pre-post intervention assessment of the HLE. As per emerging guidance on pilot studies prior to high-quality large-scale efficacy trials, the current study design does not include a control group [59–61], given the high likelihood of an underpowered sample in this pilot to detect meaningful differences in the endpoints; these differences would be evaluated in a future large-scale randomized controlled trial. By focusing the pilot on delivering TWMB in both clinics, we can maximize the number of dyads and obtain a better estimate of variability in intervention response. This approach ensures we are not allocating resources to conduct underpowered difference tests between conditions for an untested intervention in this setting.

### Primary care clinics

Clinics must meet all the following criteria to participate in the study:

- Provide primary pediatric general healthcare, including at least 300 unique WCC visits to children with the age range from 2 to 36 months of age during the last 12 months.
- Have at least two full-time healthcare providers or one provider plus care team member(s) (i.e., physicians, physician assistants, nurse practitioners, nurses, nursing assistants, patient care technicians) who agree to participate in the study trial.
- Have the capacity to generate patient lists and appointment schedules (e.g., from electronic health records [EHR] or billing database structure) to identify potential participants within the eligible age range.
- Meet one or both of the following criteria for the patient population served: 1) ≥40% from a rural zip code as defined by rural-urban community area (RUCA) code ≥4; and 2) ≥40% with Medicaid insurance or uninsured.
- Agree to have all providers who deliver WCC and their care team members participate in TWMB training.

### Study participants

The study population includes parent-child dyads (up to 66) consisting of one caregiver and their child.

#### Inclusion criteria.

A potential caregiver must meet all the following criteria to participate in the study:

- Be the age of majority or older, as defined by the state of residency.
- Able to complete study measures in English.
- Have the legal authority to consent to participate for themselves and to consent on behalf of their eligible child.

A potential child participant must meet all the following inclusion criteria to be enrolled in the study:

- Receive WCC at a participating clinic.
- Be two to nine months (+ 0–30 days) old at enrollment.
- Born at full term (≥ 37 weeks gestation).
- Born in a singleton birth (i.e., was the only child delivered during the birth).

Although TWMB is implemented broadly within participating clinics to maintain consistent provider practice, the sample in this pilot is restricted to infants aged 2–6 months at enrollment. This narrower analytic cohort reduces developmental variability and ensures comparable baseline measurement prior to intervention exposure.

#### Exclusion criteria.

Potential caregiver participants will be excluded if they:

- Have cognitive impairment or a visual or hearing impairment known to the clinic that limits their ability to make decisions about participating or engaging with the assessments.
- Do not live with child or spend at least two full days (i.e., at least 48 hours) per week with the child.
- Do not plan for the child to continue receiving services at the participating clinic for at least 12 months.

Potential child participants will be excluded if they:

- Have significant developmental delay or cognitive, visual, or hearing impairment known to the clinic.
- Previously attended a WCC visit at a participating site prior to enrollment that occurred during the TWMB trial.

#### Recruitment and consent.

**Fig 1:**
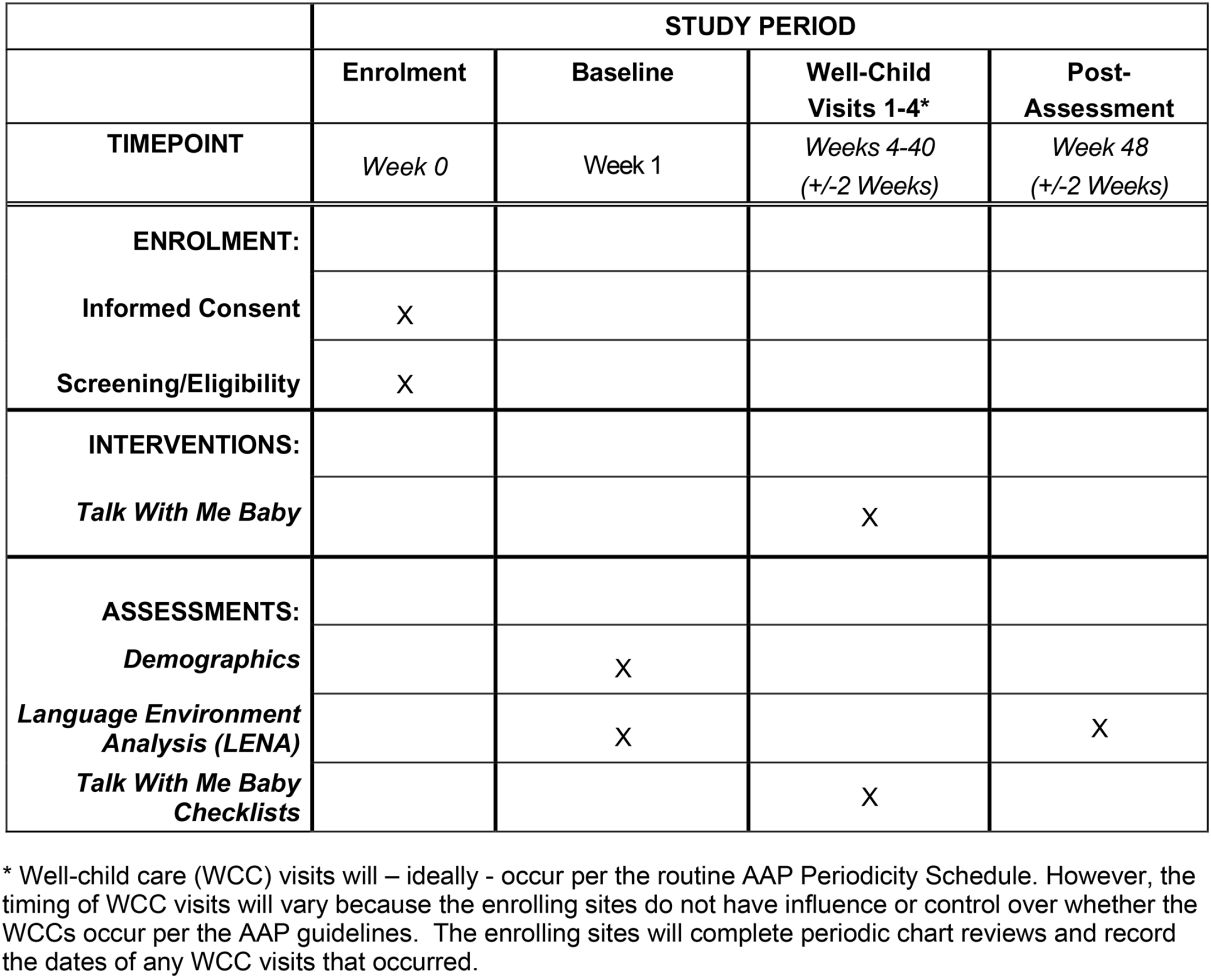
Schedule of Activities.

The study schedule of activities is provided in Fig 1. Eligible caregiver–infant dyads will be recruited consecutively from participating clinics during the recruitment period until the target sample size is reached. To ensure dyads are naïve to the TWMB intervention, we will enroll our sample of dyads prior to clinic providers receiving their TWMB training. This approach is common in behavioral intervention trials and important in situations where the intervention training may change the standard-of-care practices (i.e., in this case, it could change the anticipatory guidance providers give to families related to language milestones and language development prior to enrolled dyads completing their baseline HLE assessment).

Before recruitment begins, all patients will be mailed an Institutional Review Board (IRB) approved opt-out letter allowing them to express their desire to opt out of sharing protected health information for prerecruitment screening purposes. Information about how to join the study will also be included in the letter. We will then contact potential participants by phone or during clinic visits to complete screening surveys with caregivers. We will first contact caregivers of children who have Medicaid insurance or are uninsured as a strategy to ensure sufficient enrollment of potentially medically underserved children. Study staff will introduce the TWMB intervention, explain the study assessment component, and obtain consent to participate in the study. After written consent is obtained (see Appendix A in S1 File), study baseline assessments will be completed. Throughout the recruitment period, the study team will assess accrual at least twice monthly to decide whether it is necessary to adjust recruitment strategies. For example, if accrual rate is lower than expected, the study team will update recruitment materials to address areas of concern for families related to recruitment. The recruitment phase will begin February 3, 2025 and is expected to continue through July 30, 2025. Data collection is expected to continue through August 2026.

#### Engagement and retention strategies.

The research team will provide clinics and participating providers regular study updates, clinic-specific feedback on progress, and messaging about the study. Clinics and participating providers will have the opportunity for at least monthly meetings, in addition to as-needed or as-requested check-ins with the study team, to answer questions, provide support and address implementation challenges that arise. Support can be provided via e-mail, phone, or video-based communication platforms, per the preference of the clinics and participating providers. Providers (TWMB Clinic Champions) will also receive compensation for their time completing TWMB training and participating in the trial.

To engage and retain families who may be hesitant to engage in research, we will use strategies that have been recommended in previous clinical trials with families from wide-ranging geographic areas and diverse settings (e.g., we will engage clinic providers as trusted community partners to advertise the study; we will engage research staff who are culturally sensitive, and research staff will contact potential participants actively and repeatedly) [62]. We will use IRB-approved contact methods/language, which may include phone or text message reminders and engagement touchpoints [63]. We will also utilize local Community Advisory Boards to provide feedback on recruitment and retention materials. To address barriers related to caregiver language and literacy levels, we will ensure study participant-reported assessment tools are written at an accessible reading level and offer completion by interview. All study materials are available in printed format, and study staff provide phone-based guidance to ensure accessibility for families with limited internet or smartphone access. We will employ multiple contact modalities for communicating with participants throughout the study, including (but not limited to) e-mail, phone calls, and text messages. We will also contact participants with touchpoint communication (i.e., e-mail, text, phone) periodically during the trial to share information about the project. Child-caregiver dyads will also receive compensation via prepaid gift cards for their time completing baseline and post-intervention measures.

### Intervention

The TWMB intervention creates a clinic workflow structure for primary care providers and their care teams to deliver routine anticipatory guidance following AAP guidelines related to language development at specific intervals (detailed in Bright Futures Guidelines) [50,64–66] during routine WCC for all families with children 4–36 months of age. TWMB is intended to function within existing workflows rather than require additional staffing resources. TWMB will be used by participating providers at every WCC with all children 4–36 months of age during the intervention period, regardless of whether the family is enrolled in the study. Providers and their care teams will be trained to deliver TWMB prior to the start of the trial. Providers will apply an “I do – We do – You do” structure within the standard of care WCC time frame. This combination of brief education, modeling, and coaching [34,67] is an evidence-based approach enhancing caregiver knowledge and language behaviors. The “You do” stage involves brief, structured in-visit rehearsal guided by the TWMB Checklist, with caregivers practicing a strategy and receiving feedback. It serves as behavioral activation, with sustained practice occurring in daily routines at home and reinforced across visits. Although each TWMB interaction adds approximately 2–3 minutes to a well-child visit, the intervention is delivered repeatedly across multiple visits during early infancy, resulting in cumulative exposure. This repeated, brief coaching model aligns with evidence supporting the effectiveness of low-intensity, provider-delivered behavioral interventions when integrated into routine care. Our Provider Advisory Board reviewed the TWMB workflow and agreed it was readily achievable and of high value. This workflow was also implemented successfully in our preliminary work.

Specifically, the TWMB primary intervention components in this workflow include having the provider: (1) Model by showing how to use key language promotion strategies with a child and pointing out how the child is responding; (2) Educate by sharing why language promotion is important for school readiness and future health; (3) Coach by encouraging the caregiver to practice language promotion during the visit and giving feedback; and (4) provide a Language Nutrition Prescription for the caregiver(s) to use language promotion during daily activities with the child. Rather than prescribing specific activities, TWMB encourages caregivers to embed strategies within their typical daily routines (e.g., during meal preparation, outdoor activities, or caregiving tasks), which may differ across rural and urban contexts. In rural settings, where travel distances are longer and daily routines may involve agricultural or outdoor work, strategies can be incorporated into caregiving during transportation, outdoor tasks, or shared family activities.

TWMB focuses on three evidence-based language-promotion strategies. *Parentese* is a speaking style with young children characterized by melodic tone, varied intonation; higher pitch; elongated consonants and vowels; short, simple, and repeated; and precise pronunciation and grammar. *Take Turns* is noticing what a child is interested in and how a child is responding; following in to talk about what a child is interested in; taking turns and keeping the conversation (or back and forth sounds) going. *Narrate Your Day* is talking about what is happening around a child, about what the child is doing, and about what you and the child are doing together. Importantly, TWMB is facilitated by specific intervention tools—the TWMB Checklist and Language Nutrition Prescription. These tools facilitate TWMB delivery by serving as cues to action for providers and their care teams and provide educational resources for caregivers to take home. The TWMB Checklist is also a structured fidelity tool used to document delivery of core intervention components during well-child visits. Fidelity is defined as completion of ≥75% of required checklist elements per visit.

### Provider training

#### TWMB training.

For each clinic, TWMB training for providers and care teams will occur after the initial 4-month recruitment window, or after recruitment and baseline assessments are completed and the target sample is met for the clinic, whichever occurs first. TWMB training involves didactic and practice-based instruction that includes:

- A brief review of the science behind Talk With Me Baby.
- Focused instruction with video examples and modeling on how to deliver components of TWMB intervention (described below).
- Practice and role play with the trainer on how to deliver TWMB across child ages and different families and how to use the TWMB Checklist and Language Nutrition Prescription (described below).

Training will be sufficient to ensure each provider demonstrates acceptable adherence to delivery of critical TWMB elements (completion of 75% of TWMB Checklist components) during WCC role play to complete training. Training will be offered a minimum of two different times per clinic to accommodate providers and care teams and will be recorded for later use as needed. All clinic providers and care teams will be invited to attend TWMB training. However, only two providers will be selected as participating “Clinic Champions.”

#### Run-in period.

After TWMB training, we will complete a 2-week run-in period to ensure that participating providers are delivering TWMB with sufficient frequency during their WCC visits (at least 70% of sampled ≤ 36-month WCC visits) and with sufficient quality, delivering critical TWMB elements during each WCC visit (at least 75% of primary intervention components) prior to start of the intervention. Provider adherence will be assessed via the TWMB Checklist (described above). The checklist functions as a structured delivery log rather than a subjective coding instrument; therefore, formal inter-rater reliability testing is not conducted. Our previous work has established that the TWMB checklist, which is co-completed by the clinician and caregiver, is highly reliable (96% agreement with coded WCC visits) [52].The site coordinator will review and log all TWMB Checklists collected to compare to our target of 70% uptake. In preliminary implementation work, providers consistently met these criteria within approximately two weeks. However, clinics can complete a second training and run-in period if needed to achieve this criterion.

### Study measures

#### Participant demographic and clinical data.

The study staff will send caregivers an electronic link (by e-mail or text, depending on caregiver preference) to complete demographics questionnaires or will complete with caregivers by phone. Research staff will follow up with reminders for completion (after the initial invitation). These methods (which we have used successfully in previous large-scale longitudinal studies) are feasible given national data indicate 85% of adults have smartphones (77% have broadband) [68].

#### Caregiver language promotion behaviors.

Caregivers will complete Language Environment Analysis (LENA) assessments at baseline (Week 1) and post-intervention (Week 48). LENA, the research standard for measuring talk with children, provides a quantitative measure of the HLE. LENA has been widely used in research and is feasible for full-day recording, with children wearing the device in a vest pocket during typical daily routines. LENA uses a small recorder (“talk pedometer”) that children wear in a vest pocket for a day at a time [69]. It meets U.S. and international safety standards, does not transmit, and has the same low-power processors as hearing aids. LENA software processes the audio into talk data (with built-in fidelity measures). Study staff will mail the LENA recorder and vest to the family; alternatively, the LENA recorder may be provided to the family at a clinic visit. Study staff will then complete a short phone call with the family to walk through how to use the device and answer any questions (simple instructions are also provided with the device, and brief video instructions will be sent electronically). Families will be asked to complete a day-long recording during a period when the child is in the home setting (i.e., not at daycare), turning on and putting on the device at the start of the day when the child first wakes up and leaving it on for 12–16 hours. The LENA device can remain turned on and be taken off and left nearby during baths or naps if needed. The device automatically turns off when the maximum recording time is reached. Families will complete a recording on two separate days during Baseline (i.e., Phase 1). The families will be sent 2 LENA devices and 2 vests (1 pair for each recording day). After completing the baseline recording, the family will mail the LENA recorder and vest back to the study team in a prepaid mailer or drop the device off at the clinic.

To continue in the study to the second LENA assessment (phase 2), the scores for child-caregiver dyads for the first LENA assessment must be ≤ 75^th^ percentile, compared to age-referenced normative data. Phase 2 participants will complete a recording on two separate days again during the post-intervention assessment phase. If the LENA data do not meet LENA fidelity benchmarks to extract a usable recording, the site study team will send the LENA devices back to the family in a second attempt to collect an additional sample. Complete LENA data will be defined as follows: at least one usable recording (i.e., usable recording from one day of the two possible recording days) is collected from caregiver participants within two attempts (to account for potential errors in recording). LENA recordings are processed using automated software to generate quantitative language metrics; raw audio files are not accessed or reviewed by the study team and are not retained as part of the study dataset.

#### Clinic attendance.

During the intervention period, the study team will use the clinics schedules to log participants’ attendance at their well-child visits.

### Outcomes

#### Primary outcome.

This pilot study evaluates proximal caregiver language promotion behavior change as the primary endpoint. Child language development is conceptualized as a downstream outcome and is not directly assessed in this protocol. Primary outcomes include LENA age-referenced normative scores for daily Conversational Turn Count (CTC) between the adult and the child and daily Adult Word Count (AWC) directed to the child [70]. Consistent with the proposed mechanism of change, adult word count is used to index quantity of language input, whereas conversational turn count serves as an indicator of contingent interactional reciprocity within the home language environment.

#### Exploratory analyses.

We will examine barriers to enrollment, including our ability to contact caregivers, the proportion of caregivers that consent to participate in the study, enrollment of caregiver-child dyads, and caregivers’ willingness to complete baseline assessments. In addition, characteristics related to attrition for caregiver and provider participants will be examined. Finally, we will also examine associations of differences in change in LENA scores with caregiver characteristics (e.g., low or high baseline LENA scores, education level, SES) and child vocalizations.

### Statistical considerations

#### Sample size.

The primary endpoint is a binary variable defined by reaching at least a 10% increase in AWC or CTC percentile scores from Baseline (Week 1) to Post-intervention (Week 48). This increase is based on previous language-promotion intervention studies using LENA, which reported statistically significant gains (for intervention versus control) for parent language-promotion behaviors ranging from 10–30% [71–76]. A 10% increase in AWC or CTC percentile scores is consistent with prior language-promotion intervention studies demonstrating meaningful improvements in caregiver language input. The study is powered to detect whether the proportion of participants demonstrating at least a 10% increase in AWC or CTC exceeds a null benchmark of 50%. A target improvement rate of 70% was used as a planning parameter for sample size estimation, based on prior studies reporting small-to-moderate effects. This threshold is not interpreted as an expected outcome but as a benchmark for evaluating observed variability. We plan a total accrual of 66 child-caregiver dyad participants into the study. With an estimated attrition rate of 20%, we expect that a total of 53 enrolled child-caregiver dyad participants will complete the study, and hence the effective sample size is 53. Using a two-sided exact test with a significance level of 0.05, we are able to reach 85% power to reject a null rate of improvement at 50%, should the targeted rate of improvement be reached.

#### Statistical analysis.

All numerical variables will be summarized using mean ± standard deviation and median (minimum, maximum). All categorical variables will be summarized using frequency (in %). Numerical variables will also be inspected for outliers and empirical distributions during data review. Descriptive statistics of the primary endpoints will be reported by caregiver-child demographics (such as race), Medicaid status (yes/no), and WCC visit age (e.g., 2, 4, 6, 9, 12, 18-month WCC visit). The primary endpoint of improvement in caregiver language-promotion behaviors (as measured by LENA) will be summarized using frequency (in %). A 95% confidence interval will be constructed using a Clopper-Pearson exact method. Alternatively, a hypothesized null rate can be tested using a two-sided exact test at a significant level of 0.05. Descriptive statistics will be used in exploratory analyses. We will use analysis of variance (ANOVA) models or linear regression models to examine associations of differences in change in LENA and caregiver characteristics.

### Data management and monitoring

#### Data management.

This will be completed by the ISPCTN Data Coordinating Center (DCOC). The clinical database management system (CDMS) will be built using a validated electronic data capture system, with an audit trail, that is fully compliant with 21 CRF Part 11. The CDMS will be designed, developed, validated and managed by the DCOC. Edit checks will be developed by the DCOC to check data values for inconsistencies, including missing values, values out of range, invalid and illogical data. The electronic data capture (EDC) system will have a discrepancy management system to track the status of the electronic edit checks and allows for manually created queries. Status, Exception and Safety reports will be generated and distributed to the Protocol Team weekly to monitor the progress and outstanding data for the study.

#### Study monitoring.

Clinical sites will be monitored to ensure that site PIs, site coordinators, and research team members are protecting the rights and well-being of trial participants, that the reported trial data are accurate, complete, and verifiable, and that the conduct of the trial complies with the currently approved protocol and its amendments, with International Council on Harmonisation Good Clinical Practice, and with applicable regulatory requirement(s).

Study progress and safety will be reviewed monthly (and more frequently if needed) by the study investigators. Progress reports include patient recruitment, retention/attrition, and adverse events. Because this study is of an educational intervention, study-related adverse events (AEs) and serious adverse events (SAEs) are not expected. Given the minimal risk of the study, the study team will not solicit AEs or SAEs. However, the study team will provide all participants with a phone number to report AEs and SAEs. The study team will track severe AEs and SAEs that are potentially study related. All AEs recorded during the study period will be coded with the Medical Dictionary for Regulatory Activities (MedDRA) [77].

Safety oversight will be under the direction of a Data and Safety Monitoring Board (DSMB) composed of individuals with the appropriate expertise, including pediatrics, child development and biostatistics. Members of the DSMB will be independent from the study conduct and free of conflicts of interest, or measures should be in place to minimize perceived conflicts of interest. The initial DSMB meeting will occur before the start of the trial to discuss the protocol and the DSMB Charter, which includes data review in open and closed table shells, the Data and Safety Monitoring Plan, definition of a quorum, and guidelines for monitoring the study. The DSMB will operate under the rules of an approved charter that will be written and reviewed at the organizational meeting of the DSMB, and each data element that the DSMB needs to assess will be clearly defined. The DSMB will provide its input to the NIH. Following the initial meeting, the DSMB will meet at least twice a year thereafter, per the DSMB charter, to assess safety and study enrollment. Adverse events will be tabulated by type, severity and relatedness to study treatment at the event level and participant level for review by the DSMB at the site of DSMB meetings.

### Ethics approval and informed consent

This study, protocol, and all instruments, including the informed consent document, have been approved by the Human Research Ethics Committee (HRECV) of the University of Arkansas Medical Sciences (UAMS) (FWA00001119) on 12/4/2023. Participants will be parent-infant dyads. All parent participants will provide written informed consent prior to participation; they will also provide written informed consent for infant (minor) participation. The outcomes of this study will be published and presented at national and/or international conferences. All data presented will be de-identified. The resources developed in this study will be made publicly available. If the TWMB intervention demonstrates evidence of preliminary efficacy in rural/underserved areas, it will be evaluated in a large-scale randomized control trial and can be applied in routine well-child care to support child development. This trial has been registered at ClinicalTrials.gov (NCT06479278) and we will submit trial results to ClinicalTrials.gov.

## Discussion

The HLE is a primary public health consideration, conferring critical protection or risk for young children, and more strongly predicting early neurodevelopment and long-term health, academic, and economic outcomes than parent income, education, race, and ethnicity [3,7,8,15,20–23,26,78,79]. Young children in rural and underserved areas (i.e., those with lower access to early childhood services, such as Head Start and early intervention, such as Part C services) are particularly at risk for disparities in the HLE. They are more likely than children in urban areas to experience higher and more persistent levels of poverty and reduced access to early childhood services [39–46,80]. When parents and caregivers receive instruction on how to use language promotion it benefits the quality and frequency of their language interactions with their child, which in turn improves child outcomes [33,34,81]. However, evidence-based interventions for language promotion have not been effectively deployed at scale to reach large proportions of disadvantaged families, with cost and access cited as the main barriers [36–38]. Since 91–98% of families with infants and toddlers access primary care services, WCC visits may be an ideal vehicle for delivering low-cost, scalable intervention to these parents and caregivers [48].

The current pilot study aims to demonstrate the preliminary efficacy of embedding universal language promotion (TWMB) during routine well-child care. This pilot focuses specifically on proximal caregiver behavior change, namely adult word count and conversational turns, as the primary outcome. Child language development is conceptualized as a downstream outcome of improved caregiver language input and will not be directly evaluated in this preliminary study. Demonstrating change in this behavioral mechanism is a necessary precursor to conducting a fully powered randomized controlled trial examining child language outcomes. The innovation of this study lies not in modification of intervention content, but in evaluating whether a standardized, evidence-informed language promotion model can be effectively integrated into routine care within lower-resourced rural systems. Rural clinics face unique structural constraints, including limited staffing, higher patient burden, and reduced access to early childhood services. Demonstrating that TWMB can be implemented with fidelity and produce measurable changes in caregiver behavior under these conditions addresses a critical gap in the scalability of early language interventions. Although the pre–post design does not allow us to attribute change in caregiver language-promotion behavior solely to the intervention, this pilot serves to estimate the magnitude and variability of change in caregiver language-promotion behaviors under routine clinical implementation. These data are necessary to inform effect size assumptions, refine eligibility thresholds, and assess potential ceiling effects. Establishing signal strength in real-world rural clinics is a critical step before to a larger controlled study. Sustained delivery over time may be affected by competing clinical demands; fidelity monitoring is intended to mitigate drift, but this will be important to evaluate in larger pragmatic studies. Because outcome evaluation is restricted to infants aged 2–6 months at enrollment, findings may not directly generalize to older toddlers (18–36 months), and it will be important for future trials to evaluate TWMB across the full intended age range.

To our knowledge, this is the first study to explore the preliminary efficacy of language promotion intervention in primary care clinics serving rural and/or underserved children. This study extends the literature by testing whether a structured language-promotion framework can be implemented within routine primary care workflows in rural and underserved settings. Rather than evaluating a stand-alone parenting program, TWMB operates as a systems-level integration within well-child care, with potential for universal reach through an existing healthcare infrastructure. This study is a necessary step toward evaluating efficacy in a fully powered randomized trial.

This intervention provides a framework for embedding efficient, evidence-based tools to help healthcare providers deliver language promotion within routine anticipatory guidance that aligns with Bright Futures Guidelines [50,64–66]. This study will evaluate whether TWMB demonstrates preliminary efficacy for parents’ language promotion behaviors. Results of this trial will inform a future larger study to test TWMB efficacy for optimizing caregivers’ language promotion behaviors and benefitting child language development; it has the potential to be scaled to diverse settings that provide pediatric primary care. Further, demonstrating the efficacy of TWMB as a low-cost, scalable, and universal language-promotion approach has significant potential to change the standard of WCC and to benefit children’s long-term academic, health, and economic outcomes [44,82,83].

## Supporting information

S1 File SPIRIT Checklist. (DOC)

S2 File Study Protocol. (PDF)
