# Supplementary material for: Study protocol for a pilot evaluation of Talk With Me Baby (TWMB) for Well-Child Care: A primary care-integrated language promotion intervention in rural and underserved clinics
Source: PLoS One. 2026 Jul 24;21(7):e0353423. doi: 10.1371/journal.pone.0353423 (PMC13399447; doi:10.1371/journal.pone.0353423)
Supplement: S2 File — (PDF) [file pone.0353423.s002.pdf]

Talk With Me Baby:

Leveraging Well-Child Care to Enhance the Early Home Language Environment for Rural and Underserved Children (TWMB)

UAMS IRB Protocol Number: 276453

Version Number: V-08

Date: 12-August-2025

National Clinical Trial (NCT) Identified Number: NCT06479278

DCOC Principal Investigator:

Songthip Ounpraseuth, PhD
(ISPCTN DCOC at UAMS)

Principal Investigator:
Brenda Salley, PhD
University of Kansas Medical Center

Co-Investigators:
David Huss, MD
West Virginia University

Margaret Jaynes, MD
West Virginia University

Sponsor: ISPCTN DCOC

Funded by: NIH

Summary of Key Changes from Version 01 to Version 02:

Changes made in response to pre-review contingencies and email of 10/24/2023 (1:02 PM) from the UAMS IRB Director, Edith Paal.

Affected Section(s)	Summary of Revisions Made from V-01 to V-02	Rationale
6.1.4. Study Staff Training	Added that training requirements will apply to research staff "who are listed on the sites' delegation logs" (in 1st sentence of 1st paragraph)<br><br>Added entire paragraph "All study staff that are on a delegation log…." after the 2nd set of bullets.	Response to pre-review contingency:<br>• "Study Staff Training" -- Please add language describing what kind of basic human subject protection training clinic staff will receive.<br>NOTE that the study team is no longer planning on using a clinic that is NOT part a university (e.g., KUMC or WVU) system, so training requirements will fall under the corresponding university's training requirement per the university's human protections program.
8.1.1 Clinic Procedures Sub-section "Clinic Schedule of WCC Visits Collection Procedures"	Deleted the following paragraph:<br>We plan to use a Data Use Agreement (DUA) with each clinic. This will allow transfer of data including protected health information (PHI) from the clinic to the study team. This process has been used successfully in research studies with rural clinics to allow data sharing.[108] Comparable HIPAA compliant solutions for transfer of PHI preferred by the cIRB, or the local IRB and clinic may also be implemented in place of a DUA if appropriate	The study team no longer plans on using a clinic that is NOT part of one of the university systems. Clinics will be part of a major university (e.g., KUMC or WVU) system.
8.1.2 Caregiver Assessments and Procedures.<br>Subsection: Primary Endpoint: Caregiver Language-promotion behaviors (LENA)	Added entire 2nd full paragraph to this subsection. Paragraph starts with "The LENA recording system, i.e., LENA SP, that will be use …."	IRB director requested additional information (email of 10/24/2023) on whether or not actual words could be accessed (listened to) from the device.

Summary of Key Changes from Version 02 to Version 03:

Affected Section(s)	Summary of Revisions Made from V-02 to V-03	Rationale
2.3.1, Known potential risks	Deleted sentence:<br>*Caregiver participants will answer questions related to their child's development (i.e., on the demographics questionnaire presented by research staff), which may provoke some emotional stress or anxiety.*<br>Added to beginning of sentence of 2nd full paragraph:<br>*"If any concerns about their child's development arise, …"*	Re: deleted sentence:<br>Questions are no longer part of the study design.<br><br>RE: Addition:<br>Clarification
8.1.2, Caregiver Assessments and Procedures, subsection "Enrollment"	Minor wording corrections and added that surveys can be done in person.	Clarifications
8.1.2, Caregiver Assessments and Procedures, subsection "Primary Endpoint; Caregiver Language-Promotion Behaviors)	Added 3rd full paragraph to detail how system will be tested prior to enrolling actual study participants.	Added to ensure the team has IRB permission to complete systems testing with volunteers prior to initial enrollment of actual study participants.
10.1.1.2 Consent Procedures and Documentation; subsection Remote Consent	Added the highlighted wording to sentence in 1st paragraph:<br>*The remote consent process will parallel the consent processed used for typical in-person consenting; this includes ensuring there is a signed consent and HIPAA authorization prior to starting any study-related procedures*	Clarification to make sure study teams know that ICF and HIPAA signatures are required prior to starting study-related procedures. I.e., verbal authorization alone is not sufficient.
10.3 Glossary	Redefined "caregiver" to match that in ICF.	Consistency with ICF

Summary of Key Changes from Version 03 to Version 04:

Affected Section(s)	Summary of Revisions Made from V-03 to V-04	Rationale
Cover page	Changed overall DCOC PI<br>FROM: Jessica Snowden, MD<br>TO: Songthip Ounpraseuth, PhD	Jessica Snowden, MD, left UAMS/DCOC at the end of August 2024 and Songthip Ounpraseuth, Ph.D., is the new DCOC (operational) PI who will sign off in CLARA (the UAMS IRB's e-submission system).
10.1.5, Key Roles and Study Governance	• Changed name and associated contact info for DCOC Overall cIRB PI:<br>FROM: Jessica Snowden, MD<br>TO: Songthip Ounpraseuth, PhD<br>• Added new DCOC Co-I's<br>○ Sherry Courtney, MD<br>○ Fred Prior, PhD<br>• Updated contact information for Medical Monitor	Sherry Courtney is an MD who will fill the MD part of Dr. Snowden's role. Dr. Prior has been with ISPCTN DCOC but has a new role within DCOC.

Summary of Key Changes from Version 04 to Version 05:

Affected Section(s)	Summary of Revisions Made from V-04 to V-05	Rationale
Cover page	Added NCT number	NCT number was not previously available.

Affected Section(s)	Summary of Revisions Made from V-04 to V-05	Rationale
Table 1.3.2	Updated well-child care visit points	The timing and number of well-child care visits are recommendations and cannot be specifically controlled by the study protocol or the study team.

FROM

1.3.2 PARTICIPANT LEVEL ACTIVITIES FOR CHILD-CAREGIVER DYADS

Study Activity	Week 1	Week 4–8* (± 2 weeks)	Week 12–16* (± 2 weeks)	Week 20–28* (± 2 weeks)	Week 32–40* (± 2 weeks)	Week 48 (± 2 weeks)
	Baseline Assessment	WCC 1	WCC 2	WCC 3	WCC 4	Post-Intervention Assessment
Consent	•					
Screening/Eligibility	•					
Demographics	•					
LENA	•					•
WCC Visit*		•	•	•	•	

* WCC visits will occur per the routine AAP Periodicity Schedule (i.e., WCC visits at 2, 4, 6, 9, 12, and 15 months), and the timing of WCC visits will vary by the child's age at enrollment. Enrollment occurs at 2-6 months (+ 0-30 days) of age and will also vary if child is on a "catch-up schedule" for missed WCC appointments.

Abbreviations. LENA = Language Environment Analysis, WCC = Well-Child Care, AAP = American Academy of Pediatrics.

TO:

1.3.2 PARTICIPANT LEVEL ACTIVITIES FOR CHILD-CAREGIVER DYADS

Study Activity	Week 1	Week 4–8* (± 2 weeks)	Week 12–16* (± 2 weeks)	Week 20–28* (± 2 weeks)	Week 32–40* (± 2 weeks)	Week 48 (± 2 weeks)
	Baseline Assessment	4 WCCs*				~~Post-Intervention Assessment~~
Consent	•					
Screening/Eligibility	•					
Demographics	•					
LENA	•					•
WCC Visit*						

* It will be recommended that the children have 4 WCCs between weeks 2 and 42. These WCC visits will – ideally - occur per the routine AAP Periodicity Schedule. However, the timing of WCC visits will vary because the enrolling sites do not have influence or control over whether the WCCs occur per the AAP guidelines. The enrolling sites will complete periodic chart reviews and record the dates of any WCC visits that occurred.

Abbreviations. LENA = Language Environment Analysis, WCC = Well-Child Care, AAP = American Academy of Pediatrics.

Affected Section(s)	Summary of Revisions Made from V-04 to V-05	Rationale
10.1.10; Protocol Deviations	Updated link to ICH E6(R2)	Simple correction.

Summary of Key Changes from Version 05 to Version 06:

Affected Section(s)	Summary of Revisions Made from V-05 to V-06	Rationale
1.3.1, Study Level Activities, table footer	Changed as shown in row of merged cells below:	Corrected for timing of clinician training.
FROM<br>*The initial recruitment window will be 4 months, prior to TWMB training in clinics, with an additional 3 months used if needed to enroll participants who have not had a previous WCC visit during the TWMB trial, for a total of 7 months of planned recruitment.<br>†Each clinic will complete TWMB training and run-in, on a rolling basis, when the initial 4-month dyad (parent/guardian + child) participant enrollment window is complete in their clinic.<br>TO<br>*The initial recruitment window will be 4 months. TWMB clinician training can occur during or after this 4-month recruitment window. ~~, prior to TWMB training in clinics, with a~~Sites will use an additional 3 months of recruitment ~~used~~ if needed to enroll participants who have not had a previous WCC visit during the TWMB trial, for a total of 7 months of planned recruitment.<br>†Each clinic will complete TWMB training and run-in, on a rolling basis, when the initial 4-month dyad (parent/guardian + child) participant enrollment window is complete in their clinic.		
1.3.2 Participant Level activities for Child-caregiver dyads (table footer)	See changes in merged-cells row below this row.	Corrections. Requirement to have participants complete questionnaires about LENA use was included in MOP but was not included in the protocol. Flexibility for timing for week 1 was added.
FROM:<br>* It will be recommended that the children have 4 WCCs between weeks 2 and 42. These WCC visits will – ideally occur per the routine AAP Periodicity Schedule. However, the timing of WCC visits will vary because the enrolling sites do not have influence or control over whether the WCCs occur per the AAP guidelines. The enrolling sites will complete periodic chart reviews and record the dates of any WCC visits that occurred.<br>TO:<br>* The study team ~~It~~ will ~~be~~ recommend~~ed~~ that the children have 4 WCCs between weeks 2 and 42. These WCC visits will, ~~–~~ ideally, occur per the ~~routine~~ AAP ~~Periodicity Schedule~~guidelines. However, the timing of WCC visits will vary because the enrolling sites do not have influence or control over whether the WCCs occur per the AAP guidelines. The enrolling sites will complete ~~periodic~~ chart reviews and record the dates of ~~any~~all WCC visits ~~that occurred~~.<br>** Every effort will be made to complete the baseline assessment(s) during week 1, but there may be circumstances when this is not possible. The study team will discuss these cases as they arise. These will not be considered protocol deviations.<br>*** The LENA assessment includes (a) using questionnaire to assess parent/LAR's understanding of LENA use, (b) having parent/LAR record adult-child vocal interactions, (c) having parent/LAR answer questionnaires about child's location and who child was with during each of the recordings, & (d) providing feedback on LENA use.		
2.3.1 Known potential risks	Added:<br>Participants will be told of the risks of using personal unsecured electronic communications such as unencrypted personal emails and unencrypted text messages	Clarification to ensure participants are aware of risks of using unencrypted personal emails/text messaging.
5.5.1.2 and 5.5.1.3	Changed "honorarium" to "compensation."	Simple correction.

Affected Section(s)	Summary of Revisions Made from V-05 to V-06	Rationale
5.5.2.1, Caregiver-child identification, screening & enrollment	Added the opt-out letter could be provided via mail OR electronically.	IRB has approved local context for using electronic health records for send outs.
5.5.2.1, Caregiver-child identification, screening & enrollment; subsection "Recruitment/ Enrollment Timeline"	First paragraph of subsection, added<br><br>"TWMB clinician training can occur during or after this 4-month recruitment window."	Clarified/corrected clinician training window.
6.1.2, TWMB Training	First para. changed<br><br>**FROM**<br>For each clinic, TWMB training for providers and care teams will occur after the initial 4-month recruitment window, or after recruitment and baseline assessments are completed and the target sample is met for the clinic, whichever comes first.<br><br>**TO:**<br>For each clinic, TWMB training for providers and care teams will occur during or after this 4-month recruitment window, or after recruitment and baseline assessments are completed, whichever occurs first.	Simple correction related to timing.
7.3. Lost to follow-up	Added "(recording)" behind "post-intervention assessment"	Clarification
8.1.2. Caregiver Assessments and Procedures, subsection "Primary Endpoint:"	5th full paragraph.<br><br>Added "If the family opts for electronic communications, the family will get an electronic link and be able to complete the review and questionnaire electronically.	Clarification on use of electronic communications.
8.1.2. Caregiver Assessments and Procedures, subsection "Primary Endpoint:"	After 5th full paragraph<br><br>Added entire paragraph:<br>"Participants will be asked to complete …."	Information was missing from protocol.
10.1.1.2, Consent Procedures and Documentation,	Added the following to 1st sentence of 2nd paragraph<br><br>… (by mail or via electronic health record, such as MyChart).	To clarify the electronic communications can be used in addition to regular mail.

Affected Section(s)	Summary of Revisions Made from V-05 to V-06	Rationale
10.1.11, Publication and Data Sharing Policy	Changed "Jeanette Lee" TO "Songthip Ounpraseuth"	To match personnel change at DCOC. Cover page and 10.1.5 (Key Roles and Study Governance) changed previously.

Summary of Key Changes from Version 06 to Version 07:

Affected Section(s)	Summary of Revisions Made from V-06 to V-07	Rationale
Section 1.1 (Study Population);<br>Section 4.2.1 (Study Design for Primary Objective)<br>Section 5. (Study Population)<br>Section 5.1.2. (Caregiver-child participant inclusion criteria)<br>Section 5.5.2.1 (Caregiver-child Identification, Screening and Enrollment Procedures)<br>Section 8.1.2 (Enrollment sub-section)	Increased recruitment window range<br>FROM: 1 to 6 months (or 2 to 6 months) of age<br>TO: zero to 9 months (+ 0-30 days)	• To try to increase enrollment rate, which has been slower than expected.<br>• To correct to areas where the protocol was not previously corrected (i.e., protocol still indicated enrollment at 2 months of age, when the meaning was intervention to begin not earlier than 2 months of age)
Section 1.1. (Study intervention);	• Removed references to Table 3, which was removed from section 5. (See below).	Internal consistency.
Section 4.1	• Added "up to" with regard to enrolling 66 dyads total.<br>• Added last sentence, 2nd paragraph:<br>o *Although it would be ideal for the dyads to be evenly distributed (33 dyads per clinic), the portion of dyads/clinic will be adjusted as needed to get to – or near – the total enrollment goal.*	Clarification.
Section 4.2.1, Study Design for Primary Objective	Added last sentence to 1st paragraph to ensure it was clear intervention doesn't start until child is at least 2 months of age even if enrolled earlier.	Clarification/emphasis.

<table>
<tr><th>Affected Section(s)</th><th>Summary of Revisions Made from V-06 to V-07</th><th>Rationale</th></tr>
<tr><td>Section 5. Table 3.</td><td>Removed Table 3.
<table>
<tr><th colspan="7">Table 3. Study Visit Schedule Based on AAP Periodicity Schedule for WCC Visits</th></tr>
<tr><th>1st WCC Visit for Study</th><th colspan="6">AAP Periodicity Schedule (Month)</th></tr>
<tr><th></th><th>2</th><th>4</th><th>6</th><th>9</th><th>12</th><th>15</th></tr>
<tr><td>2-Month</td><td>•</td><td>•</td><td>•</td><td>•</td><td></td><td></td></tr>
<tr><td>4-Month</td><td></td><td>•</td><td>•</td><td>•</td><td>•</td><td></td></tr>
<tr><td>6-Month</td><td></td><td></td><td>•</td><td>•</td><td>•</td><td>•</td></tr>
</table></td><td>Change in enrollment window means table was no longer reflective of all the well-child care visits that may occur after enrollment.</td></tr>
<tr><td>Section 5 para. 2</td><td>Deleted references to Table 3 information.

Deleted the highlighted sentence:
Within each clinic, we will aim for an equal allocation ratio regarding age at recruitment (i.e., 11 dyads before the two-month visit; 11 dyads before the four-month visit; and 11 dyads before the six-month visit), but at minimum we will aim for eight dyads in each age band.</td><td>Internal consistency and to reflect larger enrollment window.</td></tr>
<tr><td>Section 5.4, Screen Failures</td><td>Changed rescreening criteria:
Changed “1”
• FROM Consent but do not meet inclusion or do meet exclusion criteria and are not entered in the study,
• TO: Consent but do not meet inclusion or do meet exclusion criteria and have not done the initial LENA recording,

Changed:
• In second paragraph changed first word, i.e., “caregivers,” to “dyads”

ADDED:
• Paragraph 3 (Begins with “Dyads who screen fail because….”)</td><td>Increased ability to rescreen in order to increase enrollment rate, which has been slower than expected.</td></tr>
<tr><td>Section 5.5.2, Caregiver-child Recruitment and Retention</td><td>Added 2nd paragraph</td><td>Clarification/emphasis that 33 dyads/clinic is the ideal - but is not the required - distribution.</td></tr>
<tr><td>Throughout</td><td>Minor spelling/grammar corrections.</td><td>Simple corrections.</td></tr>
</table>

Summary of Key Changes from Version 07 to Version 08:

Affected Section(s)	Summary of Revisions Made from V-07 to V-08	Rationale
Thoughout	Updated total number of dyads FROM 66 TO 86. This means the total participant population is being increased FROM 132 TO 172.	The number of dyads who were above the 75th percentile for the 1st set of LENA recordings has been higher than expected. This means there would not be enough dyads entering the phase 2 part of the study to do a proper statistical analysis. The goal is to have at least 66 dyads entering into the second phase of the study (i.e., to be eligible for the 2nd LENA recording).
Throughout	Changed TWMB intervention (i.e., ages for which the intervention was given at the WCC visits) FROM children who are "< 36 months" of age TO children who are "4 to 36 months of age"	To increase recruitment windows by keeping children ages 0 to 3 months(+30) days naïve to the TWMB intervention.
Throughout	Increased the number of medical professionals – at each clinic - from whom patients can be enrolled. Changed FROM "n=2" to "up to 4" (per clinic)	To increase recruitment potential
4.2.1 Study design for primary objective, 1st sentence of para. 5	Changed<br>FROM: *To ensure dyads are naïve to the TWMB intervention, we will enroll our sample of dyads prior to completing the TWMB training for clinic providers.*<br>TO: *To ensure dyads are naïve to the TWMB intervention, we will enroll the majority of our sample of dyads prior to completing TWMB training for clinic providers; after training has been completed, we will only enroll dyads who are TWMB naïve due to age and/or not having a previous WCC visit with TWMB.*	Changed wording to be consistent with change in TWMB WCC intervention beginning at 4 months instead $\leq$ 36 months).
8.1.2, Caregiver Assessments and Procedures, subsection "Primary Endpoint: Caregiver Language-promotions Behaviors, para. 7	Expanded delivery services that may be used to return LENA devices.	Clarified that UPS, FedEx and other services can be used in addition to the US postal service (USPS).
10.1.5, Key Roles and Governance	Change role of Sherry Courtney, MD<br>FROM: Co-I TO: Medical Monitor<br><br>Removal of Rebecca Latch, MD, as medical monitor.	Change in personnel due to change in grant to a no-cost extension.

Affected Section(s)	Summary of Revisions Made from V-07 to V-08	Rationale
Throughout	Minor corrections to wording/grammar.	Corrections made for clarity and to match key changes listed in previous rows of this table.

Table of Contents

Statement of Compliance 16
1 Protocol Summary 17
1.1 Synopsis 17
1.2 Schema 19
1.3 Schedule of Activities 20
1.3.1 Study Level Activities 20
1.3.2 Participant Level Activities for Child-CAREGIVER Dyads 21
2 Introduction 22
2.1 Study Rationale 22
2.2 Background 23
2.2.1 Preliminary Steps 26
2.2.2 Conceptual Approach for Language-promotion Intervention 28
2.3 Risk/Benefit Assessment 29
2.3.1 Known Potential Risks 29
2.3.2 Known Potential Benefits 29
2.3.3 Assessment of Potential Risks and Benefits 30
3 Objectives and Endpoints 31
4 Study Design 32
4.1 Overall Design 32
4.1.1 Primary Objective and Hypothesis 32
4.2 Scientific Rationale for Study Design 32
4.2.1 Study Design for Primary Objective 32
4.3 End of Study Definition 34
5 Study Population 35
5.1 Inclusion Criteria 35
5.1.1 Clinic Inclusion Criteria 35
5.1.2 Caregiver-Child Participant Inclusion Criteria 36
5.2 Exclusion Criteria 36
5.3 Lifestyle Considerations 37
5.4 Screen Failures 37
5.5 Strategies for Recruitment and Retention 38

5.5.1 Clinic Recruitment and Retention 38
5.5.2 Caregiver-Child Recruitment and Retention 38
6 Study Intervention 44
6.1 TWMB Intervention Administration 44
6.1.1 TWMB Intervention Description 44
6.1.2 TWMB Training 49
6.1.3 TWMB Run-In 49
6.1.4 Study Staff Training 50
6.2 Preparation/Handling/Storage/Accountability 50
6.2.1 Acquisition and Accountability 50
6.2.2 Appearance, Packaging, and Labeling 50
6.3 Measures to Minimize Bias: Randomization and Blinding 51
6.4 Study Intervention Compliance 51
7 Study Intervention Discontinuation and Participant Discontinuation/Withdrawal 52
7.1 Discontinuation of Study Intervention 52
7.2 Participant Discontinuation/Withdrawal from the Study 52
7.2.1 Provider Replacement 52
7.3 Lost to Follow-Up 52
8 Study Assessments and Procedures 54
8.1 Efficacy Assessments 54
8.1.1 Clinic Procedures 54
8.1.2 Caregiver Assessments and Procedures 56
8.2 Safety and Other Assessments 60
8.3 Adverse Events and Serious Adverse Events 60
8.3.1 Definition of Adverse Events (AE) 60
8.3.2 Definition of Serious Adverse Events (SAE) 60
8.3.3 Classification of an Adverse Event 61
8.3.4 Time Period and Frequency for Event Assessment and Follow -Up 62
8.3.5 Adverse Event Reporting 62
8.3.6 Serious Adverse Event Reporting 63
8.3.7 Reporting Events to Participants 63
8.4 Unanticipated Problems Involving Risks to Subjects or Others (UPIRTSO) 64

8.4.1 Definition of UPIRTSO 64
8.4.2 UPIRTSO Reporting 64
8.4.3 Reporting Unanticipated Problems to Participants 65
9 Statistical Considerations 66
9.1 Statistical Hypotheses 66
9.2 Sample Size Determination 66
9.3 Populations for Analyses 66
9.4 Statistical Analyses 66
9.4.1 General Approach 66
9.4.2 Analysis of the Primary Efficacy Endpoint 67
9.4.3 Safety Analyses 67
9.4.4 Baseline Descriptive Statistics 67
9.4.5 Planned Interim Analyses 67
9.4.6 Sub-Group Analyses 67
9.4.7 Tabulation of Individual Participant Data 67
9.4.8 Exploratory Analyses 67
10 Supporting Documentation and Operational Considerations 69
10.1 Regulatory, Ethical, and Study Oversight Considerations 69
10.1.1 Informed Consent Process 69
10.1.2 Study Discontinuation and Closure 71
10.1.3 Confidentiality and Privacy 72
10.1.4 Multi-site Communications (IRB-related) 73
10.1.4 Future Use of Stored Specimens and Data 73
10.1.5 Key Roles and Study Governance 73
10.1.6 Safety Oversight 75
10.1.7 Clinical Monitoring 75
10.1.8 Quality Assurance and Quality Control 75
10.1.9 Data Handling and Record Keeping 76
10.1.10 Protocol Deviations 77
10.1.11 Publication and Data Sharing Policy 78
10.1.12 Conflict of Interest Policy 78
10.2 Additional Considerations 78

10.3 Glossary 78

10.4 Abbreviations 79

11 References 81

STATEMENT OF COMPLIANCE

The trial will be carried out in accordance with International Council on Harmonisation Good Clinical Practice (ICH GCP, specifically ICH E6(R2)) and the following:

- United States (US) Code of Federal Regulations (CFR) applicable to clinical studies that are not regulated by the FDA, specifically, (45 CFR Part 46)

National Institutes of Health (NIH)-funded investigators and clinical trial site staff who are responsible for the conduct, management, or oversight of NIH-funded clinical trials have completed Human Subjects Protection and ICH GCP Training.

The protocol, informed consent form(s), recruitment materials, and all participant materials will be submitted to the Institutional Review Board (IRB) for review and approval. Approval of both the protocol and the consent form (including HIPAA authorization) must be obtained before any participant is enrolled. Any amendment or modification to the protocol will require review and approval by the IRB before the changes are implemented to the study. In addition, all changes to the consent form and HIPAA authorization will be IRB-approved. A determination will be made regarding whether a new consent needs to be obtained from participants who provided consent using a previously approved consent form.

1 PROTOCOL SUMMARY

1.1 SYNOPSIS

Title: Talk With Me Baby: Leveraging Well-Child Care to Enhance the Early Home Language Environment for Rural and Underserved Children

Study Description: This pilot study will attempt to demonstrate the preliminary efficacy of embedding universal language promotion (Talk With Me Baby, TWMB) in well-child care (WCC) visit for enhancing the home language environment (HLE). It is a multicenter trial that will take place in two primary care clinics in two ISPCTN states that serve *rural and underserved children and families*. It is designed to provide the foundation for a full-scale trial, which will evaluate the efficacy of TWMB for enhancing both the HLE and key early childhood outcomes (language and school readiness).

Objective: Primary Objective — Explore preliminary efficacy of TWMB for enhancing the HLE for at-risk families, specifically by examining whether the language-promotion behaviors of caregivers (see glossary for specific definition) improve from Baseline to Post-Intervention.

Endpoint: Primary Endpoint: Improvement in language-promotion behaviors (as measured by Language Environment Analysis [LENA] Conversational Turn Count and Adult Word Count percentile scores) from Baseline to Post-Intervention.

Study Population: Total participant population will be up to 172 people. Participants will be enrolled as dyads (up to 86) consisting of 1 caregiver and their child (aged 0-9 months [+ 0-30 days] at enrollment). The study team will include children younger than 1 month of age but will not begin data collection (LENA) until the child is 2 months of age. The study team will include children 9 months plus up to 30 days but will not include children who have turned 10 months of age. The child participant will need to be receiving WCC in the participating clinics.

Phase: N/A

Description of Sites/Facilities Enrolling Participants: The study will take place in 2 primary care clinics within ECHO ISPCTN states that provide WCC services to children (≤ 36 months old). Clinics will serve a population of families that are at least ≥40% rural **or** at least ≥40% on Medicaid or are uninsured.

Description of Study Intervention: Primary care providers in participating clinics will be trained to embed **Talk With Me Baby (TWMB)** within WCC visits for all children, ages 4 to 36 months, as part of routine anticipatory guidance. TWMB will be embedded through the following provider actions: (1) Modeling talking with the child; (2) Sharing education about the importance of talking with the child;

	(3) Encouraging them to practice talking with the child; and (4) Giving a "Language Nutrition Prescription" to add extra talk at home.<br><br>Within each clinic, providers will deliver TWMB during all standard of care WCC visits for children who are 4 to 36 months old during the 12-month intervention period, following the usual/routine American Academy of Pediatrics (AAP) Periodicity schedule (which recommends WCC visits at 2, 4, 6, 9, 12, 15, 18, 24, 30, and 36 months). All health provider teams in each clinic will receive TWMB training. However, for this feasibility study, we will only collect data on the subset of patients, plus the patients' caregivers, that were consented. The study team will approach the caregivers of patients who are seen by a few (1 to 4) selected providers at each of the 2 clinics.<br><br>Children in the dyads enrolled in the intervention will receive usual care (i.e., routine anticipatory guidance) + a brief language-promotion intervention (TWMB) in up to 4 consecutive WCC visits with their participating provider during their 12-month trial period. For this pilot study, in each clinic we will enroll approximately 43 caregiver dyads in the trial. Total enrollment for the study is up to 86 caregiver-child dyads (for a total of up to 172 participants).
**Study Duration:**	2 years (3 months study start-up; 4 months rolling recruitment; 12 months final participants' completion; 5 months analysis and closeout). See Section 1.3 for timeline details.
**Participant Duration:**	12 months (with up to 13-month window to complete the study, allowing for leeway in the timing of WCC visits).

1.2 SCHEMA

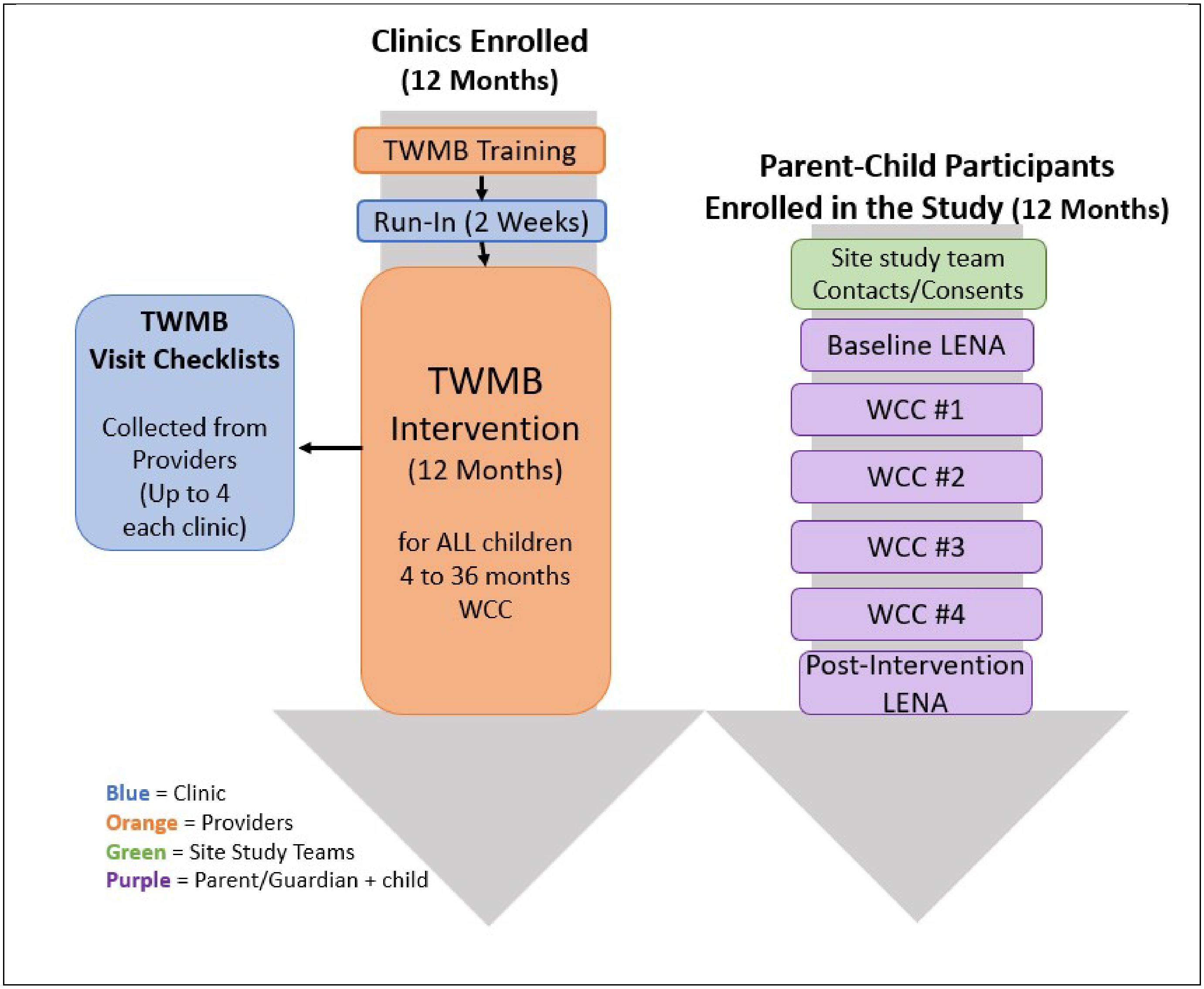

Abbreviations. TWMB = Talk With Me Baby; LENA = Language Environment Analysis; WCC = Well-Child Care; PI = Principal Investigator.
Note. LENA (Language Environment Analysis) “talk pedometer” recordings provide an automated measure of caregiver language-promotion behaviors (i.e., the home language environment).

1.3 SCHEDULE OF ACTIVITIES

1.3.1 STUDY LEVEL ACTIVITIES

Study Activity	Study Month																							
	1	2	3	4	5	6	7	8	9	10	11	12	13	14	15	16	17	18	19	20	21	22	23	24
Research staff training & prep	•	•	•																					
Preparation of Clinics	•	•	•																					
Send Weekly Patient Schedules to Study Team (for prescreening)				•	•	•	•	•	•	•	•	•	•	•	•	•	•	•	•					
Recruit/Consent Participants *				•	•	•	•	(•)	(•)	(•)														
Obtain Baseline Assessment/LENA				•	•	•	•																	
Process Participant Payments				•	•	•	•						•	•	•	•	•	•	•					
TWMB Training of Clinic Providers†						•	•																	
Clinic Run-In (2 weeks) †						•	•																	
TWMB Intervention (all 4 to 36 mo WCCs)						•	•	•	•	•	•	•	•	•	•	•	•	•						
Engagement Activities (Clinics & Dyads)						•	•	•	•	•	•	•	•	•	•	•	•	•						
Post-Intervention Assessment/LENA													•	•	•	•	•	•	•					
Study Closeout, Analyses, Dissemination																				•	•	•	•	•

Abbreviations. TWMB = Talk With Me Baby, WCC = Well-Child Care.

Note. Yellow = Protocol Study Team, Blue = Clinic, Green = Site Study Teams, Orange = Providers.

*The initial recruitment window will be 4 months. TWMB clinician training can occur during or after this 4-month recruitment window. Sites will use an additional 3 months of recruitment if needed to enroll participants who have not had a previous WCC visit during the TWMB trial, for a total of 7 months of planned recruitment.

†Each clinic will complete TWMB training and run-in, on a rolling basis, when the initial 4-month dyad (parent/guardian + child) participant enrollment window is complete in their clinic.

1.3.2 PARTICIPANT LEVEL ACTIVITIES FOR CHILD-CAREGIVER DYADS

**Study Activity**	**Week 1**	**Week 4–8* (± 2 weeks)**	**Week 12–16* (± 2 weeks)**	**Week 20–28* (± 2 weeks)**	**Week 32–40* (± 2 weeks)**	**Week 48 (± 2 weeks)**
	Baseline Assessment**	**4 WCCs***				Post-Intervention Assessment
Consent	•					
Screening/Eligibility	•					
Demographics	•					
LENA	•***					•***
WCC Visit*						

* The study team will recommend that the children have 4 WCCs between weeks 2 and 42. These WCC visits will, ideally, occur per the AAP guidelines. However, the timing of WCC visits will vary because the enrolling sites do not have influence or control over whether the WCCs occur per the AAP guidelines. The enrolling sites will complete chart reviews and record the dates of all WCC visits.
** Every effort will be made to complete the baseline assessment(s) during week 1, but there may be circumstances when this is not possible. The study team will discuss these cases as they arise. These will not be considered protocol deviations.
*** The LENA assessment includes (a) using questionnaire to assess parent/LAR's understanding of LENA use, (b) having parent/LAR record adult-child vocal interactions, (c) having parent/LAR answer questionnaires about child's location and who child was with during each of the recordings, & (d) providing feedback on LENA use.

Abbreviations. LENA = Language Environment Analysis, WCC = Well-Child Care, AAP = American Academy of Pediatrics.

2 INTRODUCTION

2.1 STUDY RATIONALE

The current pilot study aims to demonstrate the preliminary efficacy of embedding universal language promotion (TWMB) in WCC visits for enhancing the HLE. This study will act as a critical first step towards a rigorous, full-scale randomized controlled trial (RCT) on the efficacy of TWMB in WCC visits for enhancing both the HLE and key early outcomes (language acquisition and school readiness) for young children.

The HLE is a primary public health consideration because it can confer critical protection or risk for a young child; it is a stronger predictor of early neurodevelopment and long-term health, academic and economic outcomes, than parent income, education, race, and ethnicity.[1-11] Having an inadequate HLE—which is characterized by low quality and quantity of early language input from parents and caregivers—disproportionately affects children growing up in families of low-socioeconomic status (SES); among the 11 million infants and toddlers under age 3 years in the United States (US), 45% live in low-SES families.[12-14] Young children in rural and underserved areas (i.e., those with lower access to early childhood services, such as Head Start and early intervention, such as Part C services) are particularly at risk for disparities in the HLE. They are more likely than children in urban areas to experience higher and more persistent levels of poverty and reduced access to early childhood services.[15-22]

When parents and caregivers receive instruction on how to use language promotion, it benefits the quality and frequency of their language interactions with their child, which in turn improves child outcomes.[23-25] However, evidence-based interventions for language promotion have not been effectively deployed at scale to reach large proportions of disadvantaged families, with cost and access cited as the main barriers.[26-28] Primary care is an ideal solution to these problems. Since 91-98% of families with infants and toddlers access primary care services, they may be an ideal vehicle for delivering low-cost, scalable intervention to these parents and caregivers.[29]

Still, primary care clinics face practical challenges with providing language-promotion interventions. The AAP recommends universal developmental surveillance and screening at specific intervals (detailed in Bright Futures Guidelines),[30-33] but there is a lack of efficient, evidence-based tools to help healthcare providers deliver language promotion within routine anticipatory guidance. TWMB addresses this gap by allowing healthcare providers to embed evidence-based language promotion seamlessly within WCC visits.[34,35] TWMB uses evidence-based language-promotion strategies and has been deployed clinically for 8+ years,[35,36] but it has not yet been rigorously tested in an RCT for efficacy.

Our preliminary work conducted in primary care clinics in academic medical centers demonstrates the acceptability and feasibility of TWMB in urban, well-resourced settings.[37] However, to ensure the feasibility and address potential barriers within a full-scale, multicenter trial,[38,39] we need to evaluate the feasibility and preliminary efficacy of TWMB in lower-resourced rural and underserved primary care settings.

The goal of TWMB directly addresses the focus area of child neurodevelopment within the Environmental influences on Child Health Outcomes (ECHO) IDeA States Pediatric Clinical Trials Network (ISPCTN). TWMB aims to optimize the developmental potential of all young children, and specifically those at risk in rural and underserved areas, by boosting early language development and reducing long-term sociodemographic

disparities in health, academic, and economic outcomes. The ECHO ISPCTN is well suited for this evaluation because of the capacity within ISPCTN sites to scale TWMB to rural and underserved clinics throughout the US.[40] Evaluating TWMB across multiple ISPCTN states with rural and underserved families of diverse backgrounds will demonstrate the potential of early language promotion to benefit disadvantaged children and reduce intergenerational cycles of health inequities.[20,41,42] Further, demonstrating the efficacy of TWMB as a low-cost, scalable, and universal language-promotion approach has significant potential to change the standard of WCC.

2.2 BACKGROUND

Critical importance of the early language environment. Children's HLE—the quality and quantity of early language input from parents and caregivers—predicts their language development, literacy, and academic success.[1,2,8,43-49] It is the single strongest predictor of a child's vocabulary development by three years of age,[8] and early language-rich interactions with a caregiver support critical brain development necessary for a child's optimal language and long-term outcomes.[1,2,8,48] Neural networks for language acquisition are present before birth, and language input influences the brain's neural circuitry long before infants speak their first words.[50,51] Peak growth in synapses responsible for language occurs at six months and is influenced by a child's language environment.[52,53] Children who experience more language-rich interactions with parents and caregivers display more robust early neural connectivity and activation in specific brain regions that support language and literacy.[11,54-56] Notably, it is not overheard speech (i.e., simply hearing words from adults, television, or video), but rather speech directed to a child that facilitates their language development.[8,57] The effects of the HLE also become magnified over time: a child's vocabulary at three years of age predicts later academic achievement,[3-6,58] which in turn predicts their long-term health and economic outcomes.[3-6]

Effects of poverty on early language. Although there are many adverse child outcomes associated with poverty, language development is particularly vulnerable.[2,59] While all parents communicate with their children, a robust literature has established that children in low-SES families are less likely to receive optimal early language interactions with parents and caregivers. Specifically, they are likely to

- Hear fewer words,
- Hear less variety of words,
- Hear more prohibitive or negative language, and
- Take part in fewer conversational turns (i.e., an adult utterance, followed by a child utterance, or vice versa).[2,9,48,59-62]

SES differences in parent knowledge about language development also contribute to disparities in the HLE.[63,64] The effects of SES-linked differences in language input are evident on developing language as young as nine months, with differences in language processing and vocabulary evident at 18 months, and a six-month gap in skills between lower- vs higher-SES children by 24 months.[65,66] By three years of age, children in lower-SES families have heard 30 million fewer words, and have half the vocabulary on average, compared to children from higher-SES families.[2,60] Importantly, the number of conversational turns is a key indicator of the quality of the HLE and the strongest predictor of a child's later language skills.[11,67] As many as 65% of preschoolers from families with low-SES in Head Start have clinically significant language delays.[68]

This early language gap builds lifelong disadvantages in *health* (increased risk of engaging in unhealthy behaviors, increased risk to experience chronic disease, and reduced life expectancy), *academic* (increased odds of inability to read or to achieve third grade literacy, failure to graduate high school, and lower educational attainment), and *economic* (increased odds of lower income and unemployment) outcomes.[3-6] The lack of words and conversations in a young child's life has even been identified as a public health concern by the US Administration of Children and Families[14,52,69,70] due to the impact on neurodevelopment and long-term outcomes. This has resulted in the formation of the Health Resources and Services (HRSA)-funded Bridging the Word Gap Research Network.[71]

Evidence the HLE is malleable. The last three decades have yielded clear *evidence-based language-promotion strategies* that can optimize early parent-child language interactions.[24,25] These key language-promotion strategies specifically include:

- *Parentese*: high-pitched voice, elongated vowels, speaking with musical prosody.
- *Serve and Return/Conversational Turns*: noticing and responding to a child's communication signals (such as eye contact, sounds, and gestures) and expanding the number of back-and-forth communication exchanges.
- *Narration/Talking More*: increasing the amount and quality of language directed to child.[24,25,72-76]

Meta-analyses of these strategies in RCTs have also yielded evidence of strong, positive effects on parents' language-promotion behaviors and children's receptive and expressive language outcomes.[24,25] Specifically, studies indicate:

1. Educating parents about why talking with their child is important increases parent-child language interactions,[23]
2. Parents can be taught specific strategies to help them engage in more daily language interactions with their child,[24,25] and
3. These strategies can also be used effectively with parents and caregivers with low-SES.[72-75]

However, existing evidence-based interventions in home-visiting and childcare settings have not had the reach needed to narrow population-level language disparities.[26-28] The primary barrier has been finding ways to scale these interventions to large populations of parents and caregivers, especially underserved families who need this information most.[27,28] A possible solution is to use the primary care setting, as it offers several advantages: population-level access, high frequency of opportunities to reach children from birth to school entry, potential for low cost by using existing infrastructure and staff, and an opportunity to build on existing relationships between families and primary care providers within the patient-centered medical home.[77]

Rationale for targeting rural and underserved communities. All children who experience a disadvantaged HLE are at-risk for negative, long-term academic, health, and economic outcomes (see above). However, young children living in rural areas disproportionately experience multiple risk factors:

- Higher rates of poverty (24% for children under age five years of age in rural areas, compared to 17% in metro areas).
- Higher levels of poverty (of the 138 US counties with child poverty rates of 40% or higher, only 11 were metro counties).

- More persistent poverty over the last several decades (of 708 counties with persistent poverty, 80% were rural).
- Reduced access to key health and early childhood services.[15-22,78]

More specifically, children in rural and underserved communities experience: financial and geographical barriers to early childhood education programs (i.e., Early Head Start/Head Start Programs; Parents as Teachers), financial and geographical barriers to early intervention services (i.e., Part C services, developmental specialists, and speech language pathologists), shortages of health and childhood service providers, and fragmentation among available service providers.[17-19,22,78]

As such, rural and underserved children are more likely to experience a disadvantaged HLE. However, the early HLE can confer risk <u>or</u> protection for children in low-SES families.[7-10] Thus, enriching the HLE of rural and underserved children can substantially mitigate the adverse effects of poverty beginning at birth.[11,79,80]

The status of population-based efforts to intervene in the HLE. Efforts to enrich the HLE have taken two broad forms:

1. Literacy-focused interventions, and
2. Routine- or play-focused interventions.

In the primary care setting, the literacy focused intervention Reach Out and Read (ROR)[81] has been scaled with positive benefits for home reading frequency.[82,83] Notably, ROR is a low-intensity and low-dose intervention within WCC visits that yields significant change in parent behaviors related to reading at home. ROR yields promising results for enhancing child language and reaches 25% of low-income children. [81,84,85] However, low-income children that participated in ROR still demonstrate significant deficits in language and school readiness.[85-87] Thus, while this literacy-focused intervention targets an important context (i.e., book reading), this context only offers occasional opportunities to promote language.

A routine-focused intervention – the Video Interaction Project (VIP)[88] – has also been used in WCC with positive benefits for parent-child interactions and school readiness.[88] However, VIP has not been scaled because the time-intensive model (i.e., 30 min session at each WCC visit) and the need for dedicated staff for video-recording and coaching are not feasible for all primary care settings, especially rural and under-resourced clinics. <u>Still, both approaches point to the promise of primary care and WCC as vehicles for comprehensive language promotion among a significant percentage of caregivers</u>.[28,89]

Overall in the US, between 91–98% of children four years of age or younger have attended WCC visits within the past 12 months; the percentage is 91% for children living in rural communities and 95% for children with Medicaid insurance.[29] As such, WCC is an unmatched context for reaching young children and their caregivers to enrich HLEs.

TWMB as a primary care solution. We know that increasing opportunities for language interaction result in more positive language outcomes, and we can boost these outcomes by increasing the dose of language input parents provide to their child. One method in the primary care setting is to have healthcare providers encourage parents to make every activity with their child (e.g., diaper changes, bathing, playing, outings) a language-enriching occasion. However, standard of care anticipatory guidance does not give primary care providers any practical strategies for enriching caregiver-child language interactions to optimize the HLE.[28,90]

In response to this need, TWMB was developed in a statewide initiative in Georgia to embed "language nutrition" within WCC starting at birth.[34] The term language nutrition refers to the use of language that is sufficiently rich in engagement, quality, quantity, and context to nourish a child neurologically, socially, and linguistically. Similar to ROR,[91] TWMB builds on the idea that primary care providers have a unique opportunity to enrich the early HLE in families that are facing multiple challenges of poverty. It leverages trusted healthcare provider relationships and routine touchpoints (i.e., up to eight WCC visits by 15 months) to deliver evidence-based language promotion within the standard WCC time frame.[37] It also creates a systematic structure within existing anticipatory guidance and uses practical tools to:

1. Teach caregivers about the importance of early language interactions, and
2. Equip caregivers with specific, evidence-based language-promotion skills (i.e., using parentese, serve and return, narration) to increase language interactions with their child during their daily activities.

TWMB is well aligned with the anticipatory guidance objectives of the AAP's Bright Futures guidelines,[32] which identifies the importance of spoken language, screening, and education to promote language development in young children.

TWMB is also culturally appropriate for all families, as it emphasizes the importance of caregivers using their primary home language(s) with their child, and incorporates a family's individual routines and preferences, which can differ based on cultural background.

Additionally, TWMB has strong potential to complement existing literacy promotion within a clinic (like ROR). Because it focuses on language interactions before reading age, TWMB can be initiated at earlier, critical stages of child development and can be integrated into other parent-child routines and activities that occur throughout the day.

Importantly, while TWMB is built on evidence-based strategies, its efficacy has not yet been rigorously evaluated in a full-scale RCT.

2.2.1 PRELIMINARY STEPS

Given the high need, TWMB has been widely deployed across the state of Georgia in all 159 counties and 18 public health districts.[35,36] In the last four years, TWMB has been taught to 3,000+ health providers (physicians; nurses; allied health professionals; nutritionists; staff of Special Supplemental Nutrition for Women, Infants, and Children [WIC]) in hospitals, primary care clinics, obstetrics and gynecology (OB/GYN) clinics, neonatal intensive care units (NICUs), and other urban and suburban public health settings.[92] The majority of healthcare providers voluntarily participated in TWMB training and identified the value for their professional practice.

TWMB-trained healthcare providers demonstrated:

1. Significant knowledge gains about how to promote early language development, and
2. Increased self-confidence in their ability to provide language promotion to parents.[35,36]

These educational and clinically focused efforts demonstrate the high buy-in of clinical partners to incorporate evidence-based language-promotion practices within their routine clinical practice.

Table 1. Kansas City TWMB Preliminary Trial	
**Nurse Perspectives (*n* = 10) on TWMB after 9-month implementation trial**	**% Strongly Agree**
TWMB is feasible to deliver during WCC.	100%
I am confident in my ability to deliver TWMB.	100%
TWMB is important for child development.	100%
TWMB is important for my professional practice.	100%
I would recommend TWMB to my colleagues.	100%
**Caregiver Perspectives (*n* = 65) on TWMB after 9-month implementation trial**	**% Strongly Agree**
I understand the importance of talking to my child.	98%
Learning about language nutrition was helpful.	98%
Practicing language nutrition at WCC was helpful.	96%
I was confident using language nutrition with my child.	93%
I would recommend TWMB to other families.	96%
Abbreviations. TWMB = Talk With Me Baby, WCC = Well-Child Care	

Kansas City TWMB Preliminary Trial. Our group has completed preliminary steps aimed at the evaluation of TWMB efficacy. First, we conducted a pilot study in a large, at-risk, urban clinic serving diverse (40% African American, 27% Hispanic; 13.7% Spanish speaking), low-SES (78% on Medicaid) children and families in Kansas City, Kansas. Unanimous stakeholder feedback (*n* = 25) from physicians, nurses, staff, and administrators indicated that they perceived TWMB would be feasible to deliver and useful for families. Provider and care team surveys also indicated perceived time to complete TWMB during WCC visits was two to three minutes for most families and two to five minutes for higher needs families. Data from clinic records indicated actual time for WCC and TWMB was an average of two minutes longer than WCC as usual.

Second, TWMB was implemented by nurses (*n* = 10) during a nine-month trial with acceptable adherence to key TWMB components (75%) and was unanimously endorsed as acceptable and feasible to deliver during WCC visits (see **Table 1**).[37,93] Regarding parent behavior change, we found preliminary evidence that TWMB resulted in significant improvement in self-reported language behaviors of parents (*n* = 65) after three WCC visits.[37] We also identified barriers, facilitators, and strategies for embedding TWMB within existing primary care workflows, including development of specific intervention tools.

Third, we evaluated the reliability of our TWMB adherence measurement, the TWMB Checklist, which is a clinical intervention tool completed by providers during the WCC visit (see Section 6.1.1.2). Specifically, we compared provider's completion of the TWMB Checklist (i.e., self-report) to audio-recordings (*n* = 70*)* of WCC visits (ranging from 4-36-month-old WCC visits). Results indicated strong concordance (96% agreement) between what providers reported completing and what trained observers coded as completed from the audio recordings. Importantly, this self-completed checklist approach has been used successfully in previous studies (including within the ISPCTN network) and is considered a cost-effective and valuable strategy for providing information about the portability of behavioral interventions.[94]

Although these steps provide strong preliminary support for the feasibility of embedding TWMB in primary care, the TWMB Kansas City Preliminary Trial was held in a single, large, urban medical center, leaving many unanswered questions about the feasibility of implementation in smaller and/or rural medical clinics, which have fewer resources, fewer providers, and less infrastructure to support implementation. In addition, the preliminary trial did not include gold-standard measures of key outcomes that will be implemented in a large-scale RCT on efficacy. Specifically, objective measures of the HLE (i.e., LENA[95] "talk pedometer" recordings of caregiver-child language interactions) were not performed.

TWMB Provider Advisory Board. To address implementation of TWMB in small, rural, primary care clinics, we formed a Provider Advisory Board (PAB) - which included rural pediatricians, family medicine physicians, and nurses—to give input on the methods proposed herein. This group universally felt that TWMB was valuable and

could be readily integrated into clinical practice (see **Table 2**). Clinicians recognized that having a more formal structure of delivering language promotion would establish a more systematic approach to meet the language education goals for parents from the AAP's Bright Futures guidelines.[32] The PAB suggested that TWMB leverages a provider's routine WCC activity, including child-directed interaction and anticipatory guidance or usual educational approaches, to add practical, concrete strategies, intentional modeling, and caregiver practice. The PAB recognized TWMB creates opportunities for clinicians to:

1. Observe caregiver-child relationships (by inviting caregivers to talk with their child),
2. Conduct developmental surveillance and psychosocial and behavioral assessment,
3. Evaluate for adverse child experiences, and
4. Provide ways to increase positive caregiver-child interactions.

The PAB concluded such an initiative can be integrated into existing WCC processes and would likely be delivered by the physician in rural clinics. However, it also found the approach to be flexible enough to be delivered by other members of the health care team.

**Table 2. TWMB Provider Advisory Board Findings (*n* = 5)**
**Major Themes Identified from Meeting**
1. TWMB fits with routine workflow for WCC and anticipatory guidance (e.g., "can deliver with minimal time").
2. TWMB aligns with cost-effective care delivery (e.g., "high-quality, low-cost care"; "easy intervention that could have profound outcomes later in life").
3. TWMB adds value to clinical encounters (e.g., "gives better exam", "helps parent relax or child calm").
4. Physician recommended implementor in 1-provider clinics; feasible for provider and their care team delivery also.
5. TWMB process is similar for Family Medicine & Pediatrics.
6. TWMB is feasible to deliver and aligns with literacy promotion practices (i.e., ROR) in WCC.
Abbreviations. TWMB = Talk With Me Baby, ROR = Reach Out and Read, WCC = Well-Child Care.

In summary, several decades of research support the specific, evidence-based language strategies used in TWMB, but the benefits of delivery within primary care have not been rigorously evaluated. Clinical and educational efforts in Georgia and our pilot work in Kansas have demonstrated the promise of TWMB at the provider level. However, feasibility of TWMB at the clinic- and caregiver-levels in rural and underserved areas have not been tested, which is the critical next step before the first full-scale evaluation of TWMB efficacy.

2.2.2 CONCEPTUAL APPROACH FOR LANGUAGE-PROMOTION INTERVENTION

The TWMB approach follows the Ecobehavioral Model of Language Development, which identifies caregiver-child interactions as the causal mechanism for language development and provides a framework for language intervention evaluations.[96] The overall goal is to intervene early to enhance the quality and quantity of caregiver-child language interactions by targeting caregiver knowledge and language behaviors from birth. This enables the HLE to serve as a protective factor to benefit early-childhood neurodevelopment and language and long-term academic, health, and economic outcomes.

To enhance caregiver language behaviors, our approach follows existing, evidence-based language interventions by using a combination of brief education, modeling, and coaching.[25,97] Because this intervention targets caregiver language behaviors across all daily routines (e.g., bathing, mealtime, play, outings, shared reading), the

dosage of a caregiver's increased language input extends throughout a child's day to optimize the home language environment.[98] Delivering TWMB at WCC touchpoints during the 1st year of life (i.e., up to 5 WCC visits between 2- and 12-months of age) is in line with the dosage of evidence-based language-promotion interventions.[99] This dosage is also directly comparable to the delivery methods used for ROR, which is associated with significant changes in caregiver behavior.[82,83] Furthermore, our preliminary data from implementing TWMB in an academic medical center support meaningful gains in parent language behaviors.[37,93]

TWMB is a low-cost, sustainable approach designed to reach families by leveraging the workforce that regularly engages with the largest percentage of caregivers and children. Most evidence-based language-promotion interventions for vulnerable, low-income families have involved high-cost programs that are challenging to sustain or scale to large numbers of families. The primary care system is a largely untapped vehicle for reaching caregivers in low-income households with language-promotion intervention. TWMB is designed to be implemented by health providers of all disciplines and experience levels and has high dissemination potential at a population level. The low-cost, targeted approach of TWMB also has strong potential to inform translational practice for the health provider workforce serving young children and families.

2.3 RISK/BENEFIT ASSESSMENT

2.3.1 KNOWN POTENTIAL RISKS

This study poses minimal risk to participants. Participants will provide protected health information (PHI) or PHI identifiers (e.g., names, dates of birth) to the study (see DSMP for details). The study team will make every effort to store the electronic information in secure databases and the physical documents in secure facilities. However, there remains a risk of accidental and mandated disclosure of PHI or identifiers. If participant parents/LAR choose to use electronic communications with the study team members, the parent/LAR will be told of the risks of using personal unsecured electronic communications such as unencrypted personal emails and unencrypted text messages.

If any concerns about their child's development arise, participants will be referred to their/their child's primary care provider for any concerns about child development.

2.3.2 KNOWN POTENTIAL BENEFITS

The primary potential benefit of trial participation for health providers includes education about how to support caregiver-child language interactions that optimize the home language environment.

The primary potential benefit of trial participation for caregivers includes education about how to support their child's language development, which may lead to improved child outcomes. There could also be improvement in caregiver and child relationship/interactions. However, research has not documented these additional benefits.

There is also potential for benefit to future health providers, caregivers, and children if the study demonstrates that the intervention provides an effective means of educating caregivers about increasing language promotion.

2.3.3 ASSESSMENT OF POTENTIAL RISKS AND BENEFITS

The trial has minimal possible risks, and we will make every effort to minimize those described in Section 2.3.1 (see information from lena.org). The potential benefits of the proposed language-promotion intervention outweigh the potential risks.

Standards of medical care provided to the child participants will not be altered based on participants' study-related activities.

3 OBJECTIVES AND ENDPOINTS

OBJECTIVES	ENDPOINTS	JUSTIFICATION
Primary		
**Primary Objective:**<br><br>Explore preliminary efficacy of TWMB for enhancing the HLE for at-risk families, specifically by examining whether the language-promotion behaviors of caregivers improve from Baseline to Post-Intervention.	**Primary Endpoint:**<br><br>Improvement in language-promotion behaviors (as measured by LENA Conversational Turn Count and Adult Word Count percentile scores) from Baseline to Post-Intervention.	Embedding language promotion within WCC visits is intended to optimize caregiver-child language interactions and child outcomes. Obtaining data on changes in caregiver language behaviors offers preliminary evidence for TWMB efficacy.

4 STUDY DESIGN

4.1 OVERALL DESIGN

This multi-site trial will be conducted within two primary care clinics in rural and underserved areas in ECHO ISPCTN states. In each clinic, selected providers and their care teams will be trained to deliver TWMB intervention.

The study team will enroll up to 86 dyads receiving WCC with participating providers. The dyads will receive the TWMB intervention during a 12-month period. Although it would be ideal for the dyads to be evenly distributed (approx. 43 dyads per clinic), the portion of dyads/clinic will be adjusted as needed to get to – or near – the total enrollment goal.

The study team will assess changes in the HLE (i.e., caregiver's language-promotion behaviors) at Baseline (Week 1) and Post-Assessment (Week 48) using LENA "talk pedometer" audio recordings – the research standard for measuring talk with children[95] – as described Section 8.1.2 (see safety and privacy information on lena.org).

The study team has conducted preliminary steps to examine the feasibility of TWMB in urban, higher-resourced clinics in an academic medical center, but have not evaluated TWMB in lower-resourced and rural clinics. Efficacy of TWMB for caregiver language-promotion behaviors and child outcomes has not yet been rigorously evaluated. In preparation for a future full-scale RCT of TWMB efficacy, the current pilot will obtain an estimate of TWMB preliminary efficacy for improving caregiver language behaviors after intervention during WCC visits (Primary Objective). The hypotheses for this pilot were developed based on the theory of change and guided by language development and behavior change literature.

4.1.1 PRIMARY OBJECTIVE AND HYPOTHESIS

PRIMARY OBJECTIVE

Primary Objective: Explore preliminary efficacy of TWMB for enhancing the HLE for at-risk families, specifically by examining whether the language-promotion behaviors of caregivers improve from Baseline to Post-Intervention.

Primary Endpoint: Improvement in language-promotion behaviors (as measured by LENA Conversational Turn Count and Adult Word Count percentile scores) from Baseline to Post-Intervention.

Hypothesis: Among caregivers completing TWMB, at least 70% will demonstrate improvement in language-promotion behaviors from Baseline (Week 1) to Post-Intervention (Week 48).

4.2 SCIENTIFIC RATIONALE FOR STUDY DESIGN

4.2.1 STUDY DESIGN FOR PRIMARY OBJECTIVE

The study design is guided by our primary research aim, which is to obtain preliminary efficacy data for TWMB.

TWMB will be used at every WCC visit by participating providers and their care teams during the study intervention period with all children who are between 4 and 36 months of age, regardless of whether the family is enrolled in the study. This is because TWMB is an intervention designed to improve clinical care for all children within primary care well-child visits. We included TWMB delivery at WCC visits for children ages 4 to 36 months because this is the target age group for the TWMB intervention, although for feasibility purposes in this small pilot we will only enroll children aged zero to nine months (+30 days) old. (Note the LENA intervention, i.e., obtaining the 1st set of recordings, will not be started until the child is at least 2 months of age.)

We aim to conduct this pilot trial in two clinics in ECHO ISPCTN states to ensure the TWMB process can be implemented consistently outside of higher-resourced primary care settings. Clinic criteria are broadly inclusive to maximize the range of participating practices and enhance the generalizability of results (see Section 5.1).

For feasibility purposes, the study team will collect study measures from patients of up to 4 providers ("participating providers" hereafter) in each clinic. Clinics with multiple providers will be eligible to participate (and all clinic providers and their care teams will receive TWMB training), but the study team will limit data collection to patients (and the patient's parent/guardian) of the selected providers in each clinic (i.e., the study team will only collect data from patients of the selected subset (up to 4 per clinic) of providers during the trial). Because TWMB is designed to be delivered by pediatric health care providers regardless of training background (e.g., pediatrician or family medicine physician; physician assistant; nurse practitioner), all provider types are eligible to receive TWMB training. The study team will include a run-in period (i.e., pre-trial period lasting two weeks when providers will deliver TWMB during all WCC visits for children 4 to 36 months of age) prior to the intervention period to ensure TWMB can be implemented consistently and with acceptable adherence (see Section 6 for details). While there is no single standard for acceptable fidelity for behavioral interventions, positive effects are observed at adherence levels ranging from 60–80%.[94,100-102] In this pilot study, the study team will apply a 75% criterion for acceptable adherence to delivery of critical TWMB elements during a WCC visit, which is also in line with our previous data.[37]

The study team will enroll a sample of child-caregiver dyads in each clinic whose child members are between age zero and nine months (+0 to 30 days) old. We include this age range for the child participants (rather than children between 4 and 36 months of age) to minimize variability due to development within the sample. The study team will enroll child-caregiver dyads where the child is a patient of a participating provider at each clinic. Additionally, the existing initiative for patient-centered medical homes (empanelment of patients to specific providers or care teams) will help to ensure that participating children will continue to follow up with the same trained TWMB primary providers for the majority of their WCC visits. Existing primary care empanelment will help to decrease some of the provider-to-provider variability in delivering TWMB by ensuring that that child is following up with the same provider as frequently as possible during the study period. In clinics where the patient-centered medical home is not fully operational, study staff will work closely with clinic staff to maximize follow up in subsequent WCC visits with the same provider to ensure continuity. In addition, the benefits of patient empanelment will be underscored during provider and care team TWMB training sessions.

To ensure dyads are naïve to the TWMB intervention, we will enroll the majority of our sample of dyads prior to completing TWMB training for clinic providers; after training has been completed, we will only enroll dyads who are TWMB naïve due to age and/or not having a previous WCC visit with TWMB. This approach is common in behavioral intervention trials and important in situations where the intervention training may change the

standard-of-care practices (i.e., in this case, it could change the anticipatory guidance providers give to families related to language milestones and language development prior to enrolled dyads completing their baseline LENA assessment).

The pre-post intervention design allows us to evaluate participant completion of critical measures that will be used in the future, large-scale RCT. Importantly, it also allows us to estimate expected change in caregiver language-promotion skills in response to the intervention. Since TWMB targets caregiver language behaviors, the design includes a pre-post intervention assessment of the HLE using LENA audio-recordings, which is an objective assessment and the research standard for measuring talk with children.[95] These recordings will come from a small recorder ("talk pedometer") children wear (in a shirt pocket) for a day at a time. See Section 8.1.2 for details about LENA assessment.

In line with emerging guidance on pilot studies prior to high-quality large-scale efficacy trials, the current study design does not include a control group.[38,39,103] This is due to the high likelihood of an underpowered sample in this pilot to detect meaningful differences in the endpoints that will be evaluated in the large-scale RCT. By focusing the pilot on delivering TWMB in both clinics, we can maximize the number of dyads (n = 86, with goal of having at least 66 dyads able to enter phase II) to obtain a better estimate of variability in intervention response, rather than using resources to conduct underpowered difference tests between conditions for an untested intervention in this setting.

Site study teams will consent dyads and will complete baseline measures <u>prior to</u> the start of TWMB implementation in clinics (or prior to having a WCC visit with TWMB during the intervention period). To gather preliminary evidence that TWMB is effective for families with an inadequate HLE and to avoid ceiling effects in this small sample, we will enroll dyads with baseline LENA scores that are $\leq 75^{th}$ percentile compared to age-referenced normative data. Of note, previous data have shown that <u>without</u> intervention, caregiver's language-promotion behaviors do not show significant change across birth to 48 months of age. [104] Therefore, evidence of change in caregiver's language-promotion behaviors over the course of the TWMB pilot trial would be meaningful preliminary evidence of TWMB efficacy for the home language environment.

Note that in the future RCT, we will randomize participants at the clinic level to the TWMB intervention or care as usual control to evaluate TWMB efficacy for caregiver language-promotion behaviors, as well as child language outcomes. The large-scale efficacy trial will be adequately powered to include the continuum of home language environments and will not employ LENA cutoffs for enrollment. In the large-scale trial, it will also be possible to follow children over a longer period to allow evaluation of language outcomes across a wider age range.

4.3 END OF STUDY DEFINITION

The end of the study for dyads is defined as after the dyad completes the end of study assessment, or 13 months after they were consented, whichever comes first.

5 STUDY POPULATION

TWMB will take place in 2 clinics in ECHO ISPCTN states. Clinic criteria are broadly inclusive to increase the likelihood of success of a future RCT and enhance the generalizability of results. Within each clinic, we will have up to 4 providers or provider/care team members in this pilot study (for a maximum of 8 providers). Note that "provider" is used herein to refer to health providers and care team members (e.g., including physicians, physician assistants, nurse practitioners, nurses, and nursing assistants). In the case of a single-provider rural clinic, we will include the provider and at least 1 member of their care team. See Inclusion Criteria for Clinics, Section 5.1.1.

Within each clinic, we will recruit and enroll child-caregiver dyads whose child has an upcoming WCC appointment with a participating provider. We will recruit children aged zero to nine months (+ 0-30 days) between 0-30 days before their WCC visit, which will occur per provider's clinical discretion and routine AAP periodicity schedule and follow them up to four consecutive WCC visits during the 12 months of intervention or 13 months total - whichever comes first. This will maximize recruitment potential and capture a range of caregiver language behaviors with children of varying ages within the time constraints of the pilot study. We will recruit dyads with a child who receives primary care from a participating provider in this pilot study. Within each clinic, we will aim for an equal allocation ratio regarding age at recruitment. Furthermore, we will prioritize including families with lower SES by first contacting for participation those families whose children have Medicaid insurance.

This approach will also be employed in the future full-scale RCT, which will add randomization at the clinic level to either TWMB intervention or care as usual control.

5.1 INCLUSION CRITERIA

5.1.1 CLINIC INCLUSION CRITERIA

Participating clinics must meet all the following the inclusion criteria to be selected as clinic sites:

1. Provide primary pediatric general healthcare, including at least 300 unique WCC visits, from birth to 36 months of age during the last 12 months; this can include family medicine clinics.

   This WCC visit criterion was chosen to ensure an adequate pediatric population for study enrollment without excluding smaller rural clinics. Given that primary pediatric care in rural settings often occurs in family medicine clinics, including these clinics will increase the likelihood of success of a future full-scale trial and enhance the generalizability of results.

2. Have at least two full-time healthcare providers or provider plus care team member(s) (for example, physicians, physician assistants, nurse practitioners, nurses, nursing assistants, patient care technicians) who agree to participate in the study trial.

   Additionally, participating providers must be direct-hire employees of the clinic (i.e., not locum tenens providers), to ensure that there is consistent provider follow-up and to minimize participant drop out over the course of the study duration.

3. Have the capacity to generate patient lists and schedules (e.g., from electronic health records [EHR] or billing database structure) to identify potential participants within eligible ages.
4. Meet one or both of the following criteria for the patient population served:
    - ≥40% from a rural zip code as defined by rural-urban community area (RUCA) code ≥4.
    - ≥40% on Medicaid or uninsured.
5. Agree to have all providers who deliver WCC, and their care team members participate in TWMB training; this may include, for example, physicians, physician assistants, nurse practitioners, nurses, and nursing assistants.

    Providing training for all eligible clinicians offers a back-up if participating provider or care team member discontinues participation for any reason.

5.1.2 CAREGIVER-CHILD PARTICIPANT INCLUSION CRITERIA

For this study, each child-caregiver dyad will consist of:

1. A caregiver who will receive TWMB during routine WCC visits for their child at participating clinics, and
2. The child of the above caregiver for whom the WCC visits are conducted.

A potential caregiver participant must meet all the following inclusion criteria to enroll in the study:

1. Be the age of majority, or older, as defined by the state of residency.
2. Able to complete study measures in English.
3. Have the legal authority to consent to participate for themselves and to consent on behalf of their child.

To proceed to the follow-up portion of the study the caregiver (dyad) must have a LENA baseline assessment score that is ≤ 75th percentile compared to a child's age-referenced normative data.

A potential child participant must meet all the following inclusion criteria to be enrolled in the study:

1. Receive WCC at a participating clinic from a participating provider.
2. Be zero to nine months (+ 0–30 days) old at enrollment.
3. Was born at full term (> 37 weeks gestation).
4. Was born in a singleton birth (i.e., was the only child delivered during the birth).

5.2 EXCLUSION CRITERIA

A potential caregiver participant must not meet any of the following exclusion criteria to enroll in the study:

1. Has a cognitive impairment or a visual or hearing impairment known to the clinic that limits their ability to make decisions about participating or engaging with the assessments, based on the site primary investigator's (PI's) assessment and local human subjects' research policies.
2. Does not live with child or spend at least two full days (i.e., at least 48 hours) per week with the child.
3. Does not plan for the child to continue receiving services at the participating clinic for at least 12 months.

A potential child participant must <u>not</u> meet <u>any</u> of the following exclusion criteria to enroll in the study:

1. Significant developmental delay or cognitive, visual, or hearing impairment known to the clinic.
2. Previously attended WCC visit with TWMB prior to enrollment.

5.3 LIFESTYLE CONSIDERATIONS

Child-caregiver dyads will require access to transportation to primary care clinics to attend their routine WCC visits. If child-caregiver dyad participants miss WCC visits, they will not be excluded from the study, but attendance will be tracked by the study team.

The person accompanying an enrolled child to WCC visits during the intervention period may not always be the enrolled caregiver participant. At consent, the demographics questionnaire will identify primary caregivers involved in the child's care (including the typical amount of time spent with child).

5.4 SCREEN FAILURES

Screen failures are defined as child-caregiver dyads who either.

1. Consent but do not meet inclusion or do meet exclusion criteria and have not done the initial LENA recording, or
2. Meet inclusion criteria but do not consent.

Dyads who do not meet the criteria for participation in this trial (screen failure) because of meeting one or more exclusion criteria that are likely to change over time may be rescreened. Examples include a change in residence or returning to having the child receive primary care at a participating clinic.

Dyads who screen failed because one (or both) did not meet previous inclusion criteria (i.e., based on a previous version of protocol), can be re-screened/re-consented with the current version of the cIRB-approved protocol (and companion ICF) in effect at the time of the re-screening/re-consent.

Site coordinators must document and retain the following information for screen failures:

- Basic demography.
- Inclusion and exclusion criteria the participant did not meet.
- Other details as required.

5.5 STRATEGIES FOR RECRUITMENT AND RETENTION

5.5.1 CLINIC RECRUITMENT AND RETENTION

5.5.1.1 CLINIC RECRUITMENT PROCEDURES

The ECHO ISPCTN site awardee and research team has identified the primary care clinics selected for participation based on previous work. Clinics must meet the criteria defined above in Section 5.1.1.

The research team will conduct a structured assessment of potential clinics identified by ISPCTN sites during the selection process. This assessment will describe the following:

1. Demographics of patient population.
2. Access to clinic schedule of WCC visits and patient contact list.

Based on the obtained information, two clinics will be selected. If an enrolled clinic experiences unforeseen circumstances that impacts their ability to participate, a second clinic may be enrolled at the other site.

At the time of their enrollment, each clinic will provide the study team with a clinic schedule of WCC visits and patient contact list (see study-specific Manual of Operating Procedures [MOP] for details).

5.5.1.2 CLINIC COMPENSATION

Clinics will receive $1,000 compensation when enrollment begins. For participating in the study and returning TWMB Checklists each month, clinics will receive $1,000 at study closeout. The total possible compensation for each clinic is $2,000 ($1,000 [at beginning of enrollment] + $1,000 [closeout]).

5.5.1.3 PROVIDER COMPENSATION

Participating providers will receive compensation for their time. Each participating provider will be considered a TWMB Clinic Champion. They will receive $250 for completing TWMB training and any other required study training for. They will also receive $1,000 compensation for being the TWMB Clinic Champion during the trial.

5.5.1.4 CLINIC AND PROVIDER RETENTION AND ENGAGEMENT STRATEGIES

The research team will provide clinics and participating providers regular study updates, clinic-specific feedback on progress, and messaging about TWMB (e.g., tips for TWMB delivery).

Clinics and participating providers will have the opportunity for regular check-ins with the study team (i.e., at least monthly and on an as-needed or as-requested basis) to answer questions and provide support.

Support can be provided via email, phone, or video-based communication platforms, per the preference of the clinics and participating providers.

5.5.2 CAREGIVER-CHILD RECRUITMENT AND RETENTION

Site study team will conduct recruitment, screening, and enrollment activities. Within each clinic, study staff will aim to recruit approximately 43 child-caregiver dyads with the characteristics described above in Section 5.1.2.

Sites will have an initial four-month primary recruitment period to enroll child-caregiver dyads and complete baseline assessments, prior to the TWMB intervention in clinics. After the TWMB intervention begins, study staff can use an additional three months of recruitment, if needed, to enroll eligible caregiver-child participants that have not had a previous WCC visit with TWMB during the intervention period. Sites will have a total of seven months of planned recruitment.

Note that in the event that one of the study sites (ceding entities) has trouble recruiting approx. 43 dyads, the 2nd study site (ceding entity) will have the opportunity to increase the number of dyads at their site.

Based on sample size estimates and weekly minimum recruitment targets, the goal accrual is two dyads per week per clinic, for a study-wide goal accrual of four participants per week during the initial four-month recruitment window. The total target enrollment size across all clinics is 86 child-caregiver dyads (see Section 9.2 for sample size determination). Throughout the recruitment period, the study team will assess accrual at least twice monthly to decide whether it is necessary to adjust recruitment strategies.

If accrual rate is lower than expected, we could employee the following strategies:

- Have trusted clinic staff discuss the project with families.
- Update recruitment materials to address areas of concern for families related to recruitment.
- Follow patients from other TWMB-trained clinic providers.

Additional adjusted strategies that will be considered to achieve goal recruitment will include:

- Extend the primary recruitment window for sites beyond the initial seven-month period (an extension up to three months).
- Extend child age

5.5.2.1 CAREGIVER-CHILD IDENTIFICATION, SCREENING, AND ENROLLMENT PROCEDURES

The team will request a partial HIPAA waiver to allow study sites to pre-screen medical records for potential participants. Each clinic will provide the research team with a patient list for all children less than or equal to nine months (+ 0 to 30 days) of age. The list should include:

- Provider name.
- Patient name.
- Patient age.
- Parent/caregiver name.
- Phone number.
- Email address.
- Insurance provider (Medicaid status).
- Family language (if available in EHR).

Each clinic will also provide the research team with a clinic schedule of WCC visits for all patients ≤ 36 months of age (see information about the clinic schedule of WCC visits in Section 8.1.1). The schedule should include both upcoming appointments (for the next four weeks) and past appointments (for the past week):

- Provider name.
- Patient name.

- Patient age.
- Parent/caregiver name.
- Phone number.
- Email address.
- Date and time of appointment
- Insurance provider (Medicaid status).
- Family language(s) (if available in EHR).
- Status of past week's visits (attended, cancelled, or no show).

Before recruitment begins, all patients will be receiving (mail or electronic via the potential participant's electronic health record) an IRB-approved opt-out letter allowing for them to express their desire to opt out of sharing PHI for pre-recruitment screening purposes. Information about how to join the study will also be included. This model has been used successfully in two prior ISPCTN clinical trials. However, if the central Institutional Review Board (cIRB) at the University of Arkansas for Medical Sciences (UAMS) or the local IRB does not permit contacting families to opt out prior to recruitment, the opt-out-of-contact strategy will not be implemented.

Study staff will use the patient list and clinic schedule of WCC visits to identify and recruit potential participants as illustrated in **Figure 1**. Study staff will complete the following steps to identify caregivers to contact and to select the order of contact:

1. Identify potential participants who qualify based on inclusion and exclusion criteria available from the patient list and clinic schedule (see Sections 5.1.2 and 5.2).
2. Categorize the family in terms of child age and Medicaid or uninsured status.
3. Contact caregivers of children within the category of Medicaid or uninsured status first as a strategy to ensure diverse participant enrollment.

Flyers will be available in clinics (and can also be given to families by clinic staff) to increase awareness of the research study and the potential to be contacted by study staff, as well as to permit opt-out of being contacted for recruitment.

Alternatively, recruitment methods can be locally modified if other methods work better for the population.

Figure 1. Site Study Team Workflow for Clinic Schedule of WCC Visits and Participant Recruitment

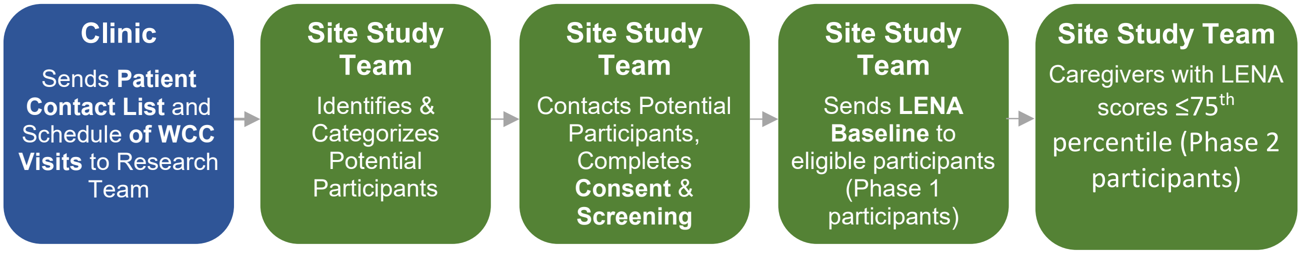

CONTACTING POTENTIAL PARTICIPANTS

If the caregiver and child meet all the inclusion and none of the exclusion criteria, the study staff (i.e., the site research teams) will contact the caregiver to describe the study. Study staff will contact caregivers by phone, email, or text (if allowed by local IRB). If caregivers are unreachable by any of these methods at first contact, study staff will follow up with at least two additional outreach attempts by one or more methods (phone, email, or text (if allowed by local IRB)).

SCREENING POTENTIAL PARTICIPANTS

For those interested in participating, study staff will begin the informed consent process with caregivers (see Section 10.1.1).

Prior to consent, site study staff will complete The Inclusion/Exclusion Form to confirm participants meet all inclusion and no exclusion criteria. See Sections 5.1.2 and 5.2.

As part of recruitment, study staff will use the clinic schedule of WCC visits to develop a Recruitment/Screening Log to record.

1. The number of potential participants they attempted to recruit,
2. The number they were able to successfully reach,
3. The number that subsequently consented,
4. The contact method(s) used, and
5. Reasons for not consenting if applicable.

These will be monitored overall and by site and used to inform adjustments to recruitment strategies for accrual.

See the study-specific MOP for detailed screening and recruitment processes.

ENROLLMENT OF CAREGIVER-CHILD PARTICIPANTS

After child-caregiver dyads are consented and screened into the study, the study team will complete LENA baseline assessment with the families, as shown in **Figure 1**. See consent procedures in Section 10.1.1 and LENA assessment procedures in Section 8.1.2.2.

To continue in the study to the second LENA assessment, the scores for child-caregiver dyads for the first LENA assessment must be ≤ 75th percentile, compared to age-referenced normative data.

Phase 1 child-caregiver dyads (i.e., those consented into the study but do not proceed to Phase 2) will still receive TWMB as part of their WCC visits with participating providers, as will all children in the clinic attending 4-month to 36-month WCC visits, but they will not complete the post-intervention assessment (i.e., the 2nd LENA recording). The research team will send dyads who cannot continue with the study due to high LENA scores a letter on behalf of their primary care provider that positively reinforces their high-quality HLE.

RECRUITMENT/ENROLLMENT TIMELINE

As described above, sites will have a total of 7 months of planned recruitment. The first 4 months will be the initial recruitment window. An additional 3 months will be added if needed to enroll participants who have not had a previous WCC visit during the TWMB trial. TWMB clinician training can occur during or after this 4-month recruitment window.

Note that the large-scale efficacy trial, which will utilize clinic-level randomization to TWMB intervention or care as usual control, will also employ this rolling recruitment strategy with child-caregiver enrollment and baseline assessment prior to a WCC visit during the trial phase.

5.5.2.2 CAREGIVER COMPENSATION

Child-caregiver dyads will receive compensation for their time. Each dyad will receive $30 when the site receives the signed informed consent form and demographics questionnaire. They will also receive $50 at Baseline and $50 at Post-Intervention for completing LENA recordings and returning (in a prepaid postage mailer or by dropping off at clinic) the LENA recorder and vest to the study team. The total possible compensation is $130 ($30 [consent, demographics] + $50 (LENA recording Baseline and LENA recorder returned) + $50 (LENA recording Post-Intervention and LENA recorders returned).

5.5.2.3 CAREGIVER-CHILD RETENTION AND ENGAGEMENT PLAN

To engage and retain at-risk families who may be hesitant to engage in research, we will use strategies that have been recommended in previous clinical trials with low-income racial and ethnic minority families from wide-ranging geographic areas and diverse settings (e.g., we will engage clinic providers as trusted community partners, engage study staff who are culturally sensitive, contact potential participants actively and repeatedly, and provide incentives for completing measures).[105] We will use IRB-approved contact methods/language, which may include phone or text message reminders and engagement touchpoints.[106] We will also utilize local Community Advisory Boards (CABs) to provide feedback on recruitment and retention materials.

To address barriers related to caregiver language and literacy levels, we will ensure study measures are at an accessible reading level and offer completion by interview.

Study team activities to maximize engagement will include the following:

- Meet with CAB(s) to ensure that recruitment materials are culturally appropriate as well as to gain feedback on messaging to maximize recruitment.
- Contact participants at enrollment with a thank you card for participating in the project.
- Employ multiple contact modalities for communicating with participants throughout the study, including (but not limited to) email, phone call, and text (if allowed by local IRB) messages.
- Send study measures electronically by email or text (if allowed by local IRB), with optional completion by phone or in-person interview with study staff.
- Send reminder prompts for study measures (email and/or text) if the initial prompt has not been completed.
- Follow up by phone if study measure still has not been completed.
- Send LENA “talk pedometer” recorders to caregivers by mail, along with LENA “how-to” video and written/visual instructions for use. Instructions will also be sent by email or text. Follow-up phone call to walk caregivers through using LENA, check for caregiver understanding of instructions, and answer any questions.
- Make every effort to maintain and regain contact with participants, including multiple attempts using the modalities listed above, as well as a certified letter to the participant’s last known mailing address or local equivalent methods.
- Contact participants with touch-point communication (i.e., email, text, phone) periodically during the trial to share information about the project (i.e., study milestones, etc.).
- Provide participants with link to the study website for background information about the study.

Detailed retention/engagement strategies will be included in the study-specific MOP.

These strategies will also be employed in the future full-scale trial.

6 STUDY INTERVENTION

6.1 TWMB INTERVENTION ADMINISTRATION

6.1.1 TWMB INTERVENTION DESCRIPTION

TWMB is designed to be embedded within routine WCC anticipatory guidance to reach families of infants and toddlers. It offers practical tools to support provider delivery of concrete strategies to foster an enriched HLE within anticipatory guidance (i.e., aligning with the AAP's Bright Futures[32] objectives) across multiple touch-points (i.e., up to 8 WCC visits by 15 months of age). TWMB is also designed to be appropriate for families of diverse cultural backgrounds because it emphasizes the importance of increasing caregiver-child language interactions within the family's existing routines and daily practices, as well as the importance of caregivers using their primary home language(s) with their child.

TWMB creates a clinic workflow structure for primary care providers and their care teams to deliver routine anticipatory guidance following AAP guideline that recommends anticipatory guidance related to language development at specific intervals (detailed in Bright Futures Guidelines)[30-33] during routine WCC for all families with children between 4 and 36 months of age.

TWMB will be used by participating providers at every WCC with all children ages 4 to 36 months of age during the intervention period, regardless of whether the family is enrolled in the study. In addition, within enrolled clinics all providers and their care teams will be trained to deliver TWMB prior to the start of the trial.

6.1.1.1 TWMB CLINIC WORKFLOW

TWMB is delivered within usual WCC workflows for anticipatory guidance during the clinical encounter (see **Figure 2**). It is delivered by the primary health provider (i.e., pediatrician or family medicine physician, physician assistant, nurse practitioner) and/or the provider and their care team.

Figure 2. Talk With Me Baby Clinic Workflow

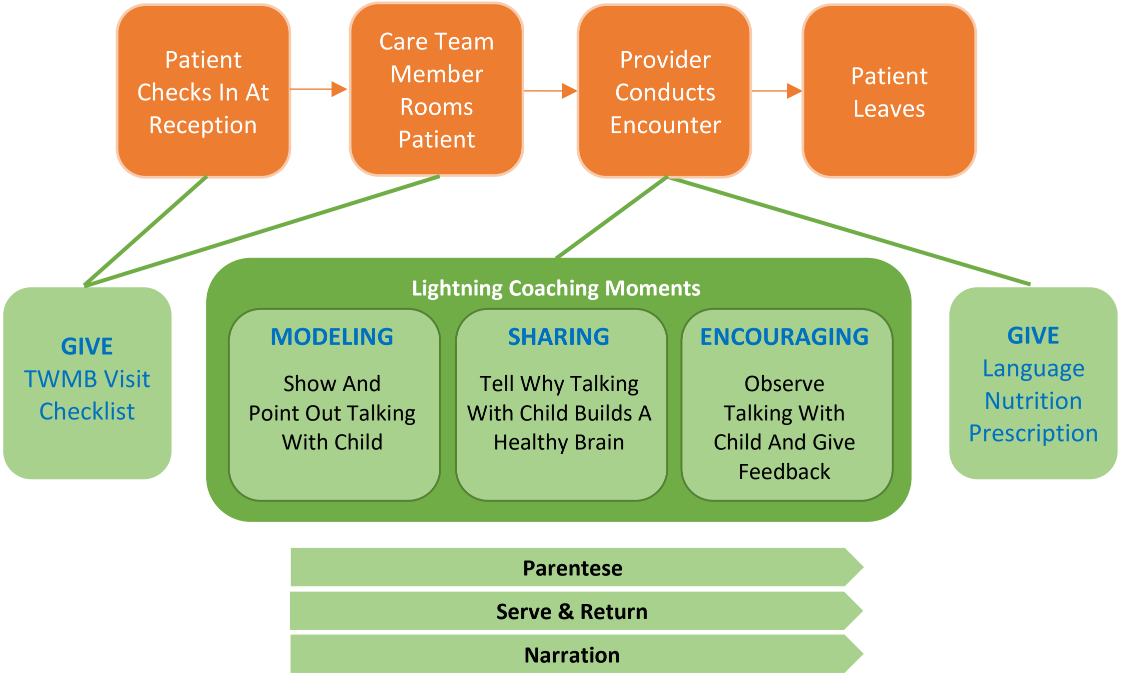

Providers use "lightning coaching moments" to apply an "I do – We do – You do" structure within the standard of care WCC time frame.[37] This combination of brief education, modeling, and coaching[25,97] is an evidence-based approach to enhance caregiver knowledge and language behaviors.

Because TWMB integrates within existing WCC activities there is minimal added time (i.e., average two to three minutes guidance, but less than one minute added time). The TWMB workflow has been reviewed by our PAB, who agreed this was readily achievable and of high value. This workflow was also implemented successfully in our preliminary work.

Specifically, the TWMB primary intervention components embedded in this workflow include:

**Modeling**	**Showing how** to talk with child, **pointing out** key language-promotion strategies and how child is responding.<br>*For example, the provider greets and talks directly to child (e.g., "Hi Lina, how are you today?"). The provider models using one or more key language skills throughout the visit (e.g., narration: "We are going to see how you are growing today. Let's look at your ears…Now I'm going to check your tummy…") and points out how the child is responding (e.g., "See how she is smiling and looking at me! She likes hearing about what is happening!").*

**Sharing**	**Telling why** talking build's a child's brain and why it is important for school readiness and future health. *For example, the provider shares education about how talking helps brain development (e.g., "You might notice I use a sing-song voice with lots of expression. We call that parentese and it's been shown to help children learn language much more easily.").*
**Encouraging**	Observing caregiver talking with child and providing feedback (**coaching**). *For example, the provider uses teach-back to encourage caregiver-child talking during the visit (e.g., "Dad, why don't you try parentese while you get Lina dressed. Don't worry if you feel silly, she loves hearing your voice!"). The provider gives feedback and highlights accomplishments (e.g., "That was great, your voice helped Lina calm and she watched you while you were talking to her using parentese!").*
**Language Nutrition Prescription**	**Giving a prescription** to increase home language interactions during daily routines. *For example, the provider asks the family about opportunities for talking (e.g., "Just like Lina needs food to grow, she also needs language nutrition for a healthy brain. You give Lina language nutrition every time you talk with her. It is so important we have a language nutrition prescription for talking to Lina. During which of these daily routines could your family use parentese with Lina?).*

Detailed intervention procedures, including use of the Language Nutrition Prescription, will be provided in the TWMB Intervention Manual.

6.1.1.2 TWMB KEY LANGUAGE-PROMOTION STRATEGIES

TWMB targets three key evidence-based language-promotion strategies (see **Figure 3**), because they are important predictors of increased brain development and later language *and* have been shown to be skills that can be taught to caregivers, including low-SES families, to improve their language interactions with their child.[72-75]

Figure 3. TWMB 3 Key Language Strategies

Parentese	Tune In & Take Turns	Talk More
**Parentese** is a speaking style with young children characterized by melodic tone, varied intonation; higher pitch; elongated consonants and vowels; short, simple and repeated; precise pronunciation and grammar.	**Serve and Return** (also known as *"*Tune In and Take Turns") is noticing what child is interested in and how a child is responding; following in to talk about what a child is interested in; taking turns and keeping the conversation (or back and forth sounds) going.	**Narration** (also known as "Talk More"*)* is talking about what is happening around child, about what child is doing, about what you and child are doing together (i.e., be a 'sportscaster'); turning daily routines and activities into opportunities for talking.

These three language strategies are the focus of TWMB lightning coaching moments: the provider focuses on one (or more) of these language strategies to model for, share with, and encourage caregivers to use during the WCC visit. The provider then gives a language nutrition prescription for the caregiver to use the strategy during routines at home with their child.

6.1.1.3 TWMB INTERVENTION TOOLS

TWMB delivery is facilitated by specific intervention tools—the TWMB Checklist and Language Nutrition Prescription—that will be used at every WCC for children ages 4 to 36 months. These tools facilitate TWMB delivery by serving as <u>cues to action for providers and their care teams</u> and <u>educational resources for caregivers</u>.

TWMB CHECKLIST

The TWMB Checklist is a brief checklist that providers and caregivers will use during the WCC visit. It contains the primary intervention components in a format that allows the provider and the caregiver to check off the topics discussed during the WCC visit (see **Figure 4**).

A TWMB Checklist will be available for each visit (i.e., available in each room or brought by the provider) and will serve as a cue to action to complete TWMB steps during the WCC visit. As the provider discusses each of the components of TWMB during the visit, the provider and the caregiver will check the boxes off to ensure that each aspect has been discussed. This allows for active participation of the caregiver as the provider goes through the tenets of TWMB. Additionally, it minimizes provider-to-provider variability and enhances standardization of the approach within the WCC visit. At the end of the visit, the TWMB Checklist goes home with the family as an educational handout and reminder to use language-promotion strategies.

Figure 4. TWMB Core Intervention Components and TWMB Checklist Components

TWMB Core Components	TWMB Checklist
Modeling Talking	❑ **Tips for Talking With Children**<br>(e.g., My provider showed me how to *Tune In & Take Turns* with my child.)
Sharing Education	❑ **Talking With a Child Is Important for Healthy Development.**<br>(e.g., I learned that *Talking with a Child Builds a Healthy Brain*.)
Encouraging Practice	❑ **Practicing Talking**<br>(e.g., *I practiced how to Tune In & Take Turns with my child*.)
Giving Language Nutrition Prescription	❑ **My Language Nutrition Prescription**<br>(e.g., *At home, we can add extra talking during diaper changes.*)

While self-reported checklists do expose the study to social-desirability bias, the action of having both the provider and the caregiver fill out the checklist together minimizes this risk. Each individual holds the other accountable for completion of the entire list as it actually occurred during the visit. Jointly completed checklists have been used successfully in primary care to support intervention delivery, and caregivers and providers have found this approach to be helpful.[107] In addition, similar visit checklists have been successfully collected from providers in rural primary care settings.[107] Our preliminary work also indicates high concordance between provider-completed TWMB Checklists and coder ratings from audio-recordings of WCC visits.

For research and data collection purposes, the TWMB Checklist will be used in a paper form. The TWMB Checklist will go home with the family as an educational handout (as usual), and a copy will be retained by the provider and returned to a central clinic location for collection by the study team. While traditional paper forms may be more feasible and preferable in lower-resource settings, the TWMB Checklist will also be available in electronic form, depending on clinic preference and access, as determined during training and run-in.

See Section 8.1.1.1 for procedures for TWMB Checklist data collection.

LANGUAGE NUTRITION PRESCRIPTION

The Language Nutrition Prescription is a cue to action for the provider to tell caregivers to:

1. Add more talking with their child at home ("Language Nutrition"), and
2. Identify home or daily routines when they can add language nutrition.

It is an educational tool that goes home with the caregiver and provides a concrete, actionable way for them to increase the frequency and quality of their daily language interactions with their child to enhance the HLE. The provider and caregiver jointly identify routine(s) during which the family would like to increase talking with their child (e.g., diaper changes, mealtime, bathing, playtime, etc.).

The Language Nutrition Prescription is designed to be tailored to the family's individual routines and preferences, as well as to the child's developmental level. Providers and families can select home routine opportunities from a list of choices or write in a more individualized opportunity.

Detailed intervention procedures, including use of the TWMB Checklist and Language Nutrition Prescription, will be provided in the TWMB Intervention Manual.

6.1.2 TWMB TRAINING

For each clinic, TWMB training for providers and care teams will occur during or after this 4-month recruitment window, or after recruitment and baseline assessments are completed, whichever occurs first.

TWMB training involves didactic and practice-based instruction that includes:

- A brief review of the science behind why talking with children matters.
- Focused instruction (with video examples and modeling) on how to deliver components of TWMB, including the TWMB Checklist and Language Nutrition Prescription during WCC; and
- Practice and role play with the trainer on how to deliver TWMB across child ages and different families and how to use the TWMB Checklist and Language Nutrition Prescription.

Dr. Brenda Salley will host mandatory training (approximately 2 hours) for all providers delivering WCC on the TWMB intervention at a participating clinic. She will provide instruction sufficient to ensure each provider demonstrates acceptable adherence to delivery of critical TWMB elements during WCC role play to complete training. Acceptable adherence will be defined as completion of 75% of TWMB Checklist components (See **Figure 4**). This criterion was set based on preliminary TWMB data[37] and is in line with the behavioral intervention literature demonstrating positive results at adherence levels ranging from 60–80%.[94,100,101] Training will be offered a minimum of two different times per clinic to accommodate providers and care teams. Clinic training will be considered complete when 100% of providers and care team members delivering WCC are trained (including part-time providers). All clinic providers and care teams will attend TWMB training. However, only selected providers (up to 4 per clinic) will be designated as participating “Clinic Champions.” Training all providers will also standardize the intervention dose by allowing a child-caregiver dyad to receive TWMB during a WCC that occurs with a different non-participating provider in the practice.

A follow-up training opportunity will be offered, after the initial training, to allow additional practice-based instruction to address challenges and barriers after providers have had the opportunity to use TWMB in their day-to-day clinical practice.

For detailed training procedures, see the study-specific MOP.

6.1.3 TWMB RUN-IN

After TWMB training, we will complete a two-week run-in period to ensure that participating providers are delivering TWMB during their WCC visits (at least 70% of sampled WCC visits for children between 4 and 36 months of age) and delivering critical TWMB elements during WCC visits (at least 75% of primary intervention components) prior to start of the intervention.

Provider adherence will be assessed via the TWMB Checklist (described in Section 6.1.1). For participating providers only, the site coordinator will review and log all TWMB Checklists collected, to compare to our target of 70% uptake. Clinics will complete a second training and run-in period if needed to achieve this criterion.

6.1.4 STUDY STAFF TRAINING

To support consistent and high-quality data submission in compliance with protocol and study operations, the DCOC and study team will train research staff who are listed on the sites' delegation logs. Study activities will be completed by centralized study staff and local site study staff. The DCOC expects all participating site study staff to take responsibility for learning the study, primarily by studying the protocol and MOP, to ensure compliance with the protocol and good clinical practice (GCP). Study staff will receive training through the following methods:

- A mandatory training session in the form of an Investigator Meeting.
- One-on-one site-specific training.
- Other targeted training as needed.

DCOC personnel will train all site study staff on

- The protocol.
- Potential study deviations and noncompliance.
- Reporting procedures for study deviations.
- Essential documentation for study conduct.
- In-person and remote consent informed consent processes and documentation.
- Identifying severe adverse events (AEs) and serious adverse events (SAEs) and how to report them.
- Any relevant electronic data capture (EDC) system(s) and study-specific data collection.

All study staff that are on a delegation log will also be required to complete Human Subjects (Research) protection (HSP) training per their local site's requirements and Good Clinical Practice (GCP) training per NIH requirements (https://grants.nih.gov/policy/clinical-trials/good-clinical-training.htm). Documentation of HSP and GCP training must be made available to the DCOC lead study team. Both HSP and GCP training must be kept current while a person is involved in study conduct (i.e., listed as active on the delegation log).

Dr. Brenda Salley will host mandatory training for study staff on the collection of LENA data (i.e., procedure for instructing families on how to use LENA and ensuring understanding; procedure for uploading LENA recordings to software system; procedure for locating LENA scores for enrollment purposes). See Section 8.1.2 for details on LENA assessment.

6.2 PREPARATION/HANDLING/STORAGE/ACCOUNTABILITY

6.2.1 ACQUISITION AND ACCOUNTABILITY

N/A

6.2.2 APPEARANCE, PACKAGING, AND LABELING

N/A

6.3 MEASURES TO MINIMIZE BIAS: RANDOMIZATION AND BLINDING

N/A

6.4 STUDY INTERVENTION COMPLIANCE

The study team will measure and monitor compliance and summarize compliance measures as follows:

- Clinic Compliance: TWMB Checklist usage by participating providers, defined as the percent of all eligible WCC visits (child 4 to 36 months of age) for which a TWMB Checklist was completed during the two-week run-in period.
- Caregiver Compliance: Child and caregiver participant attendance at WCC visits at which TWMB was delivered.

7 STUDY INTERVENTION DISCONTINUATION AND PARTICIPANT DISCONTINUATION/WITHDRAWAL

7.1 DISCONTINUATION OF STUDY INTERVENTION

See **Section 2.3.3** and **Section 4.3**.

7.2 PARTICIPANT DISCONTINUATION/WITHDRAWAL FROM THE STUDY

The study team will consider the following to be an early-terminated participant:

1. Any Phase 1 participant who does not proceed to Phase 2.
2. Any Phase 2 participant who later withdraws their consent, or
3. Any enrolled participant whom the site PI removes from the study.

The study will not replace clinics that withdraw early. If withdrawal occurs, the study will recruit additional providers, along with their patients, in the clinics that are already participating.

The study team will not replace early terminated caregiver participants.

Participants may voluntarily withdraw from the study at any time, or the site PI, the Data and Safety Monitoring Board (DSMB), and/or DCOC can withdraw a participant. Participants can be removed if they do not follow study instructions or it is not in the child's/caregiver's best interest to continue. If a participant voluntarily withdraws from the study, site coordinators or PIs can ask the participant why they chose to withdraw. Participants do not have to answer that question if they choose not to. Site coordinators will document the reasons, if available, for withdrawal.

7.2.1 PROVIDER REPLACEMENT

The study team will replace providers whose patients are enrolled in the study by engaging a new provider within the same clinic. New providers may complete booster training if needed to achieve acceptable adherence. Additionally, providing training at the clinic level for TWMB will ensure that there is a repository of trained providers who could quickly and effectively fill the role of a participating provider with little to no downtime.

7.3 LOST TO FOLLOW-UP

A participant will be considered lost to follow-up if

1. They do not the complete post-intervention assessment (recording), and
2. Site study staff are unable to contact the participant to complete the assessment as described below.

The following actions must be taken if a participant fails to complete a scheduled study assessment:

- The study team will attempt to contact the participant to complete the missed assessment and ascertain if the participant wishes to continue in the study.

- Before a participant is deemed lost to follow-up, the site coordinator or designee will make every effort to regain contact with the participant (where possible, emails, text messages, telephone calls, and if necessary, a certified letter to the participant's last known mailing address or local equivalent methods). These contact attempts will be documented in the participant's study file.
- The site PI will attempt to contact the participant by phone.
- Should the participant continue to be unreachable, they will be considered to have withdrawn from the study with a primary reason of lost to follow-up.

To minimize the number of participants lost to follow-up, the study team will send reminders via email and text (if allowable per local IRB policies) to non-respondents. Site coordinators will call remaining non-respondents. The study team will also utilize strategies used previously to engage and retain at-risk and underserved families (see Section 5.5.5.2.3 and strategies will be outlined in the study-specific MOP).

8 STUDY ASSESSMENTS AND PROCEDURES

8.1 EFFICACY ASSESSMENTS

For both clinic and caregiver procedures, activities may be completed by site study staff, as noted below, or determined by clinic and site preferences.

For the schedule of activities, see Section 1.3.

8.1.1 CLINIC PROCEDURES

CLINIC SCHEDULE OF WCC VISITS COLLECTION PROCEDURES

Each clinic will provide the study team with a weekly schedule of the previous weeks' visits and the upcoming weeks' visits for all patients between 4 and 36 months of age (i.e., the clinic schedule of WCC visits). We include children between 4 and 36 months of age because this is the target age group for the TWMB intervention. Participating clinics will provide the clinic schedule of WCC visits during the clinic preparation phase and throughout the trial.

The clinic schedule of WCC visits and how site study teams will use it is described in Section 5.5.2.1. The study team will use the clinic schedule of WCC visits to maintain a Recruitment/Screening Log (see Section 5.5.2.1). During the run-in, they will enter WCC visit data for enrolled child-caregiver dyads into the study database and log visit details for WCC visits with sampled TWMB Checklists to calculate rate of TWMB implementation for participating providers.

TWMB CHECKLIST COLLECTION PROCEDURES

The TWMB Checklist will be used to determine TWMB implementation by participating providers (i.e., proportion of their WCC visits with TWMB delivery) as part of TWMB Training and run-in period. For additional details about the TWMB Checklist intervention tool, see Section 6.1.1. Details will also be provided in the TWMB Intervention Manual.

In each clinic, the TWMB Checklist carbon copy will be collected from participating providers for all WCC visits for children 4 to 36-months of age. **Figure 5** illustrates the suggested workflow for TWMB Checklist collection for research purposes. Estimated time to complete the TWMB Checklist is less than one minute. Our PAB identified this process as a feasible approach. Jointly completed checklists have been used successfully in primary care to support intervention delivery, and caregivers and providers have found this approach to be helpful.[107] Furthermore, provider completed checklists have been used successfully to assess adherence and fidelity during primary care visits in rural clinics, and providers and families report this is a preferred approach.[107]

Figure 5. Suggested Workflow for TWMB Checklist Collection for Research

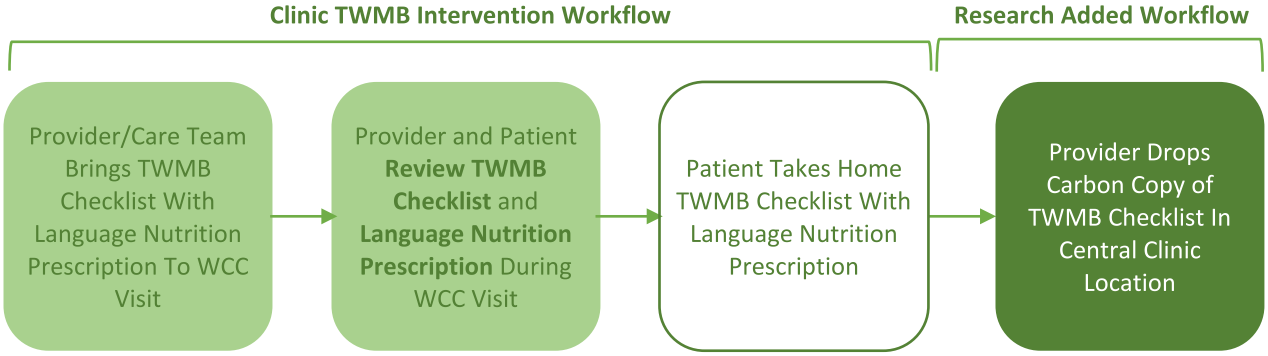

Each clinic is unique and has its own physical workflows, so implementation of the process in **Figure 5** will be tailored to each clinic to minimize loss of study materials. Systematic processes will be implemented to ensure that integration of TWMB Checklists (and collection of the checklists for research purposes) flow seamlessly within each clinic's existing workflow. System-based quality improvement measures to increase implementation and retention of study materials include creating a Visual Workplace with clear signage, collection areas that are within the normal physical workflow of movement of people and materials, and visually stimulating collection areas to ensure no physical study materials are discarded or lost.

Adaptations may need to be made based on feedback from clinic staff to ensure that the process integrates smoothly within their workflow. Proper integration into existing clinic workflow will ensure acceptance and buy-in by clinic staff through limiting waste and excessive movement.

The research team will collect all TWMB Checklists (provider carbon copy only) that participating providers complete during WCC visits during the intervention trial for each clinic. Note that the TWMB Checklist is an intervention tool, but the collection of the carbon copy allows access to clinical data for research purposes. Note that the TWMB Checklist will contain minimal data elements, specifically *date of visit*, *time of scheduled visit,* and *provider name* for purposes of linking it to the clinic schedule of WCC visits.

During the run-in, for participating providers only, the research team will review and log TWMB Checklists from WCC visits for all children who are 4 to 36 months of age. Missing TWMB Checklists will be logged as missing and considered not completed for the WCC visit for the purposes of training adherence.

Self-completed checklists as a measure of adherence to intervention is cost-effective, requires minimal time for data collection, and can provide important information about the portability of an intervention for dissemination.[94] Importantly, we have also established this approach as a reliable data collection method in our preliminary work – provider's self-report on the TWMB Checklist was highly reliable (96% agreement) with coded audio-recordings of WCC visits.

See **Figure 6** for definitions of TWMB implementation and acceptable adherence to delivery of critical TWMB elements during a WCC visit. The criterion for acceptable adherence was set based on preliminary TWMB data[37] and is in line with the behavioral intervention literature demonstrating positive results with adherence levels ranging from 60–80%.[94,100,101]

Figure 6. TWMB Implementation and TWMB Adherence Definitions

**TWMB Implementation**	=	*WCC Visits for 4 to36 months of age with acceptable TWMB adherence* / *Total WCC Visits for children 4 to 36 months of age*

Acceptable TWMB Adherence	*Completion of 75% of TWMB Checklist Components*	***Modeling*** *Talking*<br>***Sharing*** *Education*<br>***Encouraging*** *Practice*<br>***Giving*** *Language Nutrition Prescription*

8.1.2 CAREGIVER ASSESSMENTS AND PROCEDURES

ENROLLMENT

Enrollment will occur at infant age zero to nine months (+ 0-30 days) at Week 1. (The study team will include children less than 1 month of age but will not begin data collection (LENA) until the child is 2 months of age. The study team will include children 9 months plus up to 30 days but will not include children who have turned 10 (ten) months of age.) If caregivers are unreachable at first contact for recruitment/consent (see Section 10.1.1), the study team will follow up with additional outreach attempts (at least two additional attempts).

The site study team will complete screening surveys with caregiver participants. These surveys will ask for caregiver's contact information (name, phone number(s), physical address, email address, preferred contact method) and demographics (e.g., age and race/ethnicity). For the child participant, the child's parent/guardian will be asked to provide the child's name, race/ethnicity, primary care physician, primary caregiver(s) for child, and date of birth. These surveys will also ask about the availability of internet access and smartphones that participants could use for study-related communication.

Study staff will:

1. Send caregivers an electronic link (by email or text, depending on caregiver preference and what is allowed by local IRB) to complete consent and demographics questionnaires.
2. Complete the questionnaires with caregivers in person or by phone, or
3. Send by United States Postal Services or other standard document delivery services
4. Any combination of the above.

Research staff will follow up with reminders for completion (after initial invitation). These methods (which we have used successfully in previous large-scale longitudinal studies) are feasible given national data indicate 85% of adults have smartphones (77% have broadband).[110]

Other recruitment, screening, and enrollment procedures for caregiver-child participants are described in Section 5.1.2. As part of recruitment, the study team will maintain a Recruitment/Screening Log, as described in Section 5.5.2.1.

TRACKING WCC ATTENDANCE AND TWMB INTERVENTION DURING WCC

Child-caregiver dyads will receive TWMB during their WCC visits, as described in Section 6.1. The site study team will track their WCC attendance using the clinic schedule of WCC visits, as described in Section 5.5.2.1. This tracker will include scheduled appointments and attended appointments, as follows:

1. Identify and log child who attended a WCC during the past week.
2. Log visit details including the WCC provider.

PRIMARY ENDPOINT: CAREGIVER LANGUAGE-PROMOTION BEHAVIORS (LENA)

Caregivers will complete *LENA* recordings at Baseline (Week 1) and Post-Intervention (Week 48). LENA, the research standard for measuring talk with children, provides a quantitative measure of the HLE. LENA uses a small recorder ("talk pedometer") children wear in a vest pocket for a day at a time[95] (See **Figure 7**). It meets US and international safety standards, does not transmit, and has the same low-power processors as hearing aids. LENA software processes the audio into talk data (with built-in fidelity measures).

The LENA recording system, i.e., LENA SP, that will be used for this study allows – depending on the installation process - for recordings (".wav" audio files) to be downloaded to local computer systems. In order to prevent actual audio (.wav) files from being downloaded or retained for this study, the site's Information Technology (or equivalent) department will install the LENA Hub on the sites' computer(s) and the installation will be tested to ensure that files cannot be downloaded or retained. No one can listen to files that have not been downloaded or retained. The specifics for this set-up and testing will be provided in either the study-specific MOP or a specific document for LENA set-up and testing. LENA corporation email(s), which will provide additional information about the LENA SP system, are being submitted as a separate document for UAMS IRB review. In summary, the actual words spoken by the child and family will NOT be downloaded or kept by the site, the DCOC, or LENA corporation and no one will be able to listen to the audio files.

The sites will complete appropriate pre-enrollment testing of the LENA process with volunteers who will not be eligible to be TWMB study participants. This pre-enrollment testing will include (1) setting up the sites computers as noted above and completing that computer set-up testing, (2) sending LENA devices to at least 1 – but not more than 4 - volunteer families per site, (3) having the volunteer families complete the in-home LENA procedure as specified below in this section of the protocol, (4) having the LENA device sent back to corresponding site, (5) having the site process the recording to ensure the uploading and processing works as intended and the words cannot be accessed by anyone. No identifiable information will be kept about the volunteers and the information from the pre-enrollment testing will not be generalizable knowledge. If requested by the cIRB, a short informed consent will be provided for the volunteers so that they are fully

informed that their conversations will be recorded but that those recordings will not be used other than to ensure the process is ready for use and works as intended. They will be informed that neither LENA nor the study personnel will be able to listen to their actual words.

Figure 7. LENA "Talk Pedometer" Recorder and Vest With Instructions

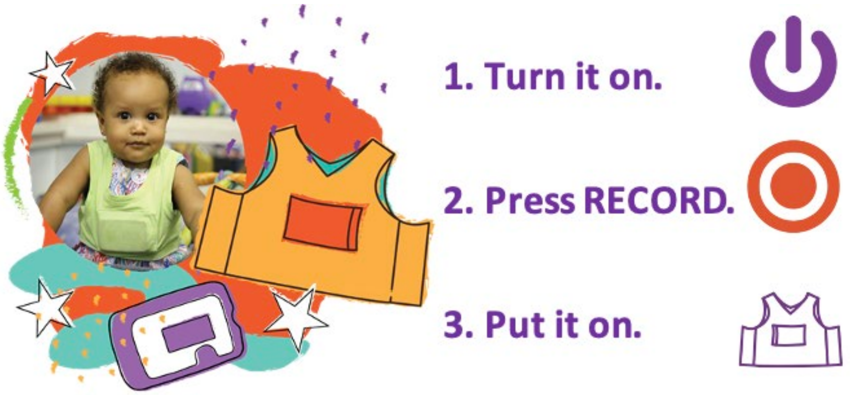

LENA is the research standard for measuring talk with children because it is user-friendly and widely used in the field to successfully collect data from families of diverse characteristics. Since the technology became available in 2008, LENA data have been published in 150+ observational and interventional studies involving many hundreds of children. LENA is family-friendly and has been used successfully in many large-scale studies with infants and toddlers using the methods we will use.[111]

Study staff will mail the LENA recorder and vest to the family; alternatively, the LENA recorder may be provided to the family at a clinic visit. Study staff will then complete a short phone call with the family to walk through how to use the device and answer any questions (simple instructions are also provided with the device, see **Figure 7**). If the family opts for electronic communications, the family will get an electronic link and be able to complete the review and questionnaire electronically. Families will be asked to complete 2 day-long recordings during a period when the child is in the home setting (i.e., not at daycare), turning on and putting on the device at the start of the day when the child first wakes up and leaving it on for 12-16 hours. The LENA recorder can remain turned on and be taken off and left nearby during bath or naps, if needed. The device automatically turns off when the maximum recording time is reached. Families will be asked where the recording was obtained. Families will complete a recording on two separate days during Baseline (i.e., Phase 1); Phase 2 participants will complete a recording on two separate days again during the Post-Intervention Assessment phase.

Participants will be asked to complete questionnaires at the end of each recording day. These questionnaires will ask about where the child was (based on 2-hour intervals) during the recording time period, what the child was doing, and who was with the child during the recording time period. A paper version of the question will be sent with the LENA device. The adult participant will have the option to answer the questions electronically, by phone, or by sending the completed paper questionnaires back with the LENA devices (see below).

After completing the Baseline recording, the family will mail (includes USPS, UPS, FedEx, and other reputable delivery services) the LENA recorder and vest back to the study team in a prepaid mailer or drop the device off

at the clinic. Phase 2 participants will complete the same process during the Post-Intervention Assessment phase. Completion of LENA recordings by mail has been done successfully in previous large trials.[112]

Site study staff will download the recording from the device to LENA's speech recognition software, which automatically analyzes the data into counts and percentiles for adult words and conversational turns. LENA captures clear adult words spoken near the child wearing the LENA recorder and is highly accurate in terms of agreement with human identification (82, 76, 71, and 76% for adult, child, television, and other speech, respectively).[113,114]

The LENA software provides a fidelity and quality indicator for the data recording. LENA software provides the following quality errors to indicate an unusable recording:

- Too much overlapping noise to detect audio sample (as determined by LENA software).
- Recording too short for processing (less than 5 hours of recording time).
- Not enough child or adult speech to process (as determined by LENA software).

If the received recording does not meet LENA fidelity benchmarks to extract a usable recording, the site study team will send the LENA device back to the family in a second attempt to collect an additional sample. The site study team will enter LENA standard scores at Baseline and Post-Intervention assessments in the study database (see **Figure 8** for example LENA output), which will be used for primary outcome data, including:

- Daily **Adult Word Count (AWC)** directed to the child.
- Daily **Conversational Turn Count (CTC)** between the adult and the child.

The LENA age-referenced normative data are based on the LENA Natural Language Study, which included a large and diverse sample of children 2 to 48 months of age and their caregivers.[104] AWC does not vary as a function of child age, so the normative data include a single mean and standard deviation for two to 48 months of age. CTC mean and standard deviation increase with age up to 25 months, then remain steady through 48 months, so the normative data include monthly intervals from two to 25 months of age.

<u>Complete LENA data will be defined as follows: at least one usable recording (i.e., usable recording from one day of the two possible recording days) is collected from caregiver participants within two attempts (to account for potential errors in recording) at Post-Intervention.</u>

We will monitor completeness/usability of LENA throughout baseline data collection to optimize our strategies for collecting follow-up data. Many studies using LENA have obtained 100% usable data at baseline, with other studies reporting a range of 70–100% usable data.[75,111,115-119] In our own lab, we have obtained 93% usable LENA data with underserved and low-SES populations. Usable data includes compliance with wearing the LENA vest and adequate length of recording time. We will ensure caregiver understanding of LENA recording procedures by sending a LENA "how-to" video and written/visual instructions to caregivers and by completing a follow-up phone call to walk caregivers through using LENA, check for caregiver understanding of instructions, and answer any questions (see detailed procedures in the study-specific MOP). We will track reasons for unusable data.

If data usability obtained in TWMB at Baseline drops below 70%, we will conduct a root-cause analysis and present a plan for correcting the problem to the DSMB. If the source of unusable data could not be corrected, we will re-evaluate our plan to collect LENA at Post-intervention.

Figure 8. LENA Data Report Example

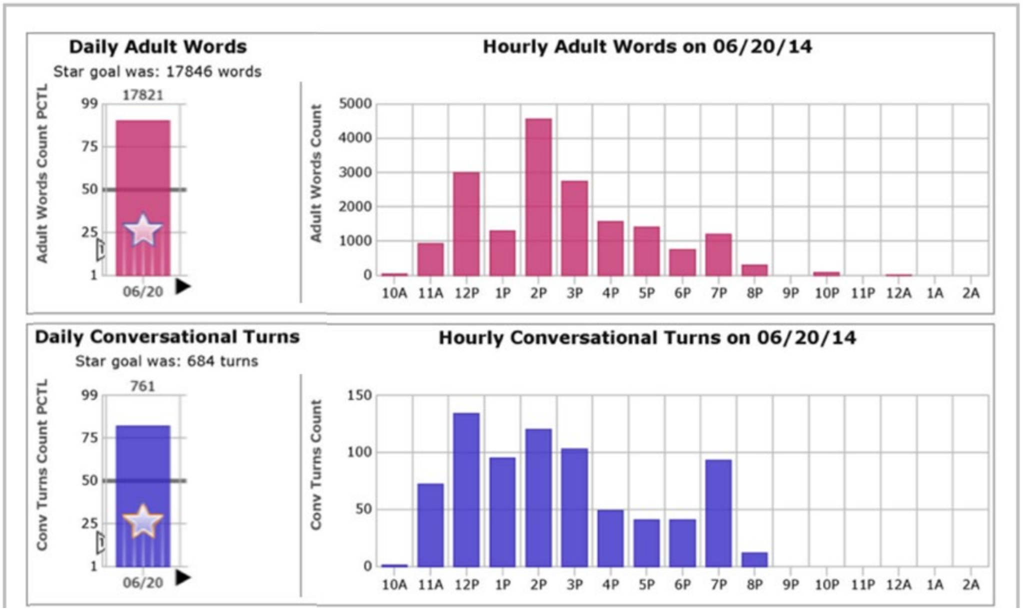

8.2 SAFETY AND OTHER ASSESSMENTS

Because this study is of an educational intervention, study-related adverse events (AEs) and serious adverse events (SAEs) are not expected. Given the minimal risk of the study, the study team will not solicit AEs or SAEs. However, the study team will provide all participants with a phone number to report AEs and SAEs.

The study team will track severe AEs and SAEs that are potentially study related.

8.3 ADVERSE EVENTS AND SERIOUS ADVERSE EVENTS

8.3.1 DEFINITION OF ADVERSE EVENTS (AE)

An AE is defined as any untoward occurrence associated with the use of an intervention in humans, whether or not considered intervention-related (21 CFR 312.32 [a]). We include a list of potential AEs in Section 8.3.3.3; however, we will only record severe AEs (defined below in Section 8.3.3.1).

8.3.2 DEFINITION OF SERIOUS ADVERSE EVENTS (SAE)

We will consider an AE or suspected adverse reaction "serious" if, in the view of the PIs, the medical monitor, the DSMB, or the sponsor (the DCOC of the ECHO ISPCTN), it results in any of the following outcomes:

- Death.
- A life-threatening AE.
- Inpatient hospitalization or prolongation of existing hospitalization.
- Persistent or significant incapacitation or substantial disruption of the ability to conduct normal life functions.

- A congenital anomaly or birth defect.

Important medical events that may not result in death, be life-threatening, or require hospitalization may be considered serious when, based upon appropriate medical judgment, they may jeopardize the participant and may require medical or surgical intervention to prevent one of the outcomes listed in the SAE definition. Examples of such medical events include allergic bronchospasm requiring intensive treatment in an emergency room or at home, blood dyscrasias, convulsions that do not result in inpatient hospitalization, or the development of drug dependency or drug abuse.

8.3.3 CLASSIFICATION OF AN ADVERSE EVENT

8.3.3.1 SEVERITY OF EVENT

These are the guidelines to describe the severity of AEs for the current study:

Mild events require minimal or no treatment and do not interfere with the participant's daily activities.

Moderate events result in a low level of inconvenience or concern with the therapeutic measures. Moderate events may cause some interference with daily functioning.

Severe events interrupt a participant's usual daily activity and may require systemic drug therapy or other treatment. Severe events are usually potentially life threatening or incapacitating. Of note, the term "severe" does not necessarily equate to "serious."

8.3.3.2 RELATIONSHIP TO STUDY INTERVENTION

All severe AEs and SAEs must have their relationship to the trial intervention assessed by the site PI or a qualified clinician, who examines and evaluates the participant based on temporal relationship and their clinical judgment. We will grade the degree of certainty about causality by using the categories below.

Related. We know the AE occurred with the trial intervention, there is a reasonable possibility that the trial intervention caused the AE, or there is a temporal relationship between the trial intervention and the AE. Reasonable possibility means that there is evidence to suggest a causal relationship between the trial intervention and the AE.

Not Related. There is not a reasonable possibility that the administration of the trial intervention caused the event, there is no temporal relationship between the trial intervention and event onset, or there is an established, alternate etiology.

8.3.3.3 EXPECTEDNESS

The site PIs will be responsible for determining whether a severe AE or SAE is expected or unexpected. A severe AE or SAE will be considered unexpected if the nature, severity, or frequency of the event is not consistent with the risk information previously described for the trial intervention.

Potential events could include:

- anxiety
- Respiratory infections:
  - Common cold, influenza, RSV etc.
- Gastroenteritis or associated illness
- Allergies
  - Respiratory, skin, eyes etc.
- Other common infections
  - Eyes, ears, skin, throat, UTIs etc.

8.3.4 TIME PERIOD AND FREQUENCY FOR EVENT ASSESSMENT AND FOLLOW -UP

The study team will provide participants with a telephone number to report severe AEs or SAEs.

Because this minimal-risk study is for an educational intervention, the study will not actively solicit AEs or SAEs. When a site coordinator or site PI learn of a severe AE or SAE, they will refer participants to their primary care physician. The site coordinator will record the severe AE or SAE in the appropriate EDC system and track it.

The site coordinator will record severe AEs, as described in the next section and in the MOP. Severe AE data collection typically includes event description, time of onset, if available, resolution time and day, if available, and operational PI's assessment of severity, relationship to study participation or intervention (assessed only by those with the training and authority to make a diagnosis), and time of resolution/stabilization of the event. The site coordinator must appropriately document severe AEs occurring during the trial, regardless of relationship to the trial. The DCOC safety personnel will track severe AEs until one of the following criteria are met: resolution, the condition stabilizes, the event is otherwise explained or is judged by the site investigator to be no longer clinically significant, or the participant is withdrawn from the study. DCOC safety personnel will follow all SAEs by asking about the SAE every 14 days until resolution or until the site investigator determines the event to be chronic, or until the participant is stable. The DCOC may request other supporting documentation of the event and should be provided as soon as possible.

Site coordinator will document changes in a severe AE in the source document and EDC to allow an assessment of the duration of the event. Severe AEs characterized as intermittent require documentation of onset and duration of each episode.

Participants may report severe AEs for 7 days after completing study procedures and SAEs for 30 days after completing study procedures. Site coordinators and/or DCOC safety personnel will record and track according to this protocol and DCOC standard operating procedures (SOPs).

8.3.5 ADVERSE EVENT REPORTING

Site coordinators or the study team will record severe AEs in the EDC system, include them in the statistical analysis, and follow the reporting procedures outlined in the study-specific MOP. The MOP section on reporting severe AEs will encompass the requirements of:

1. The cIRB's policies and procedures,
2. SOPs for the Streamlined, Multisite, Accelerated Resources for Trials IRB Reliance platform (SMART IRB),

3. ICH E6(R2), Good Clinical Practice: Integrated Addendum to ICH E6(R1): Guidance for Industry, and
4. The local IRB's policies and procedures, when applicable.

If there are discrepancies between the procedures, we will follow the most stringent of the requirements.

If this study is deemed to be greater than minimal risk, or the cIRB otherwise requires a continuing review report, the DCOC will report severe AEs to the cIRB at the time the continuing review is submitted. The DCOC will report to the cIRB at UAMS, as specified in the study-specific IRB communication plan, which is based on recommendations of SMART IRB. Sites will also report severe AEs to the local IRB, per local IRB policies and procedures, and make them available to the sponsor (the DCOC) on a continuing basis through the EDC system. We will report to the DSMB, per the DSMB charter.

The study-specific MOP will describe the details of the reporting structure and additional details related to timelines for reporting.

8.3.6 SERIOUS ADVERSE EVENT REPORTING

The site investigator or qualified delegate will immediately report to the appropriate entities (which may include the DCOC, NIH, DSMB, and local and/or reviewing IRBs) any SAE when required to do so based on that entity's policies and procedures. Reports need to include the information required by the entities policies and procedures. Study endpoints that are SAEs (e.g., hospitalizations) must be reported in accordance with the protocol unless there is evidence suggesting a causal relationship between the study intervention and the event (e.g., death from anaphylaxis). In that case, the investigator must immediately report the event.

All SAEs will be followed until satisfactory resolution or until the site investigator deems the event to be chronic or the participant is stable. Other supporting documentation of the event may be requested by the DDCOC and should be provided as soon as possible.

The DCOC will ensure the summary and report of SAE data to the cIRB in time for consideration at the next continuing review. The DCOC will also create and provide any SAE data summaries requested by the monitors or specified in the DSMB charter. The site PI will be responsible for following their local institution's requirements.

The study-specific MOP has additional details.

8.3.7 REPORTING EVENTS TO PARTICIPANTS

We will notify participants of those trial-related (or potentially trial-related) SAEs that may affect their willingness to continue with the trial or the future health of the primary caregiver or child. Any of the following can determine if study personnel should contact participants: the cIRB, the medical monitor, the DSMB, the DCOC, NIH, or the PIs. The person or oversight body that makes the determination will inform the DCOC, which will instruct the site investigators and coordinators to contact the participants consented through their site.

The site study team will record any contact with participants, if necessary, in the EDC system or trial log.

8.4 UNANTICIPATED PROBLEMS INVOLVING RISKS TO SUBJECTS OR OTHERS (UPIRTSO)

8.4.1 DEFINITION OF UPIRTSO

Unanticipated problems (UPs or UPIRTSOs) involving risks to participants or others include, in general, any incident, experience, or outcome that meets all the following criteria:

- Unexpected in terms of nature, severity, or frequency given (a) the research procedures described in the protocol-related documents, such as the Institutional Review Board (IRB)-approved research protocol and informed consent document; and (b) the characteristics of the participant population being studied;
- Related or possibly related to participation in the research ("possibly related" means there is a reasonable possibility that the incident, experience, or outcome may have been caused by the procedures involved in the research); and
- Suggests that the research places participants or others at a greater risk of harm (including physical, psychological, economic, or social harm) than was previously known or recognized.

A UPIRTSO is not necessarily an AE or an SAE.

Examples of UPs/UPIRTSOs that are not an AE or SAE, but which would need to be reported include:

- Breach of confidentiality
- Manufacturer recall of equipment used in the protocol

The study-specific MOP provides additional details.

8.4.2 UPIRTSO REPORTING

The site investigator will report UPs (UPIRTSOs) – and potential UPIRTSOs - to the DCOC (which will in turn report to the reviewing (UAMS) IRB), to their local IRB as well as to any other persons or groups noted in the DSMB charter. Reports to NIH and the DSMB will be made by following the chain of notification.

Reporting must follow the study-specific IRB communication plan, local IRB policies and procedures, and the UAMS IRB contemporaneous policy, 10.2., *Events that must be reported to the IRB and IRB Actions*, which is available via https://irb.uams.edu/irb-policies/current-irb-policies/.

As of the writing of this section of the protocol (09/12/2023), UAMS IRB reporting requirement per policy 10.2 are:

**UPIRTSO Reporting Requirement**	
**Unanticipated Problem**	**Required Reporting Time to UAMS IRB**
Death or life-threatening	Immediately to IRB office or IRB Chair
All other events	Within 10 days of event or notification of event if non-local

Reports of potential UPIRTSOs typically require the following information:

- Protocol identifying information: protocol title and number, PI's name, and the IRB project number
- Event date
- Event location
- Nature of the risk

- How the risk relates to research
- A detailed description of the event, incident, experience, or outcome
- A description of any changes to the protocol or other corrective actions that have been taken or are proposed in response to the UP

If the UAMS IRB determines the issue is, indeed, a UPIRTSO, the DCOC will notify persons and entities as required by UAMS IRB policies and procedures, the study-specific communication plan, and the DSMB charter.

The cIRB will report the issue to the OHRP, and other entities, per the policies of the cIRB.

8.4.3 REPORTING UNANTICIPATED PROBLEMS TO PARTICIPANTS

For reporting UPIRTSOs to participants, we will follow the same procedures as described for reporting SAEs to participants (see Section 8.3.7).

9 STATISTICAL CONSIDERATIONS

9.1 STATISTICAL HYPOTHESES

Primary Objective: Explore preliminary efficacy of TWMB for enhancing the HLE for at-risk families, specifically by examining whether the language-promotion behaviors of caregivers improve from Baseline to Post-Intervention.

Primary Endpoint: Improvement in language-promotion behaviors (as measured by LENA CTC and AWC percentile scores) from Baseline to Post-Intervention.

Hypothesis: Among caregivers completing TWMB, at least 70% for the sample will demonstrate improvement in language-promotion behaviors from Baseline (Week 1) to Post-Intervention (Week 48).

9.2 SAMPLE SIZE DETERMINATION

The primary endpoint is a binary variable of reaching at least a 10% increase in AWC or CTC percentile scores from Baseline (Week 1) to Post-intervention (Week 48). This increase is based on previous language-promotion intervention studies using LENA, which reported statistically significant gains (for intervention versus control) for parent language-promotion behaviors ranging from 10-30%.[75,118,120-123] In terms of clinical relevance, the proposed 10% gain in AWC would be an increase of ~160 adult words per hour, which equates to an increase of ~3 points in child language standard scores on the Bayley Scales of Infant Development and the Preschool Language Scale (proposed two-point increase for every increase of 100 adult words per hour for children age birth to four years-old).[122,124]

The study is expected to have at least 70% of caregiver participants reaching improvement in language-promotion behaviors at the end of the study. In other words, the targeted rate of improvement in language-promotion behaviors is 70%. We plan a total accrual of up to 86 child-caregiver dyad participants into the study with a goal of having at least 66 dyads enter into phase II of the study. With an estimated phase II attrition rate of 20%, we expect that a total of 53 enrolled child-caregiver dyad participants will complete phase II of the study and hence the effective sample size is 53. Using a two-sided exact test with a significance level of 0.05, we are able to reach 85% power to reject a null rate of improvement at 50%, should the targeted rate of improvement be reached.

9.3 POPULATIONS FOR ANALYSES

The population of interest is the child-caregiver dyads participating in the study.

9.4 STATISTICAL ANALYSES

9.4.1 GENERAL APPROACH

Following finalization of the protocol but prior to data lock, the DCOC statistical team will issue a Statistical Analysis Plan (SAP) as a separate document, which will provide detailed analytical plans for the set of analyses outlined below. The statistical team will conduct all statistical analyses following the statistical principles for

clinical trials as specified in ICH Statistical Principles for Clinical Trials (ICH Topic E9). The study team will describe and justify any deviations from the planned analyses in the final integrated clinical study report. The study team will present all study data and summary tables for the overall study and by study sites.

Descriptive Statistics: All numerical variables will be summarized using mean ± standard deviation and median (minimum, maximum). All categorical variables will be summarized using frequency (in %). Numerical variables will also be inspected of outliers and empirical distributions during data review. Methods for handling outliers and skewed data will be defined and described in the SAP.

Descriptive statistics of the primary endpoints will be reported by caregiver-child demographics (such as race), Medicaid status (yes/no) and WCC visit age (e.g., 2, 4, 6, 9, 15, 18-month WCC visit). The statistics summarized will be used to inform the future RCT.

9.4.2 ANALYSIS OF THE PRIMARY EFFICACY ENDPOINT

The primary endpoint of improvement in caregiver language-promotion behaviors (as measured by LENA) will be summarized using frequency (in %). A 95% confidence interval will be constructed using a Clopper-Pearson exact method. Alternatively, a hypothesized null rate can be tested using a two-sided exact test at a significant level of 0.05.

9.4.3 SAFETY ANALYSES

We will detail all treatment-related severe AEs and SAEs experienced by the child-caregiver dyads. The DCOC will present summary statistics for the study overall and by individual site.

9.4.4 BASELINE DESCRIPTIVE STATISTICS

Baseline characteristics will be summarized using the descriptive statistics specified in **Section 9.4.1**.

9.4.5 PLANNED INTERIM ANALYSES

None.

9.4.6 SUB-GROUP ANALYSES

None.

9.4.7 TABULATION OF INDIVIDUAL PARTICIPANT DATA

The statistical team will provide a description of participants in counts and percentages, including the creation of a Consolidated Standards of Reporting Trials (CONSORT) diagram. De-identified data will also be provided for all study participants.

9.4.8 EXPLORATORY ANALYSES

We will examine barriers to enrollment, including our ability to contact caregivers, the proportion of caregivers that consent to participate in the study, enrollment of caregiver-child dyads, and caregivers' willingness to

complete baseline assessments. We will also examine characteristics related to attrition for caregiver and provider participants. Descriptive statistics will be used in these exploratory analyses.

Caregiver language promotion behaviors (LENA) will be examined and reported descriptively. Child vocalization scores (LENA) will be reported descriptively. We will also examine associations of differences in change in LENA scores with caregiver characteristics (e.g., low, or high baseline LENA scores, education level, SES) using analysis of variance (ANOVA) models or linear regression models.

10 SUPPORTING DOCUMENTATION AND OPERATIONAL CONSIDERATIONS

10.1 REGULATORY, ETHICAL, AND STUDY OVERSIGHT CONSIDERATIONS

10.1.1 INFORMED CONSENT PROCESS

Informed consent is a process that starts before an individual agrees to participate in the trial and continues throughout the individual's trial participation. For this trial, caregiver-child participants include one parent/caregiver plus one child. Once the site and participant complete the process described in this section, the study team will consider the participants to be consented into in the study. All consenting processes must be HIPAA compliant.

10.1.1.1 CONSENT/ASSENT AND OTHER INFORMATIONAL DOCUMENTS PROVIDED TO PARTICIPANTS

Completed (all signatures affixed) consent forms, approved by the reviewing IRB and describing the study intervention, study procedures, and risks, will be given to the participant's parent(s)/legal guardian prior to starting study intervention. The study team must also provide the completed HIPAA form with all signatures affixed. Remote consenting will be allowed per the description below.

10.1.1.2 CONSENT PROCEDURES AND DOCUMENTATION

The contemporaneous version of UAMS IRB policy 15.5, *Informed Consent Process*, is applicable and must be followed. The policy is available at https://irb.uams.edu/irb-policies/current-irb-policies/ If there are any discrepancies between this protocol and applicable reviewing IRB polici(es), the more stringent requirements apply. The relying sites must also follow local IRB policies, as applicable.

Before recruitment begins, all potential caregiver participants will receive an opt-out letter (by mail or via electronic health record, such as MyChart). Returning this letter will allow for potential participants to express their desire to opt out of sharing PHI for prerecruitment screening purposes.

A site coordinator or other study team member will contact potential participants to describe the study and confirm that interested participants meet all inclusion criteria and none of the exclusion criteria (as described in Section 5.5.2).

The study team member then will conduct the informed consent process with participants. They will inform participants that their participation is voluntary, that they may withdraw from the trial at any time without prejudice, and that non-participation will not adversely affect their or their child's medical care.

The study team will collect informed written consent and Health Insurance Portability and Accountability Act (HIPAA) authorization. Site study teams can conduct the informed consent process in person or remotely (as described below). Study staff must complete informed consent procedures, including obtaining a signed informed consent form (ICF) and HIPAA authorization form before any intervention procedures can begin. The study team member must return a signed copy of all ICFs and HIPAA authorization forms to the participants. S.

Study team members will ask participants to read and review the ICF and the HIPAA authorization form. Site study staff will explain the trial to the participant in terms the participant can understand and answer any

questions that may arise. The explanation will state the purposes, procedures, and potential risks of the trial and describe participants' rights as research participants. Participants will have the opportunity to carefully review the ICF and ask questions before signing. The study staff will give participants the opportunity to discuss the trial with their family or surrogates or think about participating in the trial before agreeing to participate. Consenting procedures will conform to the site's and the UAMS cIRB's approved consenting procedures. The study staff will be allowed to accept any of the following as a legal signature on the ICF:

- Signing (wet ink) the ICF and handing it back to the study staff.
- Signing (wet ink) the ICF and mailing (USPS, UPS, FedEx, etc.) it back to the study staff.
- Printing, signing (wet ink), scanning, and emailing or faxing the ICF back to the study staff.
    i. If this method is used, the study staff must tell the participant that their personal (participants) email system may not be secure and that the email could be seen by others and others could find out the parent/child will be in this study.
- Printing, signing, and photographing the signature page of the ICF, and then texting or emailing a picture of the signature page to study staff. Any pictures or scans must include the complete page, include the footer with the version numbering and dates.
    i. If this method is used, the study staff must tell the participant that their personal (participants) email system may not be secure and that the email could be seen by others and others could find out the parent/child will be in this study.
- Using HIPAA-compliant e-signature software to sign the ICF per the procedures established by the relying site.

When sites receive a participant's signed ICF, the study team member who participated in the consent discussion will countersign the ICF and return the countersigned ICF to the participant. The study team member will ask participants to keep a copy of the ICF that is signed by all parties.

The UAMS IRB also requires a consent process note. This policy is 15.5, *Informed Consent Process,* and is available via https://research.uams.edu/irb/policies/current-policies/consent/ :

The person obtaining the consent is required to document the informed consent process in either the subject's research record or medical record. Consent documentation will include, at a minimum:

1. The title of the trial,
2. The date the participant entered the trial,
3. The name of the clinic physician or primary physician,
4. The name of the site investigator,
5. The name of the person(s) obtaining the informed consent,
6. A statement that the participant or LAR a copy of the signed form,
7. A list of who else was present during the consent process,
8. The types of questions asked by the participant,
9. A summary of details that demonstrate the participant understood the consent, and
10. A description of other specific details related to the consent instance as needed.

REMOTE CONSENT

As noted above, remote consenting may be used to enroll participants. All communications will be done via HIPAA-compliant methods such as telephone, personal delivery of documents, US postal service, REDCap or other compliant electronic platform. The remote consent process will parallel the consent processed used for typical in-person consenting; this includes ensuring there is a signed consent and HIPAA authorization prior to starting any study-related procedures. The only difference will be the method(s) of communication. The study team will ensure that, as with in-person consenting, the parent/legal guardian of the dyad is given sufficient opportunity to ask questions, is able to understand the nature of this study and what participation entails, and is provided a copy of the final, completed consent signed by all parties involved, including the research team member who obtained consent and, when applicable, the site investigator. This final, signed consent will be provided via a HIPAA-compliant method or a method that the parent/legal guardian of the participant dyad has agreed to in writing. The site research team members working on the consenting process will ensure that any parent/legal guardian who is consenting remotely has the authority to consent for the child. The study team will also ensure the companion HIPAA research authorization form is completed appropriately and provided to the parent/guardian of the participant dyad.

More specifically, the study team members will contact potential participants to describe the study and, if participants agree, complete the consent process, including verifying eligibility (see Section 5.5.2).

If clinic staff identify potential participants during a regular clinic visit, clinic staff can give or email potential participants study information (e.g., study pamphlet) and can give or email the potential participants' contact information to the study team. The study team may then contact potential participants to describe the study and, if participants agree, complete the consent process, including verifying eligibility (see Section 5.5.2).

IN PERSON CONSENT

The study team anticipates that most participants will provide consent remotely. However, some in-person consents could occur at participating clinics, depending on site and clinic preferences. Study team members will coordinate with clinic staff to schedule a series of days to complete consenting and screening procedures with previously contacted potential participants. If uncontacted but qualifying participants visit the clinic during these days, the study team member may screen, recruit, and consent these individuals in person at the time of the visit or schedule a later time to do so. Study team members will ask participants how they learned about the study.

10.1.2 STUDY DISCONTINUATION AND CLOSURE

Since this is a behavioral intervention trial, the study team does not expect any intervention-related safety events causing the protocol chair and co-chair, the medical monitor, the DSMB, or the cIRB to suspend or stop the trial.

The trial may be suspended or stopped per any suspension or stopping specifications in the DSMB charter. The cIRB may also stop or suspend the trial. Early termination of the study may be permanent if there is sufficient cause. The suspending or terminating party will provide, directly or indirectly, written notification documenting the reason for trial suspension or termination to the following, as applicable: trial participants, the protocol chair and co-chair, site PIs, the cIRB, local IRBs, the NIH, the DCOC, and the OHRP. Persons and offices notified will

include those specified in the study-specific MOP, the cIRB's policies and procedures, and the SMART IRB policies and procedures.

The suspending or terminating party will also contact trial participants and inform them of any changes that affect participation. Circumstances that may warrant trial termination or suspension include (but are not limited to):

- Determination of unexpected, significant, or unacceptable risk to participants.
- Insufficient compliance to protocol requirements.
- Data that are not sufficiently complete or evaluable
- Evidence of study futility of the primary endpoint

If the trial is suspended, it may resume once concerns about safety, protocol compliance, and data quality are addressed and are satisfactory to the DSMB, the cIRB, the local IRBs (when applicable), the NIH, and the DCOC.

10.1.3 CONFIDENTIALITY AND PRIVACY

Sites and study team members will conduct all trial activities in as private a manner as possible.

Records will be maintained as required by the privacy and security rules promulgated by the HIPAA (45 CFR 164).[125,126]

During the trial, site investigators and coordinators will keep all trial records in secure locations that only authorized personnel can access. Examples of secure locations include but are not limited to: 1) locked file cabinet(s) in a limited (badge or key) access room, or 2) password-protected computer systems. Study personnel may only transmit records that contain PHI, as defined by HIPAA, through an open email system if the personnel encrypt the data. Password protection alone is insufficient for data transmission through an open email system (e.g., Outlook). After trial completion, access to trial records will be limited (see Section 10.1.4).

Certain bodies or institutions may need to review information, including participant information, for any of the following reasons: to process information or to ensure compliance with the protocol and other applicable requirements (such as the policies and procedures of the cIRB). Institutions or bodies that may have access to the participants' information include, but are not necessarily limited to:

- The UAMS IRB and other oversite offices.
- The IRB for the site through which the participant is consented.
- Oversite offices at participating sites.
- The US OHRP.
- The ISPCTN DCOC.
- The NIH.

Individuals with access to trial records may include, but are not necessarily limited to:

- The protocol chair and co-chair.
- Site investigators.
- Site coordinators.
- Data managers at participating sites.

- Study monitors (which may include DCOC personnel and personnel employed by contracted monitoring groups)
- Auditors

10.1.4 MULTI-SITE COMMUNICATIONS (IRB-RELATED)

This study will be conducted at various sites (2 enrolling sites) within the ISCPTN network. All sites will cede to a cIRB (per SMART IRB definitions). The study-specific IRB-related communications plan was constructed from the SMART IRB template and uses SMART IRB recommendations for communications. This plan will be submitted to the IRB as a separate study-specific document. The DCOC will serve as the lead study team and will be the intermediary between the sites and the UAMS IRB as the central (or single) IRB (i.e., cIRB). Other types of communications (i.e., related to data, study deviations, etc.) between DCOC and the sites are detailed in their respective appropriate sections of this protocol.

10.1.4 FUTURE USE OF STORED SPECIMENS AND DATA

No specimens will be collected or stored for this trial. Regarding stored data, study personnel will document all trial interactions, and these will be password-protected in a secured facility or location.

The study team will place participant's de-identified data and other limited information, such as race and ethnic group, into one or more centralized database(s). The study team will share this data in compliance with the NIH data sharing policy.

For future studies using any procedures or analysis not specified in this protocol, cIRB approval is required.

If another investigator or collaborator has a meaningful purpose for accessing the data retrieved in this protocol, the DCOC must consult the PIs, and the cIRB must approve.

10.1.5 KEY ROLES AND STUDY GOVERNANCE

**Protocol Chair**	
Name, degree, title	Brenda Salley, PhD, Associate Professor, Pediatrics
Institution	University of Kansas Medical Center
Address	3901 Rainbow Boulevard, Kansas City, KS 66160
Phone Number	(913) 945-7944
Email	bsalley@kumc.edu
**Protocol Co-Chairs**	
Name, degree, title	David Huss, MD
Institution	West Virginia University
Address	1 Medical Center Dr., Morgantown, WV 26056
Phone Number	304-293-7331
Email	david.huss@hsc.wvu.edu

Name, degree, title	Margaret Jaynes, MD
Institution	West Virginia University
Address	1 Medical Center Dr., Morgantown, WV 26056
Phone Number	304-293-7331
Email	mjaynes@hsc.wvu.edu
**DCOC (Overall, cIRB) PI**	
Name, degree, title	Songthip Ounpraseuth, PhD, Professor, College of Public Health
Institution	University of Arkansas for Medical Sciences
Address	4301 West Markham, # 820, Little Rock, AR 72205
Phone Number	(501) 686-7233
Email	STOunpraseuth@uams.edu
**Statistician**	
Name, degree, title	Jing Jin, PhD, Instructor, Biostatistics
Institution	University of Arkansas for Medical Sciences
Address	4301 West Markham, # 820, Little Rock, AR 72205
Phone Number	(501) 214-2236
Email	JJin@uams.edu
**DCOC Co-PIs**	
Name, degree, title	Fred Prior, PhD<br>Distinguished Professor and Chair Dept. Biomedical Informatics and Professor of Radiology
Institution	University of Arkansas for Medical Sciences
Address	4301 W. Markham St., Slot 782<br>Little Rock, AR 72205
Phone Number	(314) 303-2485
Email	FWPrior@uams.edu
**Medical Monitor**	
Name, degree, title	Sherry E. Courtney, MD; Professor of Pediatrics and Director of Clinical Research for Neonatology
Institution	University of Arkansas for Medical Sciences / Arkansas Children's Hospital
Address	One Children's Way<br>Little Rock, AR 72202
Phone Number	501-364-1028
Email	scourtney@uams.edu

The clinical trial outlined in this protocol is part of the ECHO ISPCTN, a branch of the program supported by the NIH.

The data coordination, technical instruction, data standards, quality control and quality assurance, and operational coordination for the clinical trial protocol outlined here (and for the ECHO ISPCTN overall) is provided by the DCOC.

The Steering Committee governs the ECHO ISPCTN and includes representatives from all site awardees, as well as representatives from the DCOC and the NIH. Overseeing the work of the Steering Committee is the NIH ECHO office, as well as an executive Leadership Committee.

A team of content experts and DCOC staff completed this protocol, and investigators within ECHO ISPCTN leadership reviewed it. The NIH Protocol Review Committee and the DSMB further reviewed the protocol.

10.1.6 SAFETY OVERSIGHT

Ensuring participant safety is the responsibility of all study team members, especially the protocol chair and co-chair, site investigators, site coordinators, research team members, and monitors. A medical monitor and the DSMB will provide oversight.

The medical monitor will be a pediatrician with expertise in child development and will be independent of the trial. The NIH will convene the DSMB, and it will meet regularly, according to its charter.

The entities that will receive reports include, but are not limited to, the DCOC and the NIH. The DSMB charter will provide additional information.

10.1.7 CLINICAL MONITORING

We will conduct clinical site monitoring to ensure that site PIs, site coordinators, and research team members are protecting the rights and well-being of trial participants, that the reported trial data are accurate, complete, and verifiable, and that the conduct of the trial complies with the currently approved protocol and its amendments, with ICH GCP, and with applicable regulatory requirement(s).

- A DCOC team member or designee will remotely monitor site awardees to ensure data quality and integrity.
- On-site monitoring visits will also be performed at each site, per the Site Monitoring Plan (SMP), if needed for cause.
- The details of the clinical site monitoring are documented in the SMP (e.g., who will conduct the monitoring, at what frequency, at what level of detail monitoring will be performed, and the distribution of monitoring reports).

10.1.8 QUALITY ASSURANCE AND QUALITY CONTROL

Each cIRB-approved research site entering data will perform internal quality management of study conduct, data collection, documentation, and completion. Sites that ceded to the UAMS IRB (as central IRB) will follow applicable UAMS IRB policies, available at https://irb.uams.edu/irb-policies/current-irb-policies/ . A listing of applicable UAMS IRB policies will be provided in the MOP. If local requirements conflict with UAMS IRB policies, sites will consult with DCOC to help determine which policies and procedures need to be followed. Each site will follow the study-specific MOP and any additional site-specific SOPs.

Sites will provide direct access to all their facilities, source data and documents, and reports for the purpose of monitoring and auditing by the DCOC and inspection by local and regulatory authorities. When electronic health

records are source data and documents, sites must provide read-only access for the monitors, auditors, and anyone else authorized to inspect or verify records.

Following the DCOC monitoring SOPs, the monitors will verify that the clinical trial is conducted, and that data are generated, documented (recorded), and reported in compliance with:

1. The protocol,
2. The study-specific SMP,
3. Site-specific SOPs,
4. The ICH GCP E6(R2), and
5. Applicable regulatory requirements.

The DCOC will implement quality control procedures for the database and DCOC-maintained records in accordance with the study-specific SMP, MOP, and data safety monitoring plan (DSMP) and any applicable SOPs. The DCOC may communicate information about any data anomalies to the sites for clarification or resolution.

The DCOC will address issues uncovered during quality assurance or monitoring activities through simple corrections or root-cause analysis, followed by instituting corrective and preventative action (CAPA), as appropriate and as described in the study-specific MOP.

10.1.9 DATA HANDLING AND RECORD KEEPING

10.1.9.1 DATA COLLECTION AND MANAGEMENT RESPONSIBILITIES

A formal data management plan will describe and document the data and workflow for the trial. The data management plan and associated documentation will specify all operations performed on data from origination to database lock, including detailed descriptions of

- Source documentation.
- Case report forms (CRFs).
- Instructions for completing CRFs.
- Data-handling and record-keeping procedures.
- Procedures for data monitoring.
- Reconciliation procedures and coding dictionaries to be used, if applicable.

The data management plan will also describe the specific data collection and management responsibilities required of the DCOC, the protocol chair and co-chair, site PIs, site awardees, and participating clinics. The data management plan contents will be consistent with those described in the Good Clinical Data Management Practices. The DCOC will provide the data management plan components that document operations performed on the data to the protocol chair and co-chair for review and approval prior to implementation.

Data collection is the responsibility of the research staff at the individual sites under the supervision of the site investigator. The site investigator is responsible for ensuring the accuracy, completeness, legibility, and timeliness of the data reported. All source documents must be completed using standard good documentation practices (i.e., the ALCOA-C method [attributable, legible, contemporaneous, original, accurate and complete]).

It is best practice for site coordinators to use hardcopies of any data recorded on paper CRFs or trial visit worksheet or assessment forms as source documents for recording data for each participant consented. Data recorded in the EDC system derived from source documents must be consistent with the data recorded on the source documents.

Site study team personnel will enter data (including demographics and intervention-specific questionnaires) into an EDC system that complies with HIPAA regulations, provided by the DCOC. The EDC system includes password protection and internal quality checks, such as automatic range checks, to identify data that appear inconsistent, incomplete, or inaccurate. Study staff will enter clinic data directly from the source documents.

10.1.9.2 STUDY RECORDS RETENTION

Throughout the course of the trial, all sites will retain the source documents on site in accordance with current site-specific storage procedures for medical records.

Sites must retain all trial documents in accordance with local or federal regulations, whichever is most stringent. Sites will not destroy any records without the written consent of the DCOC. The DCOC will inform all site investigators when they no longer need to retain these documents.

10.1.10 PROTOCOL DEVIATIONS

A deviation is any instance of failure to follow, intentionally or unintentionally, the requirements of the clinical trial protocol, ICH E6(R2) (i.e., "GCP"), the study-specific MOP, or other documents needed to complete study conduct. The instance of failure may be on the part of the participant, the investigator, or other study staff personnel. When deviations occur, the sponsor and/or site team(s) will ensure actions are taken to correct the problem and, as needed, prevent the deviation from recurring.

These practices are consistent with ICH E6(R2) (available at https://www.fda.gov/regulatory-information/search-fda-guidance-documents/e6r2-good-clinical-practice-integrated-addendum-ich-e6r1). Specifically, sections:

- 4.5 Compliance with Protocol, sections 4.5.1, 4.5.2, and 4.5.3
- 5.1 Quality Assurance and Quality Control, section 5.1.1
- 5.20 Noncompliance, sections 5.20.1, and 5.20.2.

Sites must record all deviations in the trial source documents. Whenever a deviation occurs, the DCOC will ensure an appropriate assessment is conducted. The assessment should include documentation of the severity and risk of the deviation. Sites that have a system set up for assessing deviations and doing their own corrections via corrective and preventive action (CAPA) plans will do so according to their site SOPs/system. The site will send copies of their CAPA plan documentation to the DCOC. If a site does not have their own quality assurance system to complete adequate deviation review and assessments, corrections, and CAPA plans, then the DCOC will provide that function for the sites. Details of these processes will be provided in the MOP and/or trial-specific SOPs. Essentially, the site and/or the DCOC will request/ensure that there is either a CAPA plan initiated, or a simple one-time correction is performed, as appropriate.

10.1.11 PUBLICATION AND DATA SHARING POLICY

We will conduct this trial in accordance with the following publication and data sharing policies and regulations:

- **NIH Public Access Policy**, which ensures that the public has access to the published results of NIH-funded research. It requires scientists to submit final, peer-reviewed journal manuscripts that arise from NIH funds to the digital archive PubMed Central upon acceptance for publication.
- **ECHO ISPCTN Publications and Presentations Policy**, which ensures accurate, responsible, and efficient communication of findings from ECHO ISPCTN clinical trials. The ECHO ISPCTN Steering Committee, which includes representatives from all site awardees, the NIH, and the DCOC, has approved and ratified the Publications and Presentations Policy.
- **NIH Data Sharing Policy and the policy on the Dissemination of NIH-Funded Clinical Trial Information and the Clinical Trials Registration and Results Information Submission Rule**. We will register this trial at ClinicalTrials.gov, and we will submit trial results to ClinicalTrials.gov. In addition, we will make every attempt to publish results in peer reviewed journals. Other researchers may request data from this trial by contacting Songthip Ounpraseuth, PhD, at the DCOC.

10.1.12 CONFLICT OF INTEREST POLICY

The independence of this trial from any actual or perceived influence, such as by the pharmaceutical industry, is critical. Therefore, we will disclose and manage any actual conflict of interest of persons who have a role in the design, conduct, analysis, publication, or any aspect of this trial. Furthermore, persons who have a perceived conflict of interest will be required to have such conflicts managed in a way that is appropriate to their participation in the design and conduct of this trial. The trial leadership in conjunction with the NIH ECHO office has established policies and procedures for all trial group members to disclose all conflicts of interest and will establish a mechanism for the management of all reported dualities of interest.

10.2 ADDITIONAL CONSIDERATIONS

None.

10.3 GLOSSARY

Caregiver – a person that has legal guardianship and legal authority to make medical decisions, including medical research, for their child as well as for themselves. The “caregiver” may be a biological or adoptive parent or another legally authorized representative (LAR) as defined by applicable state laws.

Enrolled – A participant who is consented into the study (i.e., has signed a consent and HIPAA authorization form). Enrolled and consented are synonymous terms.

Follow up – The intervention phase of the study.

Participating clinic – The clinic that is implementing the TWMB intervention.

Phase 1 participants – Participants who are consented and sent the LENA device for baseline recording.

Phase 2 participants – Participants whose baseline score qualifies them for the study (i.e., ≤75th percentile) and participant enters follow-up.

Protocol study team – The study PIs/protocol chairs, their mentors, site coordinators, and DCOC leadership.

Site study team – The site PI and site coordinator(s) who recruit and enroll participants into the study, along with others from the site who are on the Delegation of Authority Log.

10.4 ABBREVIATIONS

AAP	American Academy of Pediatrics
AE	Adverse Event
ANCOVA	Analysis of Covariance
AWC	Adult Word Count
CAB	Community Advisory Board
CAPA	Corrective and Preventative Action
CFR	Code of Federal Regulations
cIRB	Central Institutional Review Board
CONSORT	Consolidated Standards of Reporting Trials
CRF	Case Report Form
CTC	Conversational Turn Count
DCOC	Data Coordinating and Operations Center
DSMB	Data Safety Monitoring Board
DSMP	Data Safety Monitoring Plan
DUA	Data Use Agreement
ECHO	Environmental influences on Child Health Outcomes
EHR	Electronic Health Record
FDA	Food and Drug Administration
GCP	Good Clinical Practice
HIPAA	Health Insurance Portability and Accountability Act
HLE	Home Language Environment
HRSA	Health Resources and Services Administration
ICF	Informed Consent Form
ICH	International Council on Harmonisation
IRB	Institutional Review Board
ISPCTN	IDeA States Pediatric Clinical Trials Network
LENA	Language ENvironment Analysis System
MOP	Manual of Operating Procedures
MSDS	Material Safety Data Sheet
NICU	Neonatal Intensive Care Unit
NIH	National Institutes of Health
OB/GYN	Obstetrics and Gynecology
OHRP	Office for Human Research Protections
PAB	Provider Advisory Board

PHI	Protected Health Information
PI	Principal Investigator
RCT	Randomized Controlled Trial
ROR	Reach Out and Read
RUCA	Rural-Urban Commuting Area
SAE	Serious Adverse Event
SAP	Statistical Analysis Plan
SES	Socioeconomic Status
SMART IRB	Streamlined, Multisite, Accelerated Resources for Trials IRB Reliance platform
SMP	Site Monitoring Plan
SOP	Standard Operating Procedure
TWMB	Talk With Me Baby
UAMS	University of Arkansas for Medical Sciences
UPIRTSO	Unanticipated Problem Involving Risk to Subjects or Others
US	United States
VIP	Video Interaction Project
WCC	Well-child Care
WIC	Special Supplemental Nutrition for Women, Infants, and Children

11 REFERENCES

1. Dickinson DK, Porche MV. Relation Between Language Experiences in Preschool Classrooms and Children's Kindergarten and Fourth-Grade Language and Reading Abilities. *Child Development*. 2011;82(3):870-886. doi:0.1111/j.1467-8624.2011.01576.x
2. Hart B, Risley TR. *Meaningful differences in the everyday experience of young American children*. Baltimore, MD; 1995.
3. Hernandez DJ. *Double jeopardy: How third-grade reading skills and poverty influence high school graduation*. 2012. http://www.aecf.org/m/resourcedoc/AECF-DoubleJeopardy-2012-Full.pdf
4. National Center for Health Statistics. *Health, United States 2011: with special features on socioeconomic status and health. Washington, D.C: U.S.Government Printing Office*. 2012:2012-1232.
5. Robert Wood Johnson Foundation. *Education matters for health (Vol. 6) Robert Wood Johnson Foundation.* 2009:1-15.
6. Leger M, Roberts C, Sharp S. *The road to readiness: the precursors and practices that predict school readiness and later school success*. Overdeck Family Foundation; 2020.
7. Rowe ML. A longitudinal investigation of the role of quantity and quality of child-directed speech in vocabulary development. *Child Dev*. Sep-Oct 2012;83(5):1762-74. doi:10.1111/j.1467-8624.2012.01805.x
8. Weisleder A, Fernald A. Talking to children matters: early language experience strengthens processing and builds vocabulary. *Psychol Sci*. Nov 1 2013;24(11):2143-52. doi:10.1177/0956797613488145
9. Hoff E. The specificity of environmental influence: Socioeconomic status affects early vocabulary development via maternal speech. *Child Development*. 2003;74(5):1368-1378.
10. Melvin SA, Brito NH, Mack LJ, et al. Home Environment, But Not Socioeconomic Status, is Linked to Differences in Early Phonetic Perception Ability. *Infancy*. Jan-Feb 2017;22(1):42-55. doi:10.1111/infa.12145
11. Romeo RR. Beyond the 30-million word gap: Children's conversational exposure is associated with language-related brain function. *Psychol Sci*. 2018;29(5):700-710. doi:10.1177/0956797617742725
12. Child Trends Databank. Children in poverty. www.childtrends.org-poverty. 2016;
13. Centers for Disease Control and Prevention. Ending poverty now Report available at:. http://www.childrensdefense.org/library/PovertyReport/ EndingChildPovertyNow.html. 2018;
14. Heckman JJ. Schools, skills and synapses. *Economic Inquiry*. 2008;46:289–324. doi:http://dx.doi.org/10.1111/j.1465-7295.2008.00163.x
15. Housing Assistance Council. *Poverty in Rural America. Rural research brief*. 2012. http://ruralhome.nonprofitsoapbox.com/storage/research_notes/rrn_poverty.pdf
16. United States Department of Agriculture Economic Research Service. *Rural poverty & well-being*. 2020. https://www.ers.usda.gov/topics/rural-economy-population/rural-poverty-well-being/
17. Laurore J. *Health care access for infants and toddlers in rural areas*. 2020. *Child Trends*. https://www.childtrends.org/publications/health-care-access-for-infants-and-toddlers-in-rural-areas
18. De Marco A, Vernon-Feagans L. Rural Neighborhood Context, Child Care Quality, and Relationship to Early Language Development. *Early Educ Dev*. Aug 2013;24(6):792-812. doi:10.1080/10409289.2013.736036
19. Burchinal M, Vernon-Feagans L, Cox M, Key Family Life Project I. Cumulative Social Risk, Parenting, and Infant Development in Rural Low-Income Communities. *Parent Sci Pract*. 2008;8(1):41-69. doi:10.1080/15295190701830672
20. Schaefer A, Mattingly MJ, Johnson KM. *Child poverty higher and more persistent in rural america*. Vol. National Issue Brief #97. 2016. *Casey Research*.
21. Brito NH. Influence of the Home Linguistic Environment on Early Language Development. *Policy Insights from the Behavioral and Brain Sciences*. 2017;4(2):155-162. doi:10.1177/2372732217720699
22. Rosenberg SA, Zhang D, Robinson CC. Prevalence of developmental delays and participation in early intervention services for young children. *Pediatrics*. Jun 2008;121(6):e1503-9. doi:10.1542/peds.2007-1680

23. Suskind DL, Leffel KR, Graf E. A parent-directed languge intervention for children of low socioeconomic status: A randomized controlled pilot study. *Journal of Child Language*. 2016;43:366-406. doi:10.1017/S0305000915000033
24. Roberts MY, Kaiser AP. The effectiveness of parent-implemented language interventions: A meta-analysis. *American Journal of Speech Language Pathology*. 2011;20:180-199. doi:10.1044/1058-0360(2011/10-0055
25. Heidlage JK, Cunningham JE, Kaiser AP, et al. The effects of parent-implemented language interventions on child linguistic outcomes: A meta-analysis. *Early Childhood Research Quarterly*. 2020;50:6-23. doi:10.1016/j.ecresq.2018.12.006
26. Walker D, Carta JJ. Intervention research to improve language-learning opportunities and address the inequities of the word gap. *Early Childhood Research Quarterly*. 2020;50:1-5. doi:10.1016/j.ecresq.2019.10.008
27. Greenwood CR, Schnitz AG, Carta JJ, Wallisch A, Irvin DW. A systematic review of language intervention research with low-income families: A word gap prevention perspective. *Early Childhood Research Quarterly*. 2020;50:230-245. doi:10.1016/j.ecresq.2019.04.001
28. Darcy Mahoney A, McConnell SR, Larson AL, Becklenberg A, Stapel-Wax JL. Where do we go from here? Examining pediatric and population-level interventions to improve child outcomes. *Early Childhood Research Quarterly*. 2020;50:205-220. doi:10.1016/j.ecresq.2019.01.009
29. National Center for Health Statistics. *Percentage of having a well child check-up in the past 12 months for children under age 18 years, United States, 2019. National Health Interview Survey. Generated interactively: Jan 25 2022 from https://wwwn.cdc.gov/NHISDataQueryTool/SHS_2019_CHILD3/index.html*.
30. Lipkin PH, Macias MM, Pediatrics COCWDSODaB. Promoting Optimal Development: Identifying Infants and Young Children With Developmental Disorders Through Developmental Surveillance and Screening. *Pediatrics*. 2020;145(1):e20193449.
31. Council on Children With Disabilities; Section on Developmental Behavioral Pediatrics; Bright Futures Steering Committee; Medical Home Initiatives for Children With Special Needs Project Advisory Committee. Identifying infants and young children with developmental disorders in the medical home: an algorithm for developmental surveillance and screening. *Pediatrics*. 2006;118(1):405–420.
32. American Academy of Pediatrics. *Bright Futures: Guidelines for Health Supervision of Infants, Children, and Adolescents*. 4th ed. 2017.
33. Committee on Practice and Ambulatory Medicine; Bright Futures Periodicity Schedule Workgroup. 2019 recommendations for preventive pediatric health care. *Pediatrics*. 2019;143(3):e20183971.
34. Stapel-Wax JL, Darcy Mahoney AE, Fitzgerald B, Moore K, Weldon A, Yates C. Talk With Me Baby: A curriculum for nurses. 2016.
35. Brasher S. Integrating early brain science and skills into prelicensure nursing curriculum to promote parent-child interaction. *Nurse Educator*. 2021;
36. Gaines T, Ryan T, Williams B, et al. Talk With Me Baby: Evaluating the effectiveness of training workforces on the primacy of language nutrition. presented at: Poster session presented at the Society for Research in Child Development 2017 Biennial Meeting; 2017;
37. Salley B, Carta J, Walker C, Weltmer K. Talk With Me Baby: Universal Language Promotion in Primary Care. presented at: Biennial meeting of the International Congress on Infant Studies (ICIS); 2020; Virtual Meeting.
38. Arnold DM, Burns KE, Adhikari NK, et al. The design and interpretation of pilot trials in clinical research in critical care. *Crit Care Med*. Jan 2009;37(1 Suppl):S69-74. doi:10.1097/CCM.0b013e3181920e33
39. Pearson N, Naylor PJ, Ashe MC, Fernandez M, Yoong SL, Wolfenden L. Guidance for conducting feasibility and pilot studies for implementation trials. *Pilot Feasibility Stud*. Oct 31 2020;6(1):167. doi:10.1186/s40814-020-00634-w

40. Snowden J, Darden P, Palumbo P, Saul P, Lee J, Network IDSPCT. The institutional development award states pediatric clinical trials network: building research capacity among the rural and medically underserved. *Current opinion in pediatrics*. Apr 2018;30(2):297-302. doi:10.1097/MOP.0000000000000597

41. The Annie E. Casey Foundation. *KIDS COUNT Data Book: State Trends in Child Well-Being*. 2017.

42. United States Department of Agriculture Economic Research Service. *Rural Poverty & Well-Being*. April 19, 2018. https://www.ers.usda.gov/topics/rural-economy- population/rural-poverty-well-being

43. Gilkerson J, Richards JA, Warren SF, Oller DK, Russo R, Vohr B. Language Experience in the Second Year of Life and Language Outcomes in Late Childhood. *Pediatrics*. Oct 2018;142(4)doi:10.1542/peds.2017-4276

44. Rowe ML, Raudenbush SW, Goldin-Meadow S. The pace of vocabulary growth helps predict later vocabulary skill. *Child Development*. Mar-Apr 2012;83(2):508-25. doi:10.1111/j.1467-8624.2011.01710.x

45. Aikens NL, Barbarin O. Socioeconomic differences in reading trajectories: The contribution of family, neighborhood, and school contexts. *Journal of Educational Psychology*. 2008;100(2):235–251.

46. Rodriguez ET, Tamis-LeMonda CS. Trajectories of the home learning environment across the first 5 years: Associations with children's vocabulary and literacy skills at prekindergarten. *Child Development*. 2011;82(4):1058–1075.

47. Hoff E. Interpreting the early language trajectories of children from low SES and language minority homes: Implications for closing the achievement gap. *Dev Psychol*. 2013;49(4):4–14. doi:10.1037/a0027238

48. Tamis-LeMonda CS, Bornstein MH, Baumwell L. Maternal responsiveness and children's achievement of langauge milestones. *Child Development*. 2001;72(3):748-767. doi:10.1111/1467-8624.00313

49. Brito NH. Influence of the home linguistic environment on early language development. *Policy Insights from the Brain and Behavioral Sciences*. 2017:1-8. doi:10/1177/2372732217720699

50. Perani D, Saccuman MC, Scifo P, et al. Neural language networks at birth. *Proceedings of the National Academy of Sciences*. Sep 20 2011;108(38):16056-61. doi:10.1073/pnas.1102991108

51. Kisilevsky BS, Hains SM, Brown CA, et al. Fetal sensitivity to properties of maternal speech and language. *Infant Behavior and Development*. Jan 2009;32(1):59-71. doi:10.1016/j.infbeh.2008.10.002

52. Shonkoff JP, Phillips DA. *From neurons to neighborhoods: The science of early childhood development*. National Academy Press; 2000.

53. Hoff E, Naigles L. How children use input in acquiring a lexicon. *Child Development*. 2002;73:418–433.

54. Kuhl PK. Brain mechanisms in early language acquisition. *Neuron*. 2010;67(5):713-27. doi:10.1016/j.neuron.2010.08.038

55. Hutton JS, Horowitz-Kraus T, Mendelsohn AL, DeWitt T, Holland SK, Consortium. C-MA. Home reading environment and brain activation in preschool children listening to stories. *Pediatrics*. 2015;136(3):466-478. doi:10.1542/peds.2015-0359

56. Hackman DA, Farah MJ, Meaney MJ. Socioeconomic status and the brain: mechanistic insights from human and animal research. *Nature Reviews Neuroscience*. Sep 2010;11(9):651-9. doi:10.1038/nrn2897

57. Kuhl PK, Tsao FM, Liu HM. Foreign-language experience in infancy: effects of short-term exposure and social interaction on phonetic learning. *Proc Natl Acad Sci U S A*. Jul 22 2003;100(15):9096-101. doi:10.1073/pnas.1532872100

58. Biermiller A, Slomin N. Estimating root word vocabulary growth in normative and advantaged populations: Evidence for a common sequence of vocabulary acquisition. *Journal of Educational Psychology*. 2001;93(3):498-520. doi:10.1037/0022-0663.93.3.498

59. Huttenlocher J, Waterfall H, Vasilyeva M, Vevea J, Hedges LV. Sources of variability in children's language growth. *Cognitive Psychology*. 2010;61(4):343–365.

60. Rowe ML. Child-directed speech: relation to socioeconomic status, knowledge of child development and child vocabulary skill. *Journal of Child Language*. 2008;35(01)doi:10.1017/s0305000907008343

61. Pace A, Luo R, Hirsh-Pasek K, Golinkoff RM. Identifying pathways between socioeconomic status and language development. *Annual Review of Linguistics*. 2017;3:285–308.

62. Rowe ML. Understanding socioeconomic differences in parents' speech to children. *Child Development Perspectives*. 2017;12:122–127.
63. Rowe ML, Denmark N, Harden BJ, Stapleton LM. The Role of Parent Education and Parenting Knowledge in Children's Language and Literacy Skills among White, Black, and Latino Families. *Infant and Child Development*. 2016;25(2):198-220. doi:10.1002/icd.1924
64. Suskind DL, Leung CYY, Webber RJ, et al. Educating Parents About Infant Language Development: A Randomized Controlled Trial. *Clinical Pediatrics*. Jul 2018;57(8):945-953. doi:10.1177/0009922817737079
65. Fernald A, Marchman VA, Weisleder A. SES differences in language processing skill and vocabulary are evident at 18 months. *Developmental Science*. Mar 2013;16(2):234-48. doi:10.1111/desc.12019
66. Hurtado N, Marchman VA, Fernald A. Does input influence uptake? Links between maternal talk, processing speed and vocabulary size in Spanish-learning children. *Developmental Science*. 2008;11(6):31–39. doi:doi:10.1111/j.1467-7687
67. Romeo RR, Leonard JA, Grotzinger HM, et al. Neuroplasticity associated with changes in conversational turn-taking following a family-based intervention. *Dev Cogn Neurosci*. Jun 2021;49:100967. doi:10.1016/j.dcn.2021.100967
68. Nelson KE, Welsh JA, Trup EMV, Greenberg MT. Language delays of impoverished preschool children in relation to early academic and emotion recognition skills. *First Language*. 2011;31(2):164-194. doi:10.1177/0142723710391887
69. Greenwood CR, Carta JJ, Walker D, et al. Conceptualizing a Public Health Prevention Intervention for Bridging the 30 Million Word Gap. *Clinical Child and Familly Psychological Review*. Mar 2017;20(1):3-24. doi:10.1007/s10567-017-0223-8
70. Crow S, O'Leary A. *Word health: Addressing the word gap as a public health crisis.* 2015, May 1. *Next Generation*. Retrieved from http://thenextgeneration.org/publications/word-health-addressing-the-word-gap-as-a-public-health-crisis
71. Carta J, Greenwood CR, Walker D. Bridging the Word Gap Research Network (BWGRN). Health Resources and Services Administration. https://bwg.ku.edu/. 2021.
72. Kong N, Carta JJ. Responsive interaction interventions for children at risk for and with developmental delays. *Topics in Early Childhood Special Education*. 2013;33(1):4–17.
73. Peterson P, Carta JJ, Greenwood CR. The effects of teaching enhanced milieu language teaching skills to parents in multiple risk families. *Journal of Early Intervention*. 2005;27(2):94–109.
74. Leung CYY, Hernandez MW, Suskind DL. Enriching home language environment among families from low-SES backgrounds: A randomized controlled trial of a home visiting curriculum. *Early Childhood Research Quarterly*. 2020;50:24-35. doi:10.1016/j.ecresq.2018.12.005
75. Ferjan Ramirez N, Lytle SR, Kuhl PK. Parent coaching increases conversational turns and advances infant language development. *Proceedings of the National Academy of Sciences*. Feb 18 2020;117(7):3484-3491. doi:10.1073/pnas.1921653117
76. Ramirez-Esparza N, Garcia-Sierra A, Kuhl PK. Look Who's Talking NOW! Parentese Speech, Social Context, and Language Development Across Time. *Frontiers in psychology*. 2017;8:1008. doi:10.3389/fpsyg.2017.01008
77. Cates CB, Weisleder A, Mendelsohn AL. Mitigating the Effects of Family Poverty on Early Child Development through Parenting Interventions in Primary Care. *Academic Pediatrics*. Apr 2016;16(3 Suppl):S112-20. doi:10.1016/j.acap.2015.12.015
78. United States Department of Agriculture. *Economic Research Service 2021 Annual Report.*
79. Kuhl PK. Early Language Learning and Literacy: Neuroscience Implications for Education. *Mind Brain Educ*. Sep 2011;5(3):128-142. doi:10.1111/j.1751-228X.2011.01121.x

80. Irwin LG, Siddiqi A, Hertzman C. *Early childhood development: A powerful equalizer. Final report for the World Health Organizations' Commission on the Social Determinents of Health. Retrieved from http://apps.who.int/iris/bitstream/ 10665/69729/1/a91213.pdf.* 2007.
81. High PC, Lagasse L, Becker S, Ahlgren I, Gardner A. Literacy promotion in primary care pediatrics: Can we make a difference. *Pediatrics*. 2000;105(4):927-934.
82. Needlman R, Fried LE, Morley MA, Taylor S, Zuckerman B. Clinic-based intervention to promote literacy: A pilot study. *American Journal of Diseases of Children*. 1991;145:881-884.
83. Needleman R, Silverstein M. Pediatric Interventions to Support Reading Aloud: How Good is the Evidence. *Developmental and Behavioral Pediatrics*. 2004;25(5):352-363.
84. Golova N, Alario AJ, Vivier PM, Rodriguez M, High PC. Literacy promotion for Hispanic families in a primary care setting: a randomized, controlled trial. *Pediatrics*. 1999;103(5):993-997. doi:10.1542/peds.103.5.993
85. Mendelsohn AL, Mogilner LN, Dreyer BP, et al. The impact of clinic-based literacy intervention on language development in inner-city preschool children. *Pediatrics*. 2001;107(1):130-134. doi:10.1542/peds.107.1.130
86. Guevara JP, Erkoboni D, Gerdes M, et al. Effects of Early Literacy Promotion on Child Language Development and Home Reading Environment: A Randomized Controlled Trial. *The Journal of pediatrics*. 2020;2doi:10.1016/j.ympdx.2020.100020
87. Diener ML, Hobson-Rohrer W, Byington CL. Kindergarten readiness and performance of Latino children participating in Reach Out and Read. *Journal of Community Medical Health Education*. 2012;2(133)
88. Mendelsohn AL, Huberman HS, Berkule SB, Brockmeyer CA, Morrow LM, Dreyer BP. Primary Care Strategies for Promoting Parent-Child Interactions and School Readiness in At-Risk Families: The Bellevue Project for Early Language, Literacy, and Education Success. *Archives of Pediatric and Adolescent Medicine*. 2011;165(1):33-41.
89. Radesky JS, Carta J, Bair-Merritt M. The 30 Million-Word Gap: Relevance for Pediatrics. *JAMA Pediatrics*. Sep 1 2016;170(9):825-6. doi:10.1001/jamapediatrics.2016.1486
90. Reach Out and Read. A Forum to Launch the Next Chapter: What do we do next? Accessed March 26, 2021, https://reachoutandread.org/what-we-do/next-chapter/the-forum/
91. Zuckerman B, Needlman R. 30 Years of Reach Out and Read: Need for a Developmental Perspective. *Pediatrics*. Jun 2020;145(6)doi:10.1542/peds.2019-1958
92. Stapel-Wax J. TWMB Dissemination in Georgia. Personal Communication, Salley, B. July 2021.
93. Howell S, Snell B, Walker C, Carta J, Salley B. Universal Language Promotion in the Primary Care Setting: Implementing the Talk With Me Baby Program. presented at: Society for Research in Child Development (SRCD); 2021; Virtual Meeting.
94. Breitenstein SM, Gross D, Garvey CA, Hill C, Fogg L, Resnick B. Implementation fidelity in community-based interventions. *Research in Nursing and Health*. Apr 2010;33(2):164-73. doi:10.1002/nur.20373
95. Wang Y, Williams R, Dilley L, Houston DM. A meta-analysis of the predictability of LENA automated measures for child language development. *Developmental Review*. Sep 2020;57doi:10.1016/j.dr.2020.100921
96. Ford ALB, Elmquist M, Merbler AM, Kriese A, Will KK, McConnell SR. Toward an ecobehavioral model of early language development. *Early Childhood Research Quarterly*. 2020;50:246-258. doi:10.1016/j.ecresq.2018.11.004
97. Embry DD, Biglan A. Evidence-based kernels: Fundamental units of behavioral influence. *Clinical Child and Family Psychological Review*. 2008;11(3):75–113.
98. Kaiser AP, Roberts MY. Advances in early communication and language intervention. *Journal of Early Intervention*. 2011;33(4):298–309.

99. van de Weijer-Bergsma E, Wijnroks L, Jongmans MJ. Attention development in infants and preschool children born preterm: a review. Review. *Infant Behavior and Development*. Sep 2008;31(3):333-51. doi:10.1016/j.infbeh.2007.12.003
100. Durlak JA, DuPre EP. Implementation matters: a review of research on the influence of implementation on program outcomes and the factors affecting implementation. *American Journal of Community Psychology*. Jun 2008;41(3-4):327-50. doi:10.1007/s10464-008-9165-0
101. Harn B, Parisi D, Stoolmiller M. Balancing fidelity with flexibility and fit: What do we really know about fidelity of implementation in schools? *Council for Exceptional Children*. 2013;79(2):1818-193.
102. Slaughter SE, Hill JN, Snelgrove-Clarke E. What is the extent and quality of documentation and reporting of fidelity to implementation strategies: a scoping review. *Implement Sci*. Sep 7 2015;10:129. doi:10.1186/s13012-015-0320-3
103. Kistin C, Silverstein M. Pilot studies: A critical but potentially misued component of interventional research. *Journal of the American Medical Association*. 2015;314(15):1561-62.
104. Gilkerson J, Richards JA, Warren SF, et al. Mapping the Early Language Environment Using All-Day Recordings and Automated Analysis. *Am J Speech Lang Pathol*. May 17 2017;26(2):248-265. doi:10.1044/2016_AJSLP-15-0169
105. Cui Z, Truesdale KP, Robinson TN, et al. Recruitment strategies for predominantly low-income, multi-racial/ethnic children and parents to 3-year community-based intervention trials: Childhood Obesity Prevention and Treatment Research (COPTR) Consortium. *Trials*. May 28 2019;20(1):296. doi:10.1186/s13063-019-3418-0
106. Lefever JEB, Bigelow AE, Carta JJ, et al. Long-term impact of a cell phone-enhancing parenting intervention. *Child Maltreatment*. 2017;22(4):305-314.
107. Davis AM, Sampilo M, Gallagher KS, Landrum Y, Malone B. Treating rural pediatric obesity through telemedicine: outcomes from a small randomized controlled trial. *J Pediatr Psychol*. Oct 2013;38(9):932-43. doi:10.1093/jpepsy/jst005
108. Desouza CV, Johnson-Rabbett BE, Gajewski B, et al. The effect of nonpharmaceutical weight-loss interventions in rural patients with diabetes: RE-POWER Diabetes. *Obesity (Silver Spring)*. Apr 2022;30(4):884-892. doi:10.1002/oby.23392
109. Harris PA, Taylor R, Thieklke R, Payne J, Gonzalez N, Conde JG. Research electronic data capture (REDCap): A metadata-driven methodology and workflow process for providing translational research informatics support. *Journal of Biomedical Informatics*. 2009;42(2):377-381.
110. Pew Research Center. *Mobile Technology and Home Broadband 2021*. 2021. https://www.pewresearch.org/internet/2021/06/03/mobile-technology-and-home-broadband-2021/
111. Wang Y, Hartman M, Abdul Aziz NA, Arora S, Shi L, Tunison E. A systematic review of the use of LENA technology. *American Annals of the Deaf*. 2021;162(3):295-311.
112. Christakis DA, Gilkerson J, Richards JA, et al. Audible Television and Decreased Adult Words, Infant Vocalizations, and Conversational Turns. *Archives of Pediatric and Adolescent Medicine*. 2009;163(6):554-558.
113. Xu D, Yapanel U, Gray S. Reliability of the LENA Language Environment Analysis System in young children's natural home environment. . 2009;
114. Xu D, Yapanel U, Gray S, Baer CT. *The LENA Language Environment Analysis System: The Interpretive Time Segments (ITS) File. (LENA Foundation Technical Report LTR04-2). Retrieved from LENA Foundation: http://www.lenafoundation.org/TechReport.aspx/ITS_File/LTR-04-2*. . 2008.
115. King LS, Camacho MC, Montez DF, Humphreys KL, Gotlib IH. Naturalistic Language Input is Associated with Resting-State Functional Connectivity in Infancy. *J Neurosci*. Jan 20 2021;41(3):424-434. doi:10.1523/JNEUROSCI.0779-20.2020
116. Gomez E, Strasser K. Language and socioemotional development in early childhood: The role of conversational turns. *Dev Sci*. Sep 2021;24(5):e13109. doi:10.1111/desc.13109

117. Hoffman L, Hersey A, Tucker R, Vohr B. Randomised control language intervention for infants of adolescent mothers. *Acta Paediatrics*. Dec 2020;109(12):2604-2613. doi:10.1111/apa.15261
118. Suskind D, Leffel KR, Hernandez MW, et al. An Exploratory Study of "Quantitative Linguistic Feedback". *Communication Disorders Quarterly*. 2013;34(4):199-209. doi:10.1177/1525740112473146
119. Marchman VA, Weisleder A, Hurtado N, Fernald A. Accuracy of the Language Environment Analyses (LENA(TM)) system for estimating child and adult speech in laboratory settings. *J Child Lang*. May 2021;48(3):605-620. doi:10.1017/S0305000920000380
120. Beecher CC, Van Pay CK. Investigation of the effectiveness of a community-based parent education program to engage families in increasing language interactions with their children. *Early Childhood Research Quarterly*. 2020;53:453-463. doi:10.1016/j.ecresq.2020.04.001
121. Gilkerson J, Richards JA, Topping K. Evaluation of a LENA-Based Online Intervention for Parents of Young Children. *Journal of Early Intervention*. 2017;39(4):281-298. doi:doi/10.1177/1053815117718490
122. Adams KA, Marchman VA, Loi EC, Ashland MD, Fernald A, Feldman HM. Caregiver Talk and Medical Risk as Predictors of Language Outcomes in Full Term and Preterm Toddlers. *Child Dev*. Sep 2018;89(5):1674-1690. doi:10.1111/cdev.12818
123. Caskey M, Stephens B, Tucker R, Vohr B. Adult talk in the NICU with preterm infants and developmental outcomes. *Pediatrics*. Mar 2014;133(3):e578-84. doi:10.1542/peds.2013-0104
124. Caskey M, Stephens B, Tucker R, Vohr B. Importance of parent talk on the development of preterm infant vocalizations. *Pediatrics*. Nov 2011;128(5):910-6. doi:10.1542/peds.2011-0609
125. The Security Rule. Online. Accessed June 12, 2017. 126. The HIPAA Privacy Rule. Online. Accessed June 12, 2017.
